# Supplementary material for: Minute-timescale free-energy calculations reveal a pseudo-active state in the adenosine A2A receptor activation mechanism
Source: Chem. 2024 Dec 12;10(12):3678–98. doi: 10.1016/j.chempr.2024.08.004 (PMC11965979; doi:10.1016/j.chempr.2024.08.004)
Supplement: Document S1. Figures S1–S23 and Tables S1–S3 [file mmc1.pdf]

Chem, Volume *10*

Supplemental information

Minute-timescale free-energy calculations reveal a pseudo-active state in the adenosine A_{2A} receptor activation mechanism

Vincenzo Maria D'Amore, Paolo Conflitti, Luciana Marinelli, and Vittorio Limongelli

Supplemental Information

TABLE OF CONTENTS

Table S1: Conformational features of main $A_{2A}R$ states

Table S2: List of atoms selected for $_{ACT}P$ collective variable

Table S3: List of atoms selected for $_{TM6}P$ collective variable

Figure S1: RMSD plots of the $A_{2A}R$ orthosteric binding site.

Figure S2: RMSD plots of the $A_{2A}R$ intracellular binding site

Figure S3: Pearson Coefficient Matrices and Protein Structure Network

Figure S4: RMSD plots of the $A_{2A}R$ transmembrane helices

Figure S5: RMSD plots of the $A_{2A}R$ connector region

Figure S6: Stability of the pseudo-active $A_{2A}R$ in MD simulations

Figure S7: Comparison of the TM6 translational movement in different $A_{2A}R$ structures

Figure S8: Plot of the IBS's volume in the FApo system

Figure S9: Violin plots of the $H^{6.32}$-$R^{7.56}$ and $H^{6.32}$-$R^{8.48}$ cation-π interactions

Figure S10: Activating Ionic Lock (AIL) formation in rhodopsin and adenosine receptors

Figure S11: Graphical representations of the path collective variables (PCVs)

Figure S12: Convergence plots of the free-energy calculation in NECA-bound $A_{2A}R$

Figure S13: Convergence plots of the free-energy calculation in the apo $A_{2A}R$

Figure S14: Convergence plots of the free-energy calculation in the ZMA-bound $A_{2A}R$

Figure S15: RMSD plots of the energy minima receptor structures during unbiased MD simulations

Figure S16: FESs as a function of $_{ACT}P.s$ and $_{TM6}P.s$

Figure S17: Protein-Protein docking results

Figure S18: Targeted and unbiased MD calculations on $A_{2A}R$/G Protein complex

Figure S19: pAs-$A_{2A}R$/G protein binding mode

Figure S20: Structural stability of the pAs-$A_{2A}R$/β-arrestin 1 complex

Figure S21: Stability of the Activating Ionic Lock (AIL) interaction

Figure S22: Projection of $A_{2A}R$ experimental structures onto the FES of the $A_{2A}R$/NECA system

Figure S23: Comparison between ^{19}F-NMR spectra and free energy surfaces of A2AR

Table S1: Conformational microswitches in the diverse $A_{2A}R$ states

	Microswitch			
**State**	NPxxY		AIL	IIL
A	1.4	3.7	3.9	20.2
pAs	2.0	4.0	4.3	17.6
Intermediate Active	3.4	4.5	12.9	11.8
I1	3.9	1.9	16.0	4.0
I2	3.9	1.7	16.4	7.0
	RMSD (Å)[1]	RMSD (Å)[2]	Distance (Å)[3]	Distance (Å)[3]

[1] The RMSD was computed for the C-α of the transmembrane helices after with respect to $A_{2A}R$ active conformation (PDB code:5G53)

[2] The RMSD was computed for the C-α of the transmembrane helices after with respect to $A_{2A}R$ active conformation (PDB code: 3PWH)

[3] The values of AIL and IIL correspond to the distances of E6.30-Cδ from R5.66-Cζ and R3.50-Cɣ, respectively.

Table S2: List of atoms used for the alignment and RMSD measurement of $_{ACT}$Path CV (residues numbered according to UNIPROT ID P29274 numeration)

RMSD computation		Alignment	
**Atom**	**Residue**	**Atom**	**Residue**
Cα and Cβ	92	Cα	19-29
Cα and Cβ	189	Cα	44-64
Cα and Cβ	201	Cα	84-89
Cα and Cβ	205	Cα	119-138
Cα and Cβ	226-232	Cα	181-186
Cα and Cβ	234-236	Cα	246-251
Cα and Cβ	240-250	Cα	272-277
Cα and Cβ	284-289		
Cγ	208		
Cγ	225		
Cζ	202		
Cζ	288		

Table S3: List of contacts used for computing of $_{TM6}$Path CV (residues numbered according to UNIPROT ID P29274 numeration).

Contact List					
**Contact number**	**Atom i**	**Atom j**	**R_0**	***n***	***m***
1	Glu228-Cδ	Arg205-Cζ	7Å	8	16
2	Glu228-Cδ	Arg102-Cζ	7Å	8	16
3	Glu228-Cδ	Asp101-Cγ	16.5Å	8	18
4	Arg102-Cζ	Asp97-Cγ	7.5Å	6	18
5	Leu235- Cγ	Leu198-Cγ	9Å	8	16
6	Leu235- Cγ	Ile98-Cβ	10Å	10	24
7	Tyr288-Cζ	Tyr197-Cζ	8.5Å	8	28
8	Tyr288-Cζ	Val45-Cζ	6.7Å	12	24

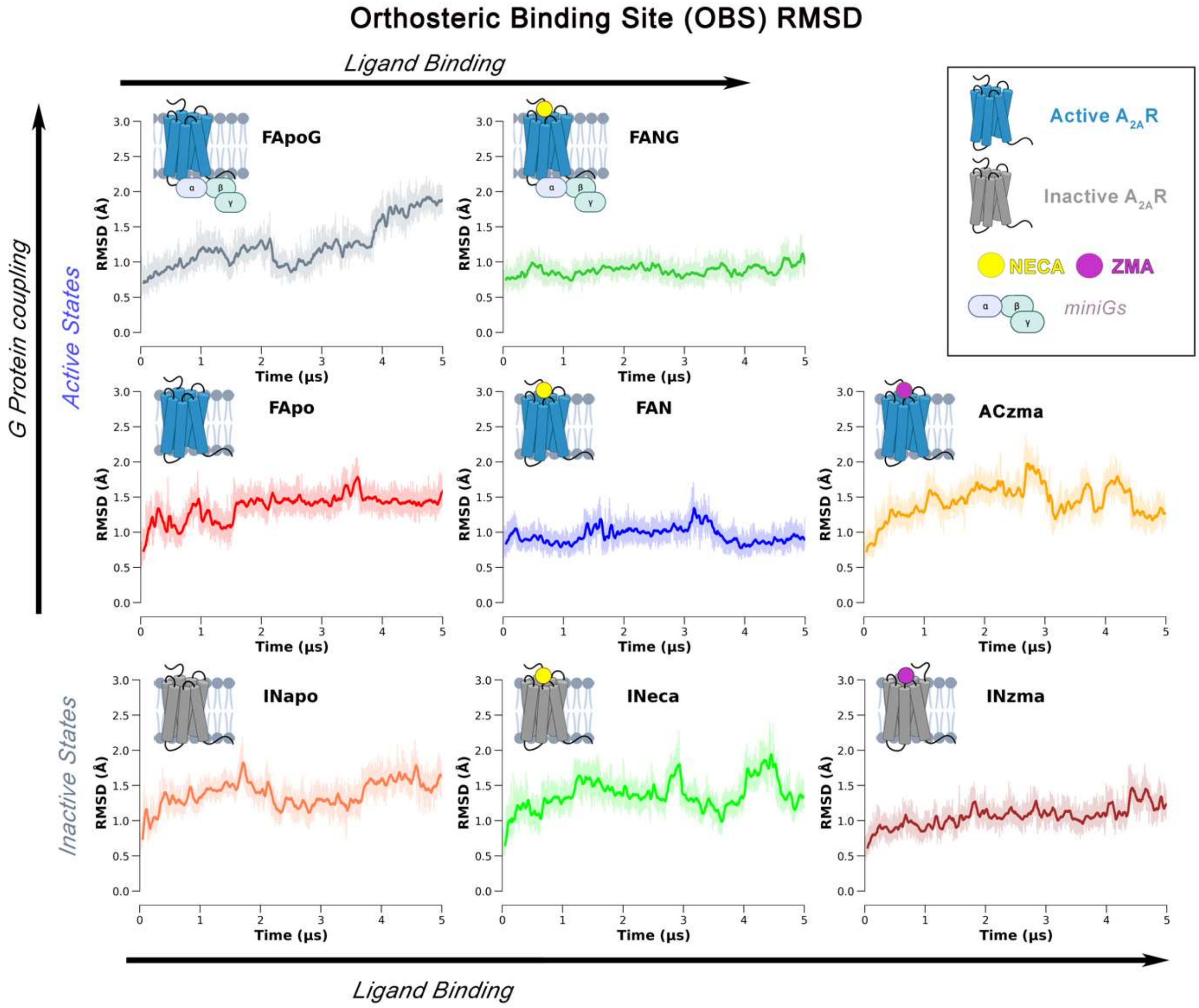

Figure S1. RMSD plots of the A2AR **orthosteric binding site**. The RMSD plots were computed using the Cα atoms of residues 8-14, 57-66, 75-90, 132-140, 175-187, 245-258, 267-278 (residue numbering as in UNIPROT ID P29274) with respect to the first frame of each trajectory. The bolded lines show a RMSD value smoothed with a rolling window of 5 ns, whereas the actual fluctuations are shown with a slight transparency.

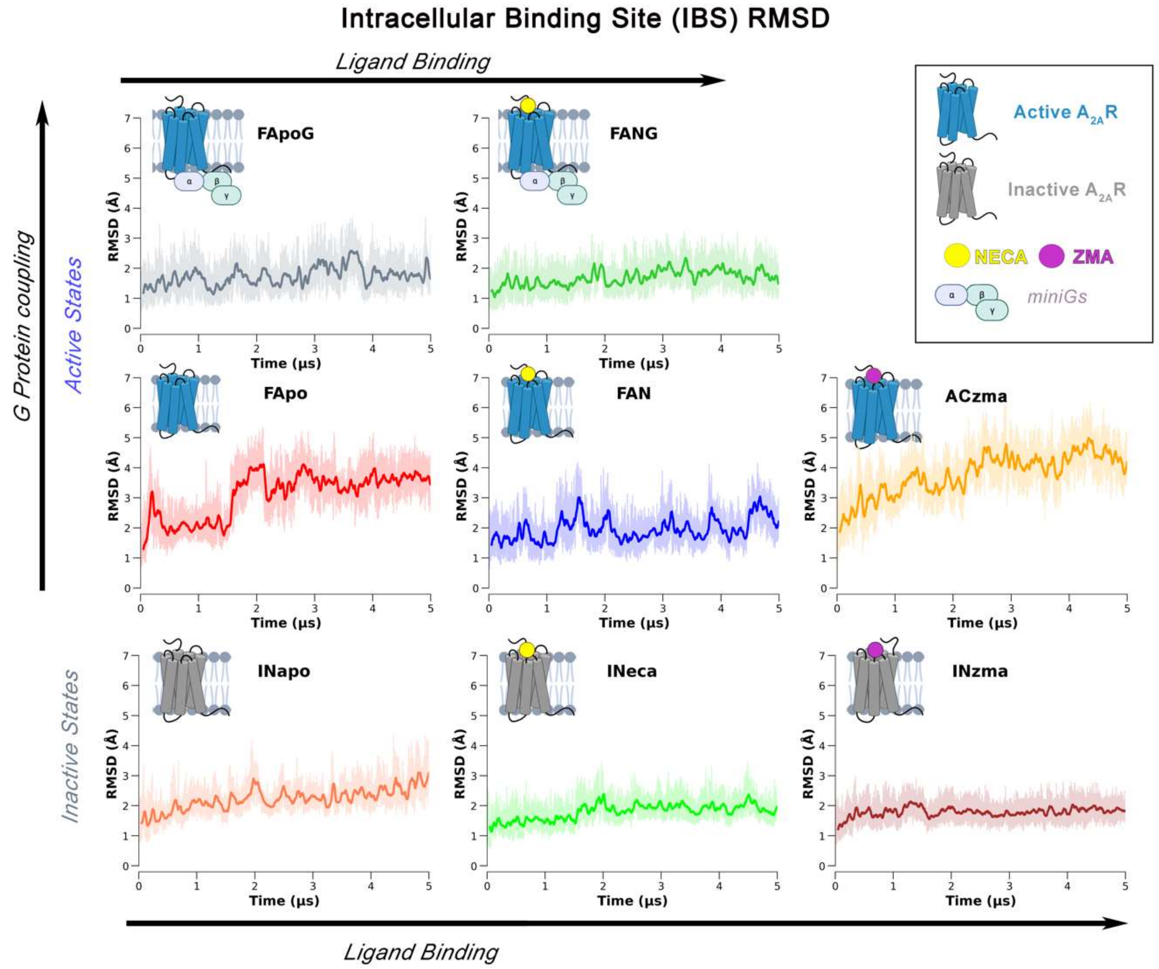

Figure S2. RMSD plots of the A2AR **intracellular binding site**. The RMSD plots were computed using the IBS Cα atoms of residues 24-31, 42-49, 98-106, 119-126, 198-208, 222-235, 283-287 (residue numbering as in UNIPROT ID P29274) with respect to the first frame of each trajectory. The bolded lines show a RMSD value smoothed with a rolling window of 5 ns, whereas the actual fluctuations are shown with a slight transparency.

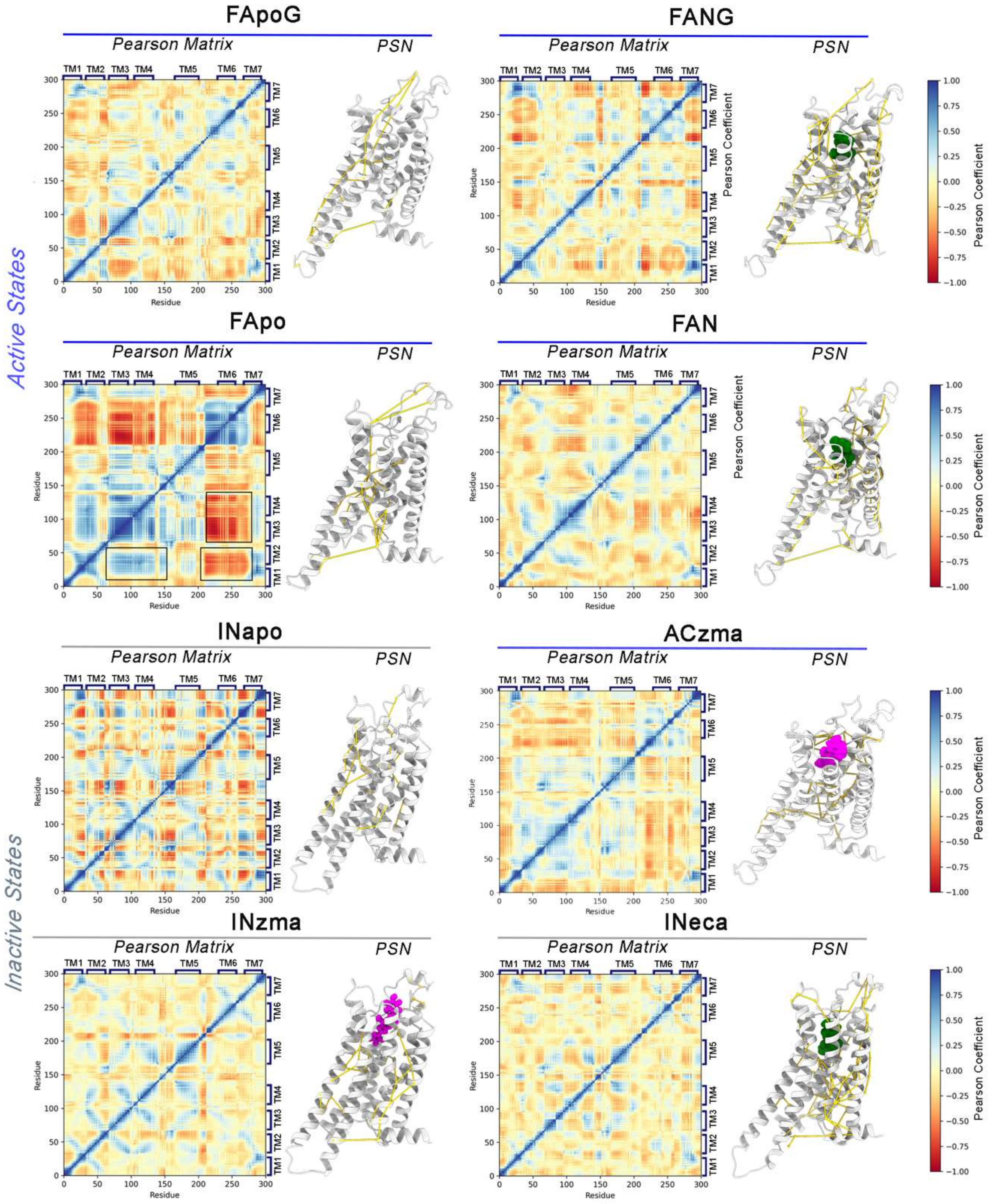

Figure S3. Pearson Coefficient Matrices and Protein Structure Network. The Pearson Coefficients were computed based on the corresponding elements of the covariance matrix of the atomic (Cα of each residue) fluctuations during the unbiased trajectories. The PSN were obtained with the aid of the webpsn server; the links connecting the most important aminoacidic hubs in the global metapath are depicted as gold connections and projected onto a cartoon representation of the starting $A_{2A}R$ conformation in the specific MD run.

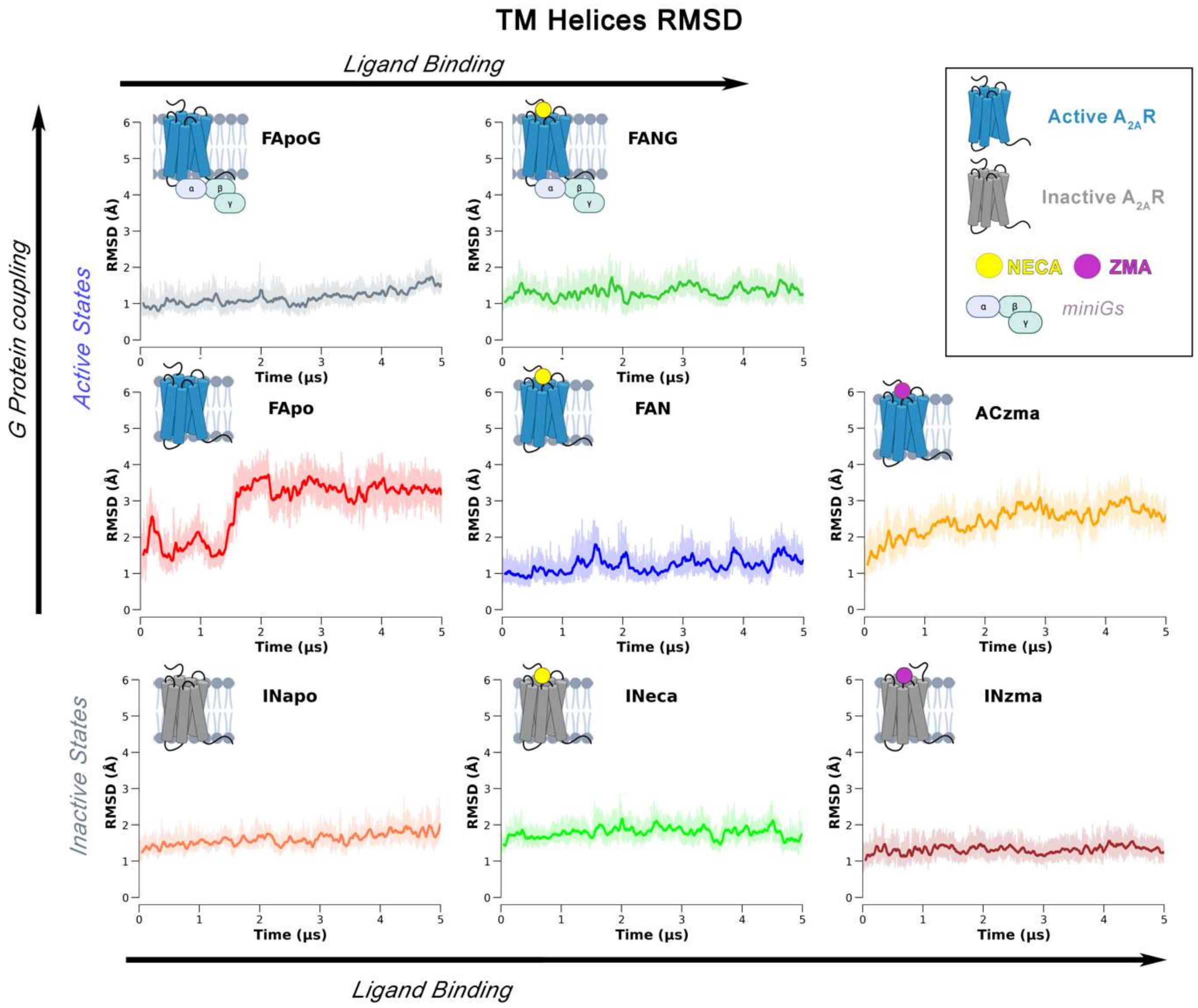

Figure S4. RMSD plots of the A2AR **transmembrane helices**. The RMSD plots were computed using the Cα atoms of residues 8-31, 42-66, 75-106, 119-140, 175-205, 227-257, 267-287 (residue numbering as in UNIPROT ID P29274) with respect to the first frame of each trajectory. The bolded lines show a RMSD value smoothed with a rolling window of 5 ns, whereas the actual fluctuations are shown with a slight transparency.

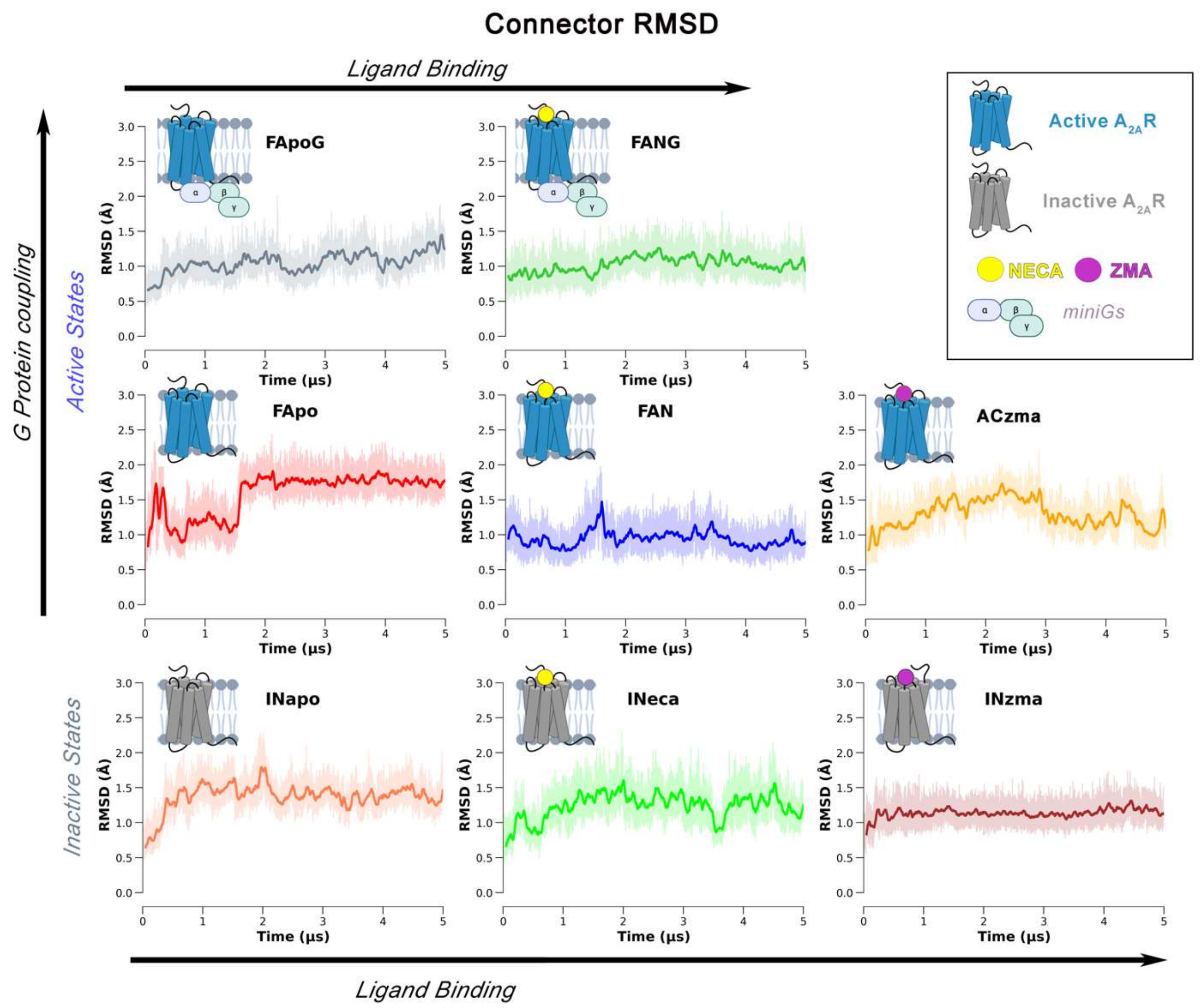

Figure S5. RMSD plots of the A2AR **connector region.** The RMSD plots were computed using the Cα atoms of residues 15-23, 50-56, 91-97, 127-131, 188-197, 236-244, 279-283 (residue numbering as in UNIPROT ID P29274) with respect to the first frame of each trajectory. The bolded lines show a RMSD value smoothed with a rolling window of 5 ns, whereas the actual fluctuations are shown with a slight transparency.

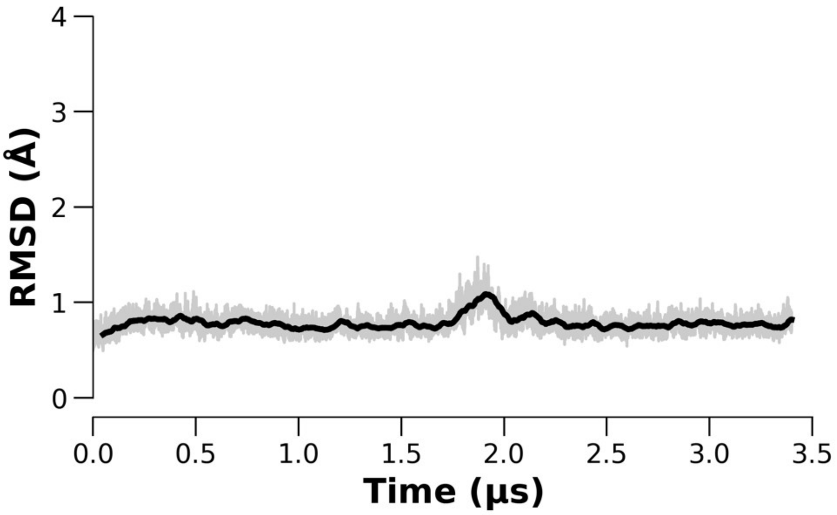

Figure S6. Stability of the pseudo-active A2AR in MD simulations. RMSD plots were computed for the GPCR transmembrane helices (Cα atoms of residues 8-31, 42-66, 75-106, 119-140, 175-205, 227-

257, 267-287; residue numbering as in UNIPROT ID P29274) with respect to the first frame after the conformational transition observed in the *FApo* system. The bolded lines show a RMSD value smoothed with a rolling window of 5 ns, whereas the actual fluctuations are shown with a slight transparency.

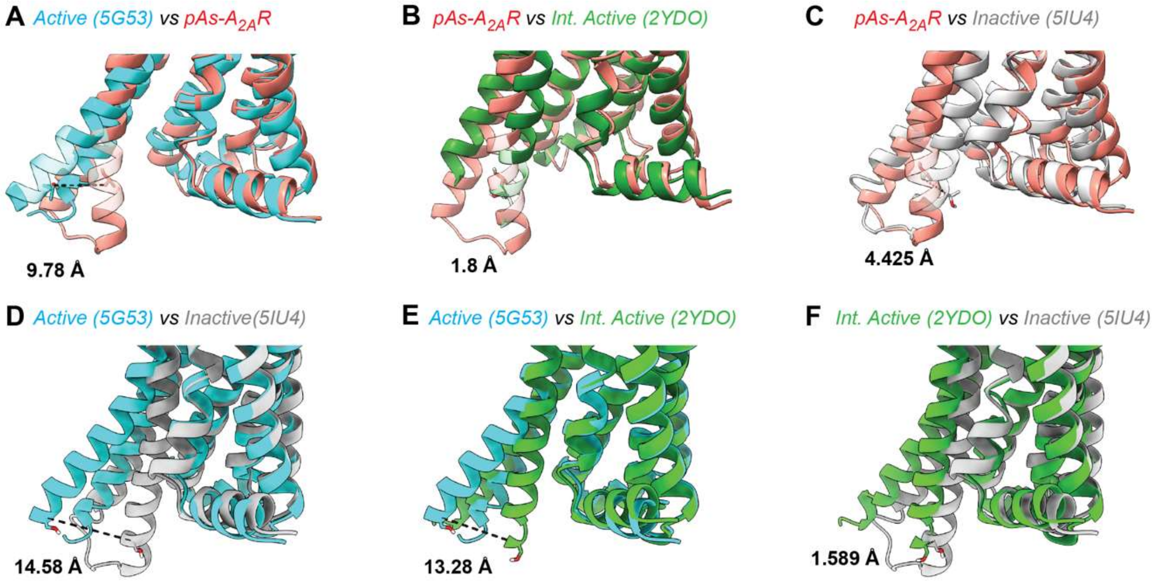

Figure S7. Comparison of the TM6 translational movement in different $A_{2A}R$ structures. Superposition between the intracellular section of pAs-$A_{2A}R$ (salmon) with the experimental active (cyan, PDB code 5G53), the intermediate-active (green, PDB code 2YDO), and the inactive structures (gray, PDB code 5IU4). The displacement of the cytoplasmic end of TM6 is measured as the distance between the Cα atoms of Thr224$^{6.26}$ in the different conformations (threonine is displayed as sticks).

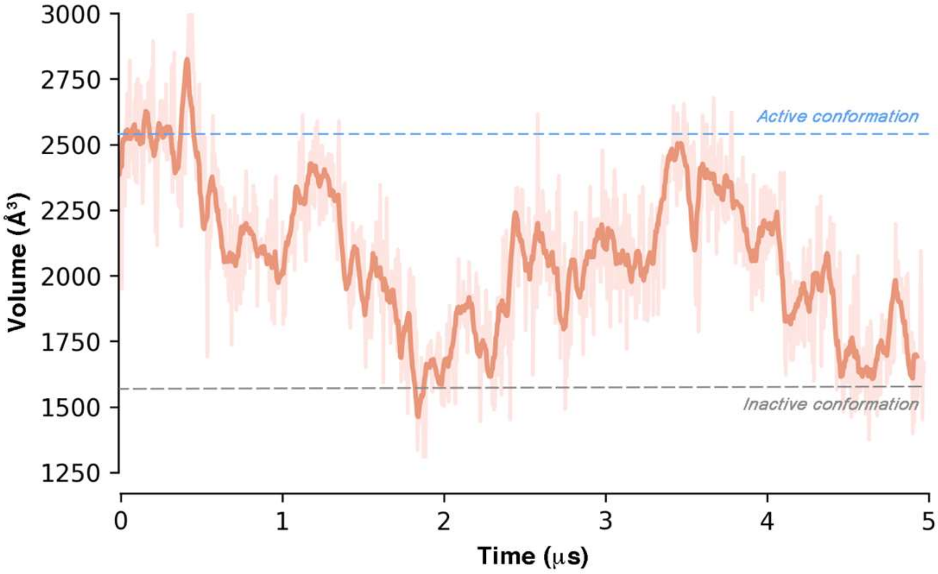

Figure S8. Plot of the IBS's volume in the FApo system. The bolded lines show a volume value smoothed with a rolling window of 5ns, whereas the actual fluctuations are shown with a slight transparency. The reference volume values for $A_{2A}R$ active and inactive states are shown as light-blue and grey dashed lines, respectively. The volume computation was performed with the aid POVME.py tool (J. Chem. Theory Comput. 2017, 13, 9, 4584–4592) by setting an inclusion sphere of 16Å centered at the center of mass of residues 24-31, 42-49, 98-106, 119-126, 198-208, 222-235, 283-287; residue numbering as in UNIPROT ID P29274.

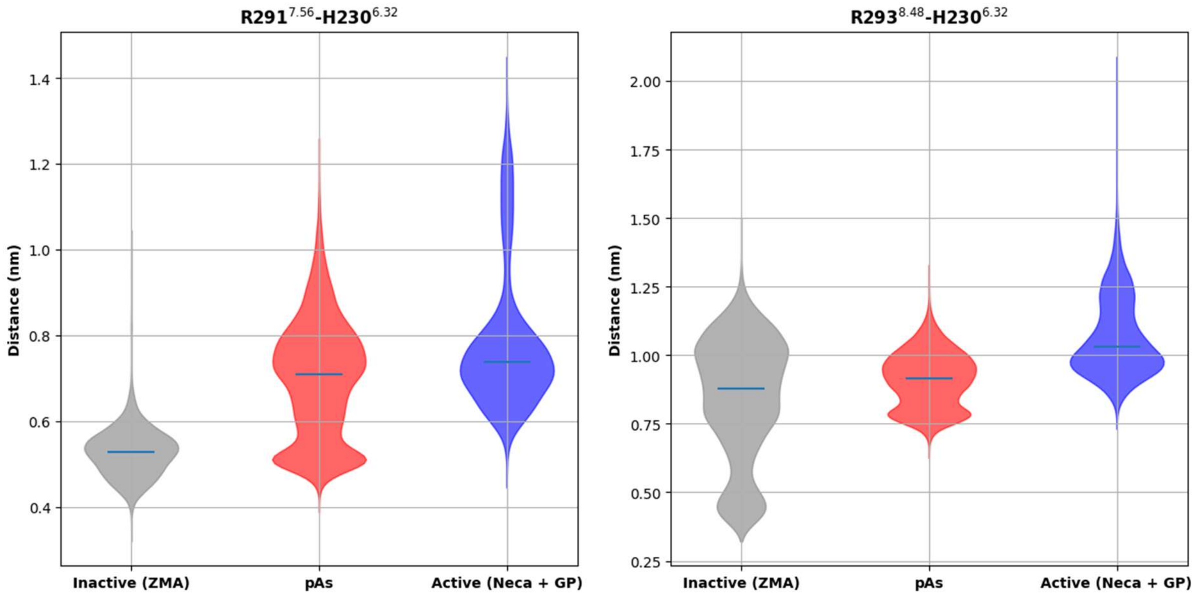

Figure S9. Violin plots of the H$^{6.32}$-R$^{7.56}$ and H$^{6.32}$-R$^{8.48}$ **cation-π interactions.** The plots are computed for the residue pairs H$^{6.32}$-R$^{7.56}$ (Left) and H$^{6.32}$-R$^{8.48}$ (Right) over the unbiased MD trajectories of $A_{2A}R$ in the inactive (in complex with ZMA), pAs, and active (in complex with NECA e G Protein) states. Distances are measured between the center of the imidazole ring of histidine and the Cζ of arginine residues.

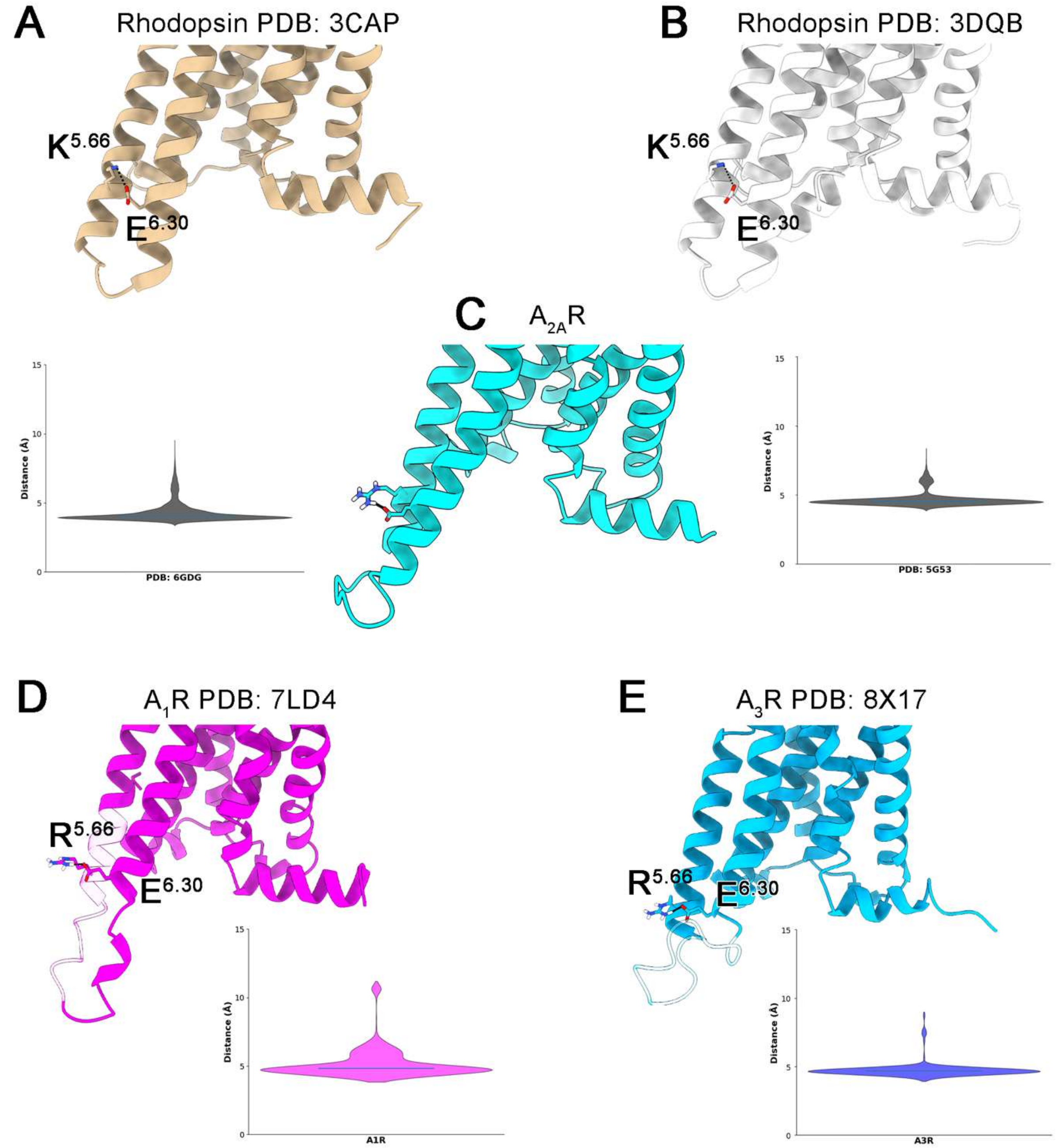

Figure S10. Activating Ionic Lock (AIL) formation in rhodopsin and adenosine receptors. Atomistic detail of the TM5-TM6 salt bridge interaction observed between K/$R^{5.66}$ and $E^{6.30}$ in the experimental active structures of rhodopsin with PDB codes 3CAP (A) and 3DQB (B), and in the $A_{2A}R$ (C), A_1R (D) and A_3R (E) receptors during MD simulations. The violin plots computed for the distance between $R^{5.66}$'s Cδ and $E^{6.30}$'s Cζ are shown as insets.

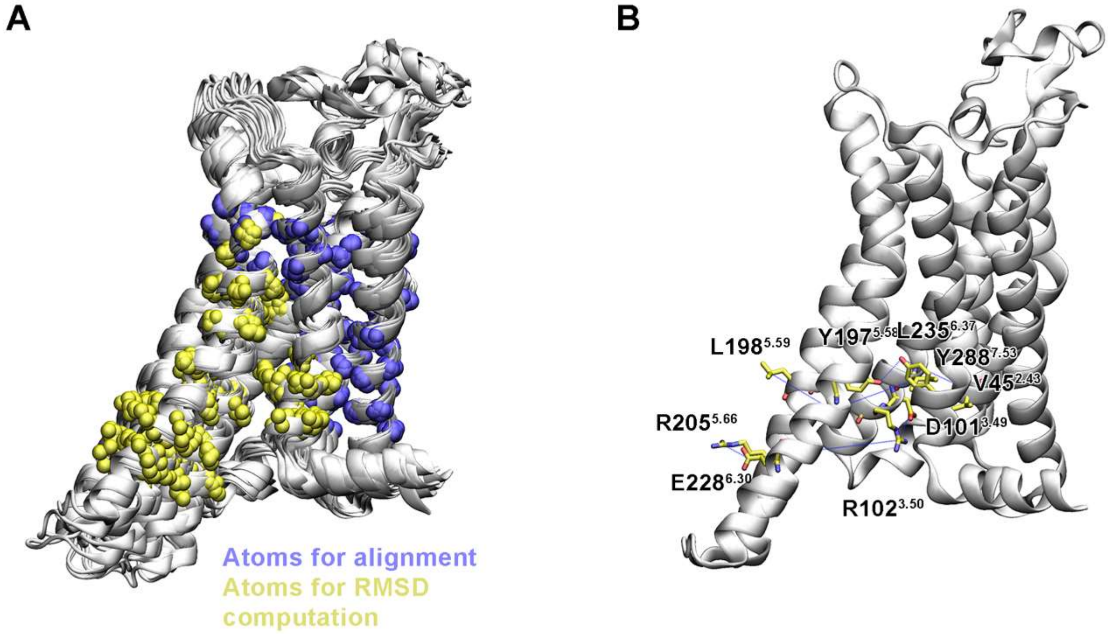

Figure S11. Graphical representations of the path collective variables (PCVs). A) Cartoon representation of the 12 frames forming the reference path used for the $_{ACT}$ Path Collective Variable. The RMSD matrix was computed for the atoms shown as yellow sphere and listed in Table S1, after alignment of the frames based on the position of the Cα and Cβ atoms depicted as blue spheres. B) Graphical representation of the seven contacts (blue dashed lines) used for defining the $_{TM6}$Path Collective variable in the CMAP space, which are listed in Table S2. For sake of clarity the contacts are projected on the first of the nine frames forming the path, corresponding to the receptor active conformation.

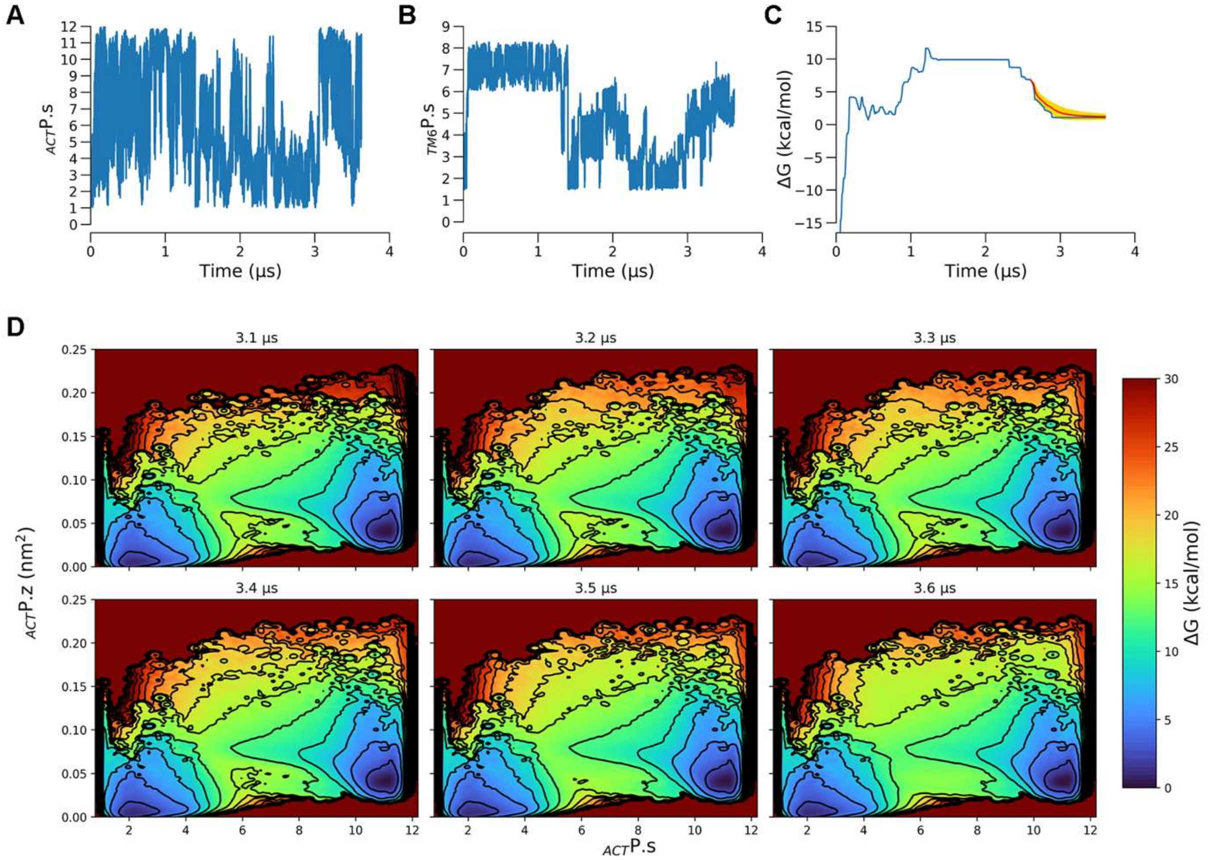

Figure S12. Convergence plots of the free-energy calculation in NECA-bound A_{2A}R. A) Time evolution of the $_{ACT}$P.s collective variable biased during the simulation. B) Time evolution of the $_{TM6}$P.s collective variable biased during the simulation. C) Free energy difference between the two main energy basins (A* and I*) as a function of the simulation time (blue). Its time-weighted average is reported in red, error bars are shown in yellow. D) Time evolution of the reweighted FES as a function of the $_{ACT}$P.s and $_{ACT}$P.z CVs during the last 500 ns of simulation.

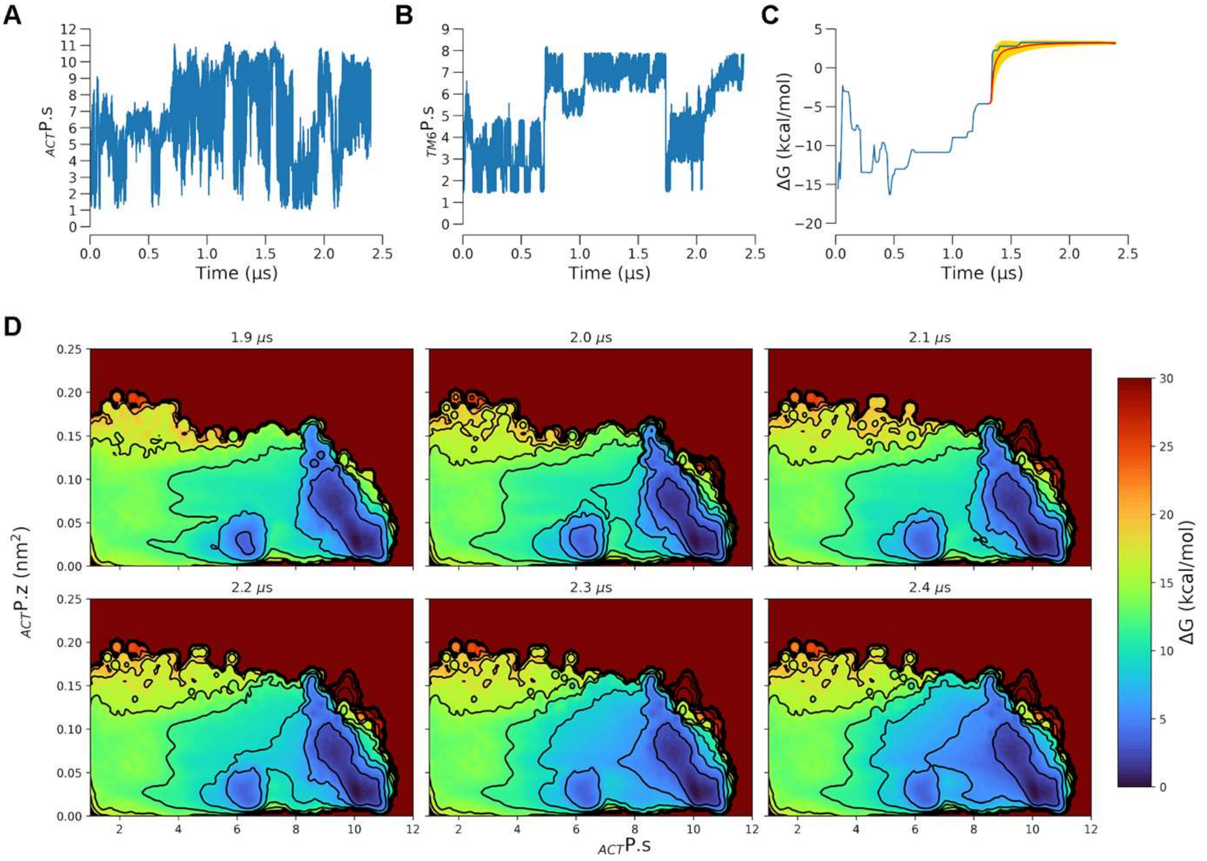

Figure S13. Convergence plots of the free-energy calculation in the apo A_{2A}R. A) Time evolution of the $_{ACT}$P.s collective variable biased during the simulation. B) Time evolution of the $_{TM6}$P.s collective variable biased during the simulation. C) Free energy difference between the two main energy basins (A* and I*) as a function of the simulation time (blue). Its time-weighted average is reported in red, error bars are shown in yellow. D) Time evolution of the reweighted FES as a function of the $_{ACT}$P.s and $_{ACT}$P.z CVs during the last 500 ns of simulation.

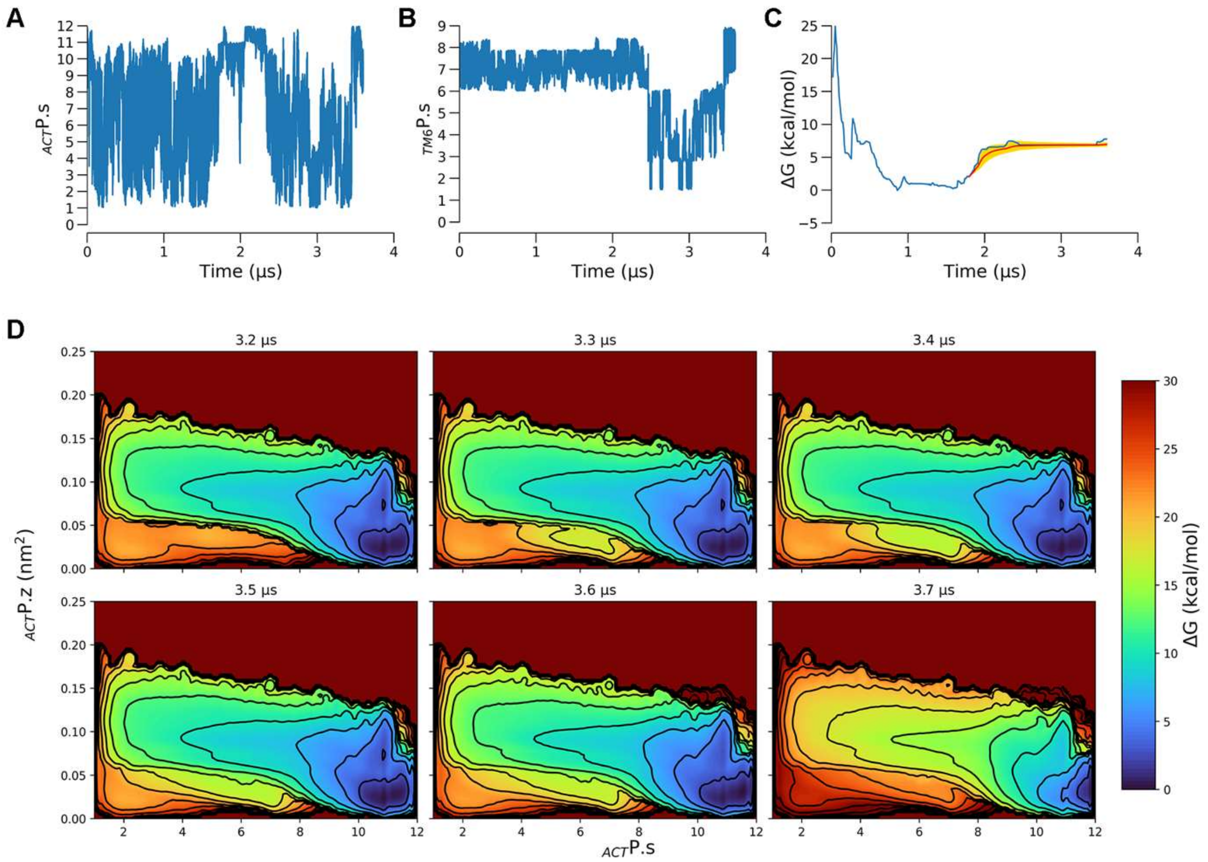

Figure S14. Convergence plots of the free-energy calculation in the ZMA-bound A_{2A}R. A) Time evolution of the $_{ACT}$P.s collective variable biased during the simulation. B) Time evolution of the $_{TM6}$P.s collective variable biased during the simulation. C) Free energy difference between the two main energy basins (A* and I*) as a function of the simulation time (blue). Its time-weighted average is reported in red, error bars are shown in yellow. D) Time evolution of the reweighted FES as a function of the $_{ACT}$P.s and $_{ACT}$P.z CVs during the last 500 ns of simulation.

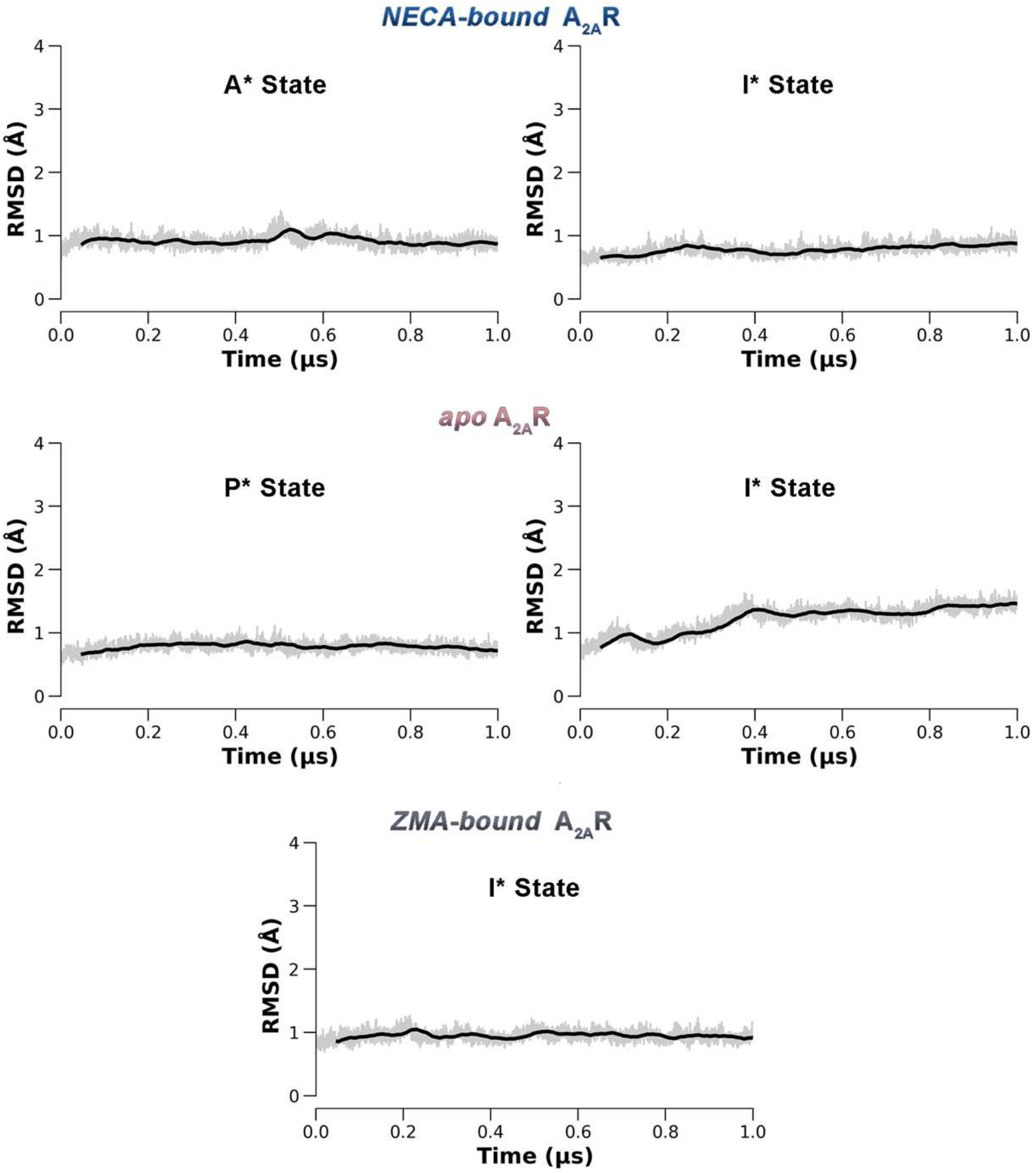

Figure S15. RMSD plots of the energy minima receptor structures during unbiased MD simulations. RMSD plots are computed for the Cα atoms of the TM helices (Cα atoms of residues 8-31, 42-66, 75-106, 119-140, 175-205, 227-257, 267-287) with respect to the starting conformation of each minimum. For the A state in the *NECA-bound* system and I state in the *ZMA-bound* system, the plots refer to the first 1 μs MD simulations performed on the X-ray structures and also described in Supplementary Fig.1-4. For the P state in the *apo* system, the plot refers to the first 1 μs after the conformational transition observed in the *FApo* MD system described in Supplementary Fig. 1-4. The bolded lines show a RMSD value smoothed with a rolling window of 5 ns, whereas the actual fluctuations are shown with a slight transparency.

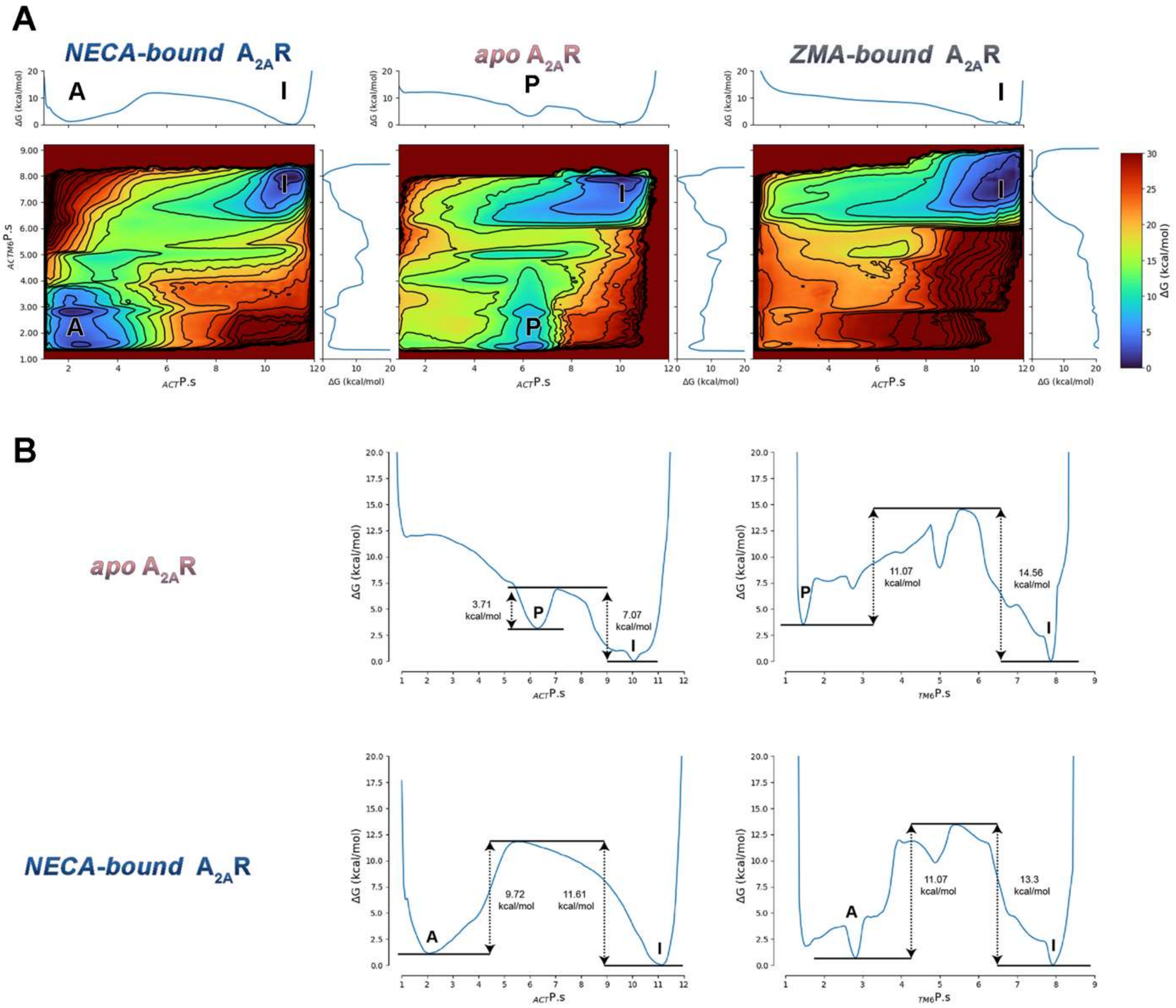

Figure S16. FESs as a function of $_{ACT}$P.s and $_{TM6}$P.s. A) Activation Free Energy landscapes of the *NECA-bound*, *apo* and *ZMA-bound* forms of $A_{2A}R$ as a function of the $_{ACT}$P.s and $_{TM6}$P.s collective variables. Isosurfaces are displayed every 3 kcal/mol. B) 1-D free energy profiles of the apo and the NECA-bound A_{2A} calculated as a function of the $_{ACT}$P.s (left) and the $_{TM6}$P.s (right) CVs.

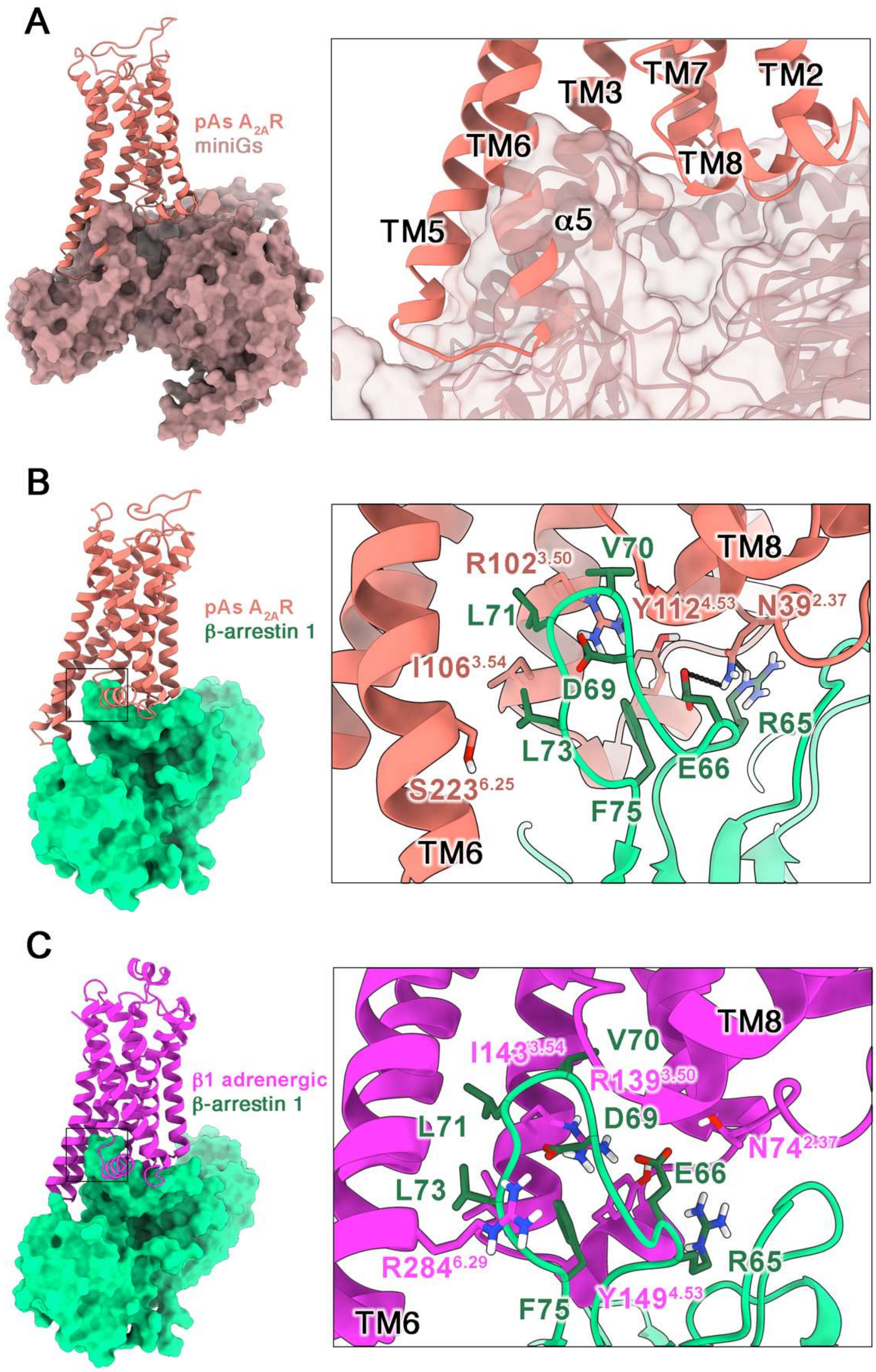

Figure S17. Protein-Protein docking results. A) Steric clash between the intracellular portion of $A_{2A}R$ and the α5 helix of Gα subunit in the the pAs/G protein complex obtained by manual fitting. B) Docking complex predicted by Haddock of the pAs-$A_{2A}R$/β-arrestin 1 interaction. The pAs $A_{2A}R$ structure is shown as salmon cartoon, whereas the mini Gs and β-arrestin 1 are depicted (cartoon and surface) in brown and green, respectively. C) Experimental Cryo-EM structure of β arrestin 1 (green surface and cartoon) at the β1 adrenoreceptor IBS (purple cartoon, PDB code: 6TKO).

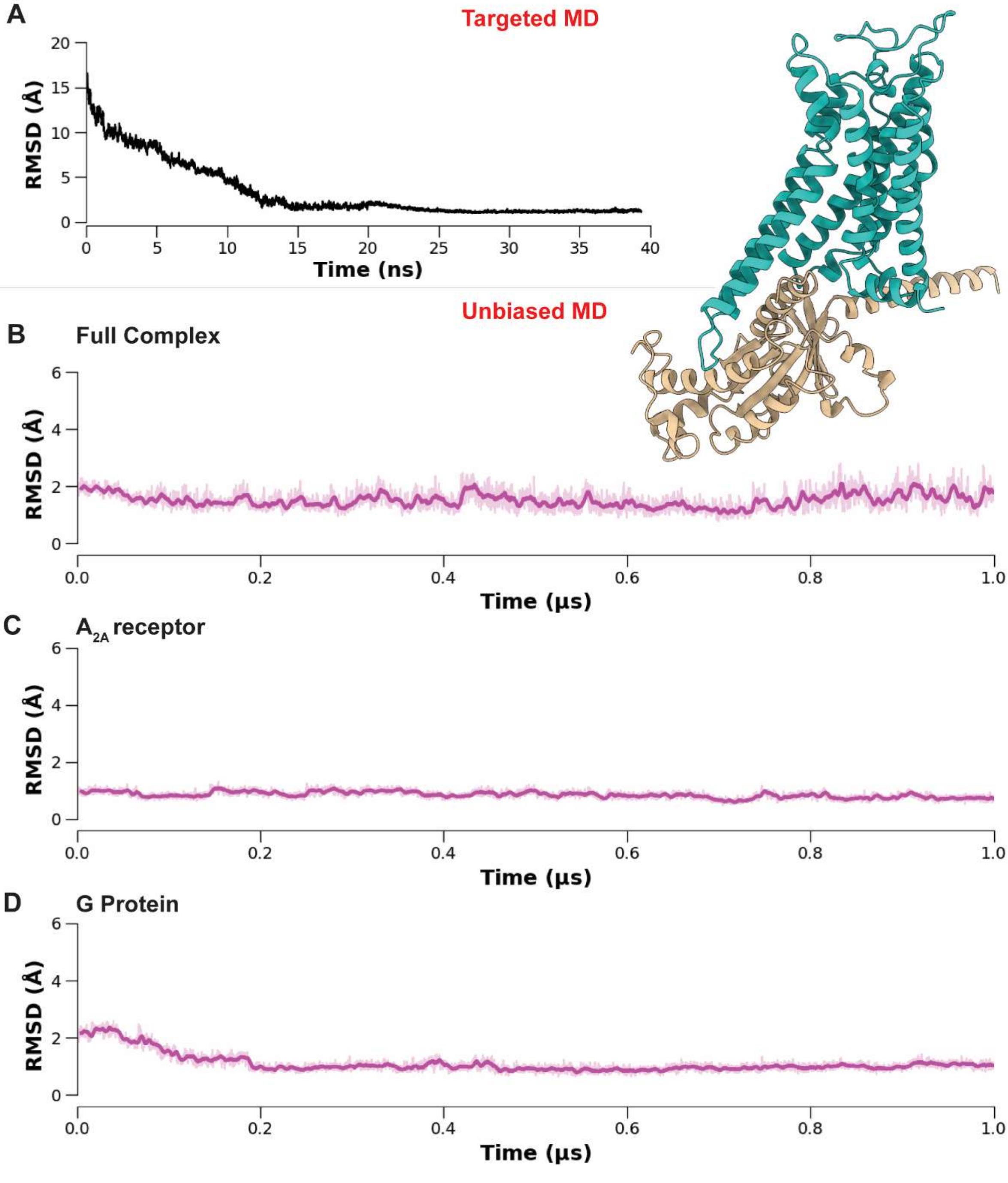

Figure S18. Targeted and unbiased MD calculations on A_{2A}R/G Protein complex. A) Evolution of the RMSD of the G protein secondary structure's Cβ calculated relative to the experimental A_{2A}R-miniGs complex (PDB: 6GDG) during 40 ns targeted MD simulations. B-D) Stability of the A_{2A}R-miniGs complex throughout 1 μs unbiased MD simulation. Evolution of the RMSD of (B) the binary complex, (C) the A_{2A}R receptor, and (D) the G protein during the unbiased MD calculated relative to the average protein conformation.

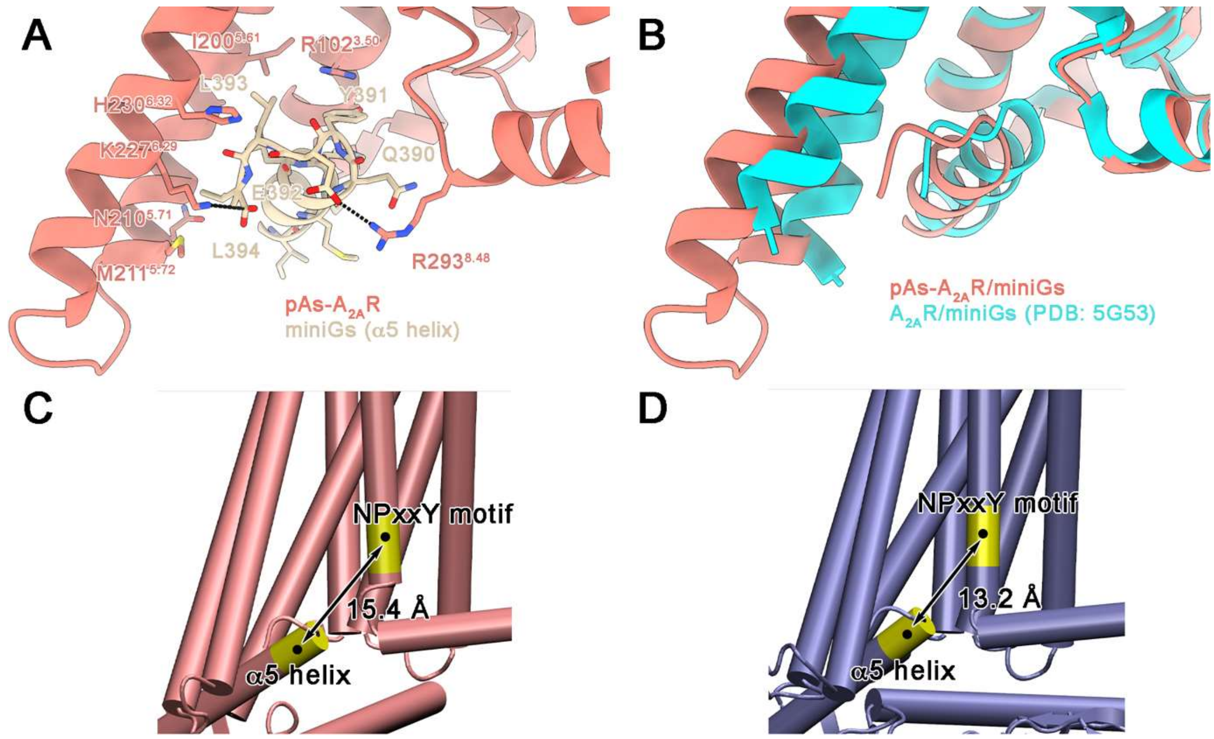

Figure S19. pAs-A$_{2A}$R/G protein binding mode. A) Binding mode of the G$_{\alpha}$'s Cα5 helix (yellow) at the intracellular binding site of A$_{2A}$R pAs (salmon) identified by molecular dynamics simulations. B) Superimposition of the pAs-G protein complex (salmon) with the experimental structure with PDB code 5G53 (light blue). C-D) Distance between the A$_{2A}$R NPxxY motif and the G$_{\alpha}$'s Cα5 helix measured in the pAs/G protein and the experimental A$_{2A}$R/G protein complexes, respectively.

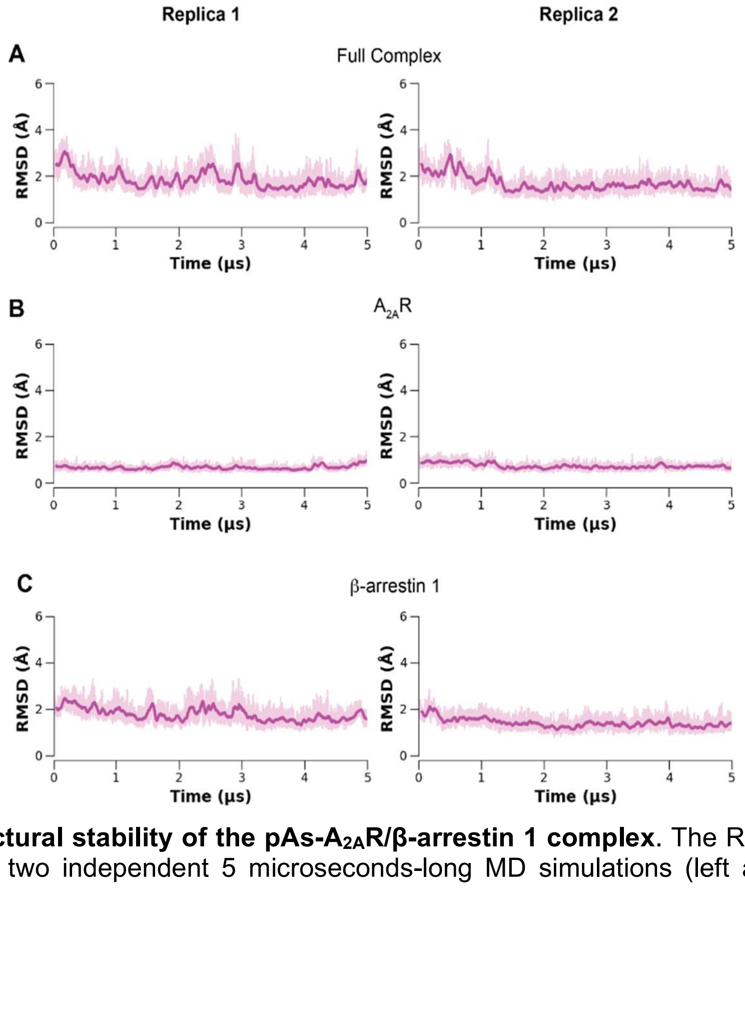

Figure S20. Structural stability of the pAs-A$_{2A}$R/β-arrestin 1 complex. The RMSD values were computed over the two independent 5 microseconds-long MD simulations (left and right column,

respectively) using the Cα atoms of the entire complex (A), the A_{2A} receptor (B) and β-arrestin with respect to their average conformation. The bolded lines show a RMSD value smoothed with a rolling window of 5 ns, whereas the actual fluctuations are shown with a slight transparency.

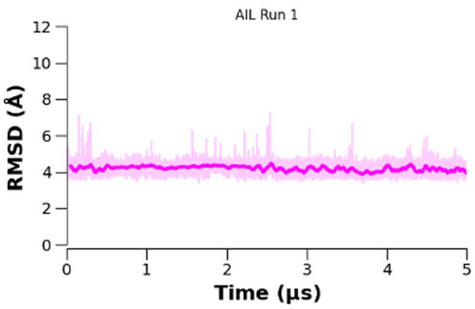

Figure S21. Stability of the Activating Ionic Lock (AIL) interaction. The RMSD values were computed over the two independent 5 microseconds-long MD simulations (left and right column, respectively) on the predicted pAs-A_{2A}R/β-arrestin 1 complex. The AIL is computed as distance between the Cδ of $E^{6.30}$ and the Cz of $R^{5.66}$. The bolded lines show a RMSD value smoothed with a rolling window of 5 ns, whereas the actual fluctuations are shown with a slight transparency.

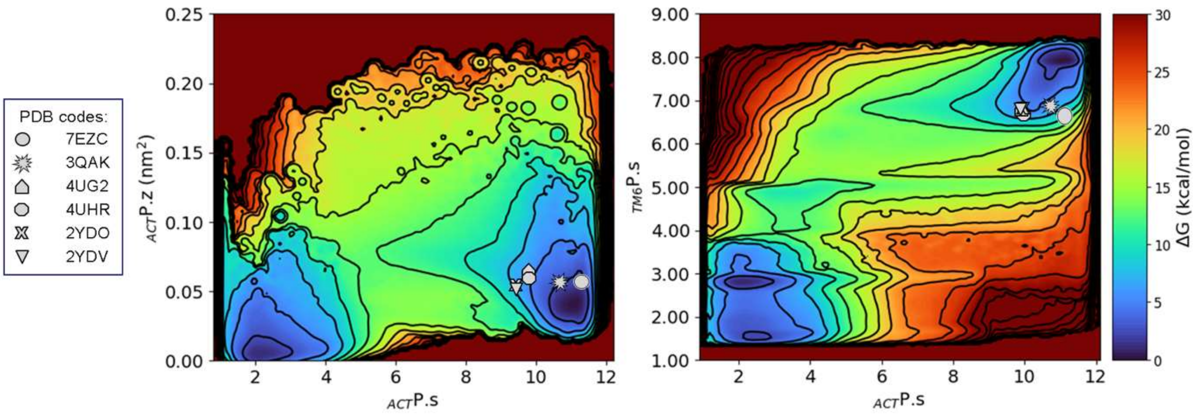

Figure S22. Projection of A_{2A}R experimental structures onto the FES of the A_{2A}R/NECA system. Projection of all the agonist-bound experimental structure (gray shapes) of the *uncoupled* A_{2A}R on the activation free energy surfaces predicted in the present work for the NECA-bound system.

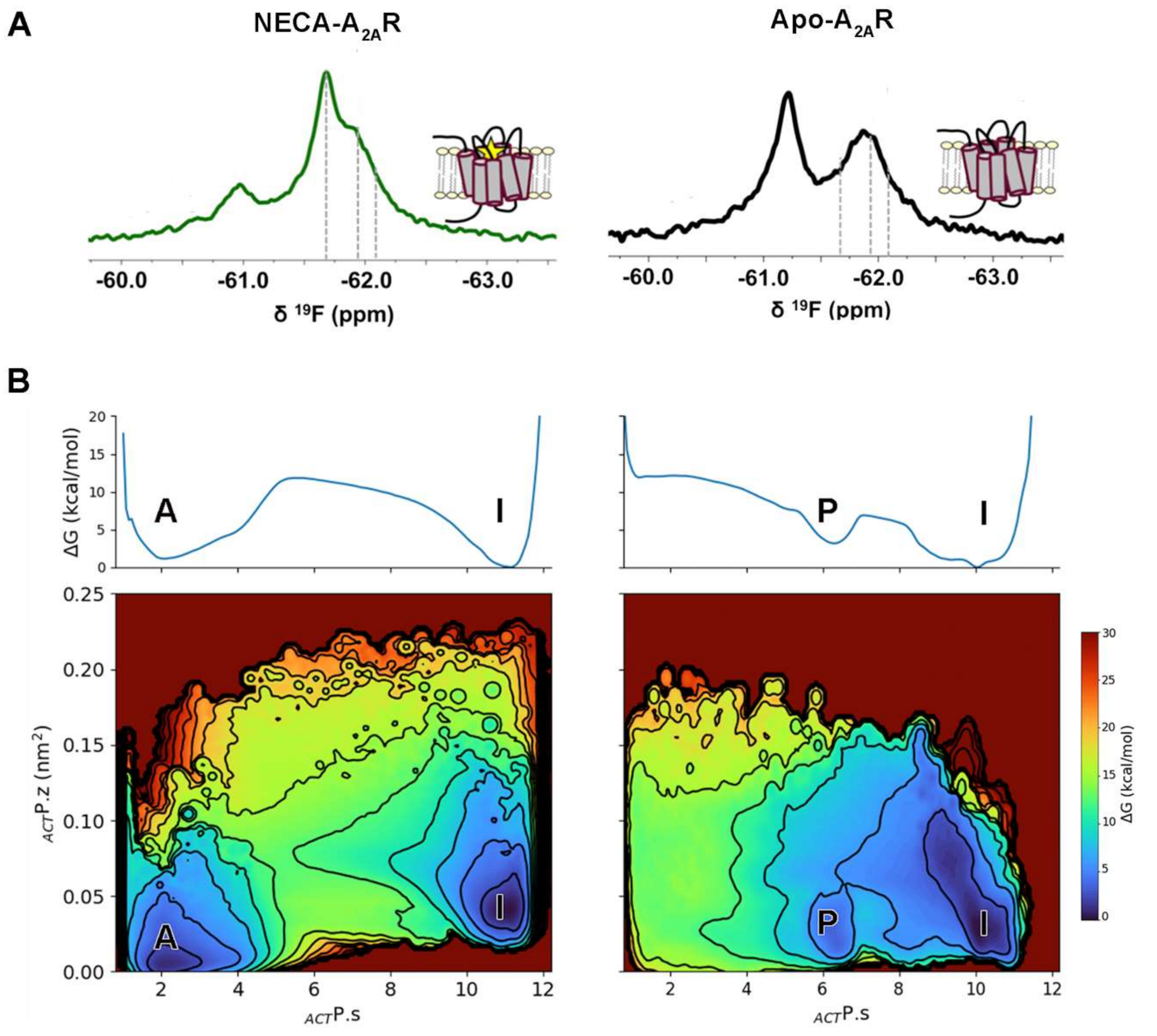

Figure S23. Comparison between ^{19}F-NMR spectra and free energy surfaces of A_{2A}R. A) ^{19}F NMR spectra of A_{2A}R-V229C in *apo* conditions and in presence of NECA reported by Prosser and coworkers (*Cell,* **2021**, 184, 1884–1894). B) Activation free energy surfaces of the *NECA-bound*, *apo*, and *ZMA-bound* A2AR as a function of the $_{ACT}$P.s and $_{ACT}$P.z collective variables computed by means of PCV-MetaD.
