# Supplementary material for: Minute-timescale free-energy calculations reveal a pseudo-active state in the adenosine A2A receptor activation mechanism
Source: Chem. 2024 Dec 12;10(12):3678–98. doi: 10.1016/j.chempr.2024.08.004 (PMC11965979; doi:10.1016/j.chempr.2024.08.004)
Supplement: Document S2. Article plus supplemental information [file mmc3.pdf]

Chem

Article

Minute-timescale free-energy calculations reveal a pseudo-active state in the adenosine A_{2A} receptor activation mechanism

Graphical abstract

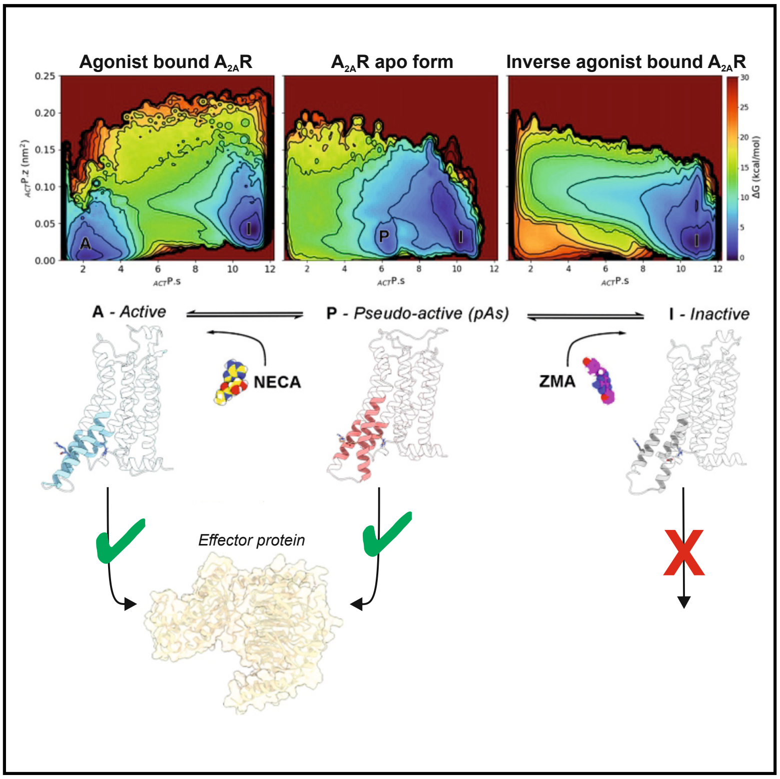

Highlights

- Elucidation of the adenosine A_{2A} receptor ($A_{2A}R$) activation mechanism
- Characterization of the ligands' intrinsic pharmacological activity
- Identification of a pseudo-active state (pAs) of the $A_{2A}R$ apo form

Authors

Vincenzo Maria D'Amore, Paolo Conflitti, Luciana Marinelli, Vittorio Limongelli

Correspondence

vittoriolimongelli@gmail.com

In brief

D'Amore et al. uncover the activation mechanism of the adenosine A_{2A} G protein-coupled receptor in its basal apo form and under different ligand-bound conditions using minute-timescale free-energy calculations. They characterize the ligands' intrinsic activity and discover a receptor pseudo-active state (pAs) that enables coupling with G protein, providing a structural basis for understanding receptor function and signaling.

D'Amore et al., 2024, Chem *10*, 3678–3698
December 12, 2024 © 2024 The Author(s).
Published by Elsevier Inc.
https://doi.org/10.1016/j.chempr.2024.08.004

CellPress

CellPress
OPEN ACCESS

Chem

Article

Minute-timescale free-energy calculations reveal a pseudo-active state in the adenosine A_{2A} receptor activation mechanism

Vincenzo Maria D'Amore,[1] Paolo Conflitti,[2] Luciana Marinelli,[1] and Vittorio Limongelli[2,3,*]

[1]Dipartimento di Farmacia, Università degli Studi di Napoli "Federico II", Via D. Montesano 49, 80131 Naples, Italy

[2]Euler Institute, Faculty of Biomedical Sciences, Università della Svizzera italiana (USI), via G. Buffi 13, CH-6900 Lugano, Switzerland

[3]Lead contact

*Correspondence: vittoriolimongelli@gmail.com

https://doi.org/10.1016/j.chempr.2024.08.004

THE BIGGER PICTURE G protein-coupled receptors (GPCRs) are membrane proteins targeted by over one-third of marketed drugs. Once activated, GPCRs undergo large conformational changes to interact with various intracellular effector proteins. Understanding their activation mechanism is crucial for developing precise and effective drugs. Our study reveals the conformational landscape of the adenosine A_{2A} receptor (A_{2A}R) activation mechanism in its basal apo form and under different ligand-bound conditions using advanced calculations. We characterized the ligands' intrinsic activity and the receptor's lowest-energy functional states, identifying a pseudo-active state (pAs). This state enables A_{2A}R to couple with Gs protein, offering a structural basis for understanding receptor function and signaling. Our versatile simulation method is applicable to any GPCR, potentially enabling breakthroughs in drug discovery by identifying new targets for tailored modulation of drug pharmacology.

SUMMARY

G protein-coupled receptors (GPCRs) are membrane proteins targeted by over one-third of marketed drugs. Understanding their activation mechanism is essential for precise regulation of drug pharmacological response. In this work, we elucidate the conformational landscape of the adenosine A_{2A} receptor (A_{2A}R) activation mechanism in its basal apo form and under different ligand-bound conditions through minute-timescale free-energy calculations. We identified a pseudo-active state (pAs) of the A_{2A}R apo form, stabilized by specific "*microswitch*" residues, including a salt bridge established between the conserved residues $R^{5.66}$ and $E^{6.30}$. The pAs enables A_{2A}R to couple with Gs protein upon rearrangement of the intracellular end of transmembrane helix 6, providing unprecedented structural insights into receptor function and signaling dynamics. Our simulation protocol is versatile and can be adapted to study the activation of any GPCRs, potentially making it a valuable tool for drug design and "*biased signaling*" studies.

INTRODUCTION

G protein-coupled receptors (GPCRs) are prominent pharmacological targets, representing 4% of the protein-coding genome and being targeted by almost 34% of the currently marketed drugs.[1,2] In response to extracellular stimuli like hormones, neurotransmitters, and odorants, GPCRs regulate a plethora of biological functions, including vision, inflammation, and sensory perception.[1] They show a conserved structural architecture arranged in seven transmembrane helices (TMs), connected through three extracellular and three intracellular loops (ECLs and ICLs, respectively).[3] The GPCR barrel-like tertiary structure can be depicted in three main sections (Figure 1): (1) the orthosteric ligand binding site (OBS) at the extracellular region, (2) the connector, and (3) the intracellular binding site (IBS) where binding of effector proteins (aka transducers) occurs.[8] The operating system is a ternary complex where the GPCR is bound to a ligand and at the same time to a transducer that triggers the signal cascade inside the cell.[9] The GPCRs are endowed with intrinsic functional dynamics, and upon agonist binding, the receptor undergoes large-scale conformational changes passing from the inactive to the active state. For instance, in rhodopsin-family class A GPCRs, the two terminal states—i.e., active and inactive—differ in an outward/inward motion of the intracellular part of TM6 (~12–14 Å), accompanied by a rotation of the same helix around its axis (~40°–50°) and slight shifts of

 © 2024 The Author(s). Published by Elsevier Inc.
This is an open access article under the CC BY license (http://creativecommons.org/licenses/by/4.0/).

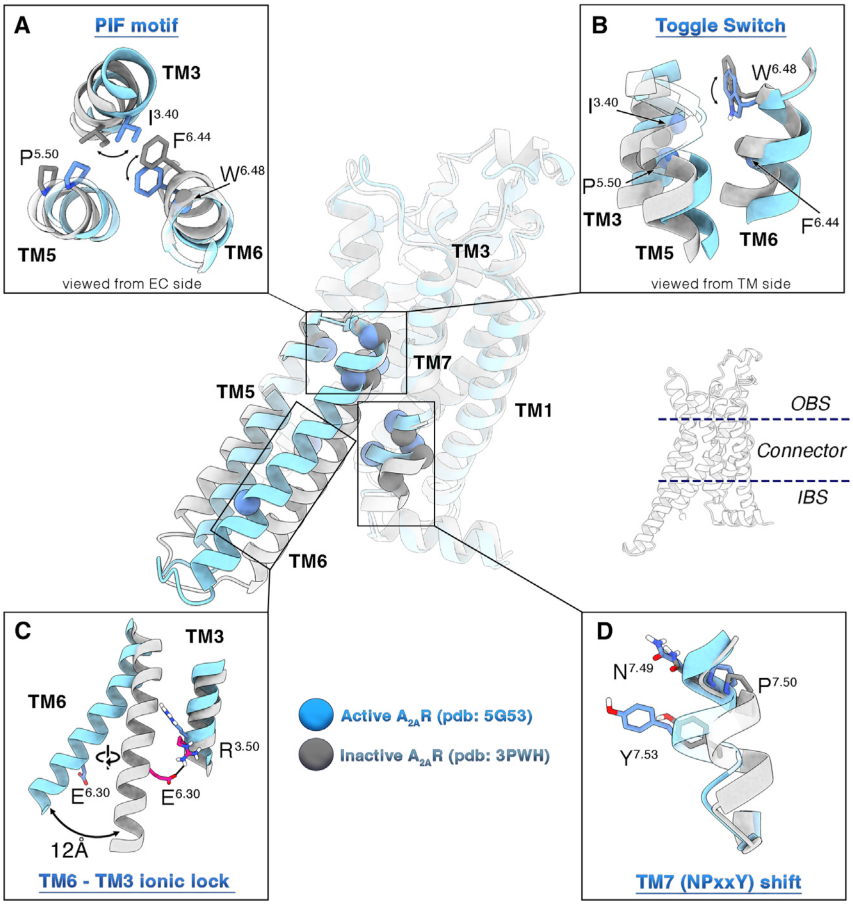

Figure 1. Main conformational changes occurring during class A GPCR activation

Active and inactive conformations of the $A_{2A}R$ are shown as cyan and silver cartoon, respectively. The four fundamental class A microswitches are highlighted as insets: the PIF motif (connector region, A), the $W^{6.48}$ rotameric state toggle switch (OBS/connector region, B), the TM6-TM3 inactivating ionic lock (IIL; IBS region, C), and the NPxxY inward/outward shift (IBS region, D). In some experimental inactive structures of $A_{2A}R$, the TM6-TM3 ionic lock (IIL) is broken, suggesting the existence of two inactive states with the ionic lock formed and broken.[4–7] The residues mainly involved in these transitions are shown as spheres (representing the Cα atoms) onto the central representations of active and inactive receptors, and they are shown as sticks in the insets. Non-polar hydrogens are omitted for the sake of clarity.

the intracellular end of TM5 and TM7 (Figure 1).[9–11] In doing so, the GPCR IBS opens up, promoting the interaction with the transducer. On the other hand, minor differences are found with respect to the OBS by comparing active and inactive structures (in the range of 1.5–2 Å for backbone atoms).[12,13] Nonetheless, the receptor (de)activation appears to be regulated by fine-grained allosteric communication between the two regions. Indeed, the binding of an agonist to the OBS promotes the recruitment of an intracellular transducer at the IBS; at the same time, the binary transducer-GPCR complex has higher affinity for agonist than the sole receptor.[14–16] In addition, GPCRs can trigger a wide range of cellular pathways[14,17–19] by coupling with diverse effector proteins such as G proteins,[17,19] GPCR kinases (GRKs),[20] proto-oncogene c-Src,[21] and arrestins.[22,23] In this scenario, a ligand might induce specific receptor conformations competent for binding to a certain effector, thus selectively activating downstream cell signaling.[24–29] This phenomenon, known as "*biased signaling*,"[24,26] brought to the fore new important implications for the pharmaceutical and clinical application of GPCR-targeting drugs.[24,27,30] For instance, developing molecules capable of selectively activating or inhibiting a specific signaling cascade can yield a more targeted modulation of cell function with consequently reduced adverse effects,[31,32] as recently reported for antidepressant drugs targeting the serotonin 5-HT_{2A} receptor[33] and analgesics targeting adenosine A_1[34] and μ opioid receptors.[35] Nevertheless, the rational design of "*biased*" ligands is hampered by the lack of structural information on all the functional conformations assumed by the GPCRs along their activation process. In fact, although the so-called "*resolution revolution*"[36] in structural biology is constantly advancing the molecular understanding of the GPCRs fundamental states, other important aspects of their functional mechanism remain elusive, including the activation dynamics and the possible presence of receptor metastable, intermediate states. Atomistic simulations based on molecular dynamics (MD) techniques have demonstrated ability in detecting dynamic properties of the receptor, including the interaction with ligands and effectors.[13,35,37–55] Particularly, enhanced sampling techniques like metadynamics (MetaD) or alternative methodologies like Gaussian accelerated MD have been used to study the allosteric ligand effects on GPCR conformational dynamics[56] as well as the protein-protein binding interaction during GPCR dimerization and between the adenosine receptors and G proteins.[57–59]

In the present work, we elucidate the activation mechanism of the adenosine A_{2A} receptor ($A_{2A}R$), a class A GPCR, providing structural and energetic information on all the functional conformations assumed by the receptor in different ligated conditions. In particular, we performed extensive MD simulations and enhanced sampling calculations that allowed disclosing the free-energy landscapes of the (de)activation process of $A_{2A}R$ (1) in the *apo* form; (2) in complex with the full agonist (2S,3S,4R,5R)-5-(6-amino-9H-purin-9-yl)-N-ethyl-3,4-dihydroxyoxolane-2-carboxamide (NECA); and (3) in complex with the compound 4-(2-{[7-Amino-2-(2-furyl)[1,2,4]triazolo[1,5-a][1,3,5]triazin-5-yl]amino}ethyl)phenol (ZM241385, also known as "*ZMA*" or "*ZM*"), whose intrinsic activity (neutral antagonist vs. inverse agonist) is still debated.[4–6,60–63] In each system, we have identified at atomistic resolution the lowest energy—hence

most probable—states assumed by $A_{2A}R$, rationalizing the diverse pharmacological activity of the investigated ligands by elucidating how they affect the receptor activation free-energy landscape. In particular, the binding of ZMA locks the receptor in the inactive state, whereas the agonist NECA predisposes $A_{2A}R$ for the active conformation competent for G protein binding, with the definitive activation of the receptor—and stabilization of the active form—occurring only after the G protein coupling. Importantly, we disclose two structures of $A_{2A}R$ in the *apo* form that were not previously resolved and connect them to the nuclear magnetic resonance (NMR) and fluorescence data reported for this receptor.[5–7,15,63–66] One is similar to the inactive state experimentally found in the presence of inverse agonist ligands. The other one corresponds to a novel receptor conformation that we named the "*pseudo-active state*" (pAs). This structure is characterized by a distinctive arrangement of the connector region and in particular TM6, with a state-specific orientation of the "*activation microswitches*" amino acids, such as $W^{6.48}$ (Ballesteros-Weinstein numbering used)[67] ("*toggle switch*"), the $E^{6.30}$/$DRY^{3.49-51}$, the $P^{5.50}I^{3.40}F^{6.44}$, and the $NPxxY^{7.49-53}$ motifs (Figure 1).[51,68–70] Among these, the class A conserved residue $E^{6.30}$ plays a leading role during $A_{2A}R$ dynamics, determining the TM6 rotation necessary for receptor activation. In particular, $E^{6.30}$ works as the key "*activation*" switch by interacting with $R^{3.50}$ in the $A_{2A}R$ inactive state—forming the characteristic "*inactivating ionic lock*" (IIL) found in many GPCRs[68,69]—whereas in the active state, it engages a salt bridge with $R^{5.66}$. The phylogenetic conservation of $R^{5.66}$ and $E^{6.30}$ in class A GPCRs prompted us to refer to the $E^{6.30}$/$R^{5.66}$ interaction as the receptor "*activating ionic lock*" (AIL). Interestingly, in the newly identified pAs, $A_{2A}R$ is able to couple with the Gs protein upon minor rearrangement at the TM6 intracellular end, as well as with β-arrestin 1, enlightening novel possible routes for receptor signaling studies.

Our simulation protocol is generalizable and can be applied to study the activation of any GPCRs, resulting in a valuable tool for biased signaling studies. An explanatory movie of the $A_{2A}R$ activation mechanism is available as Video S1 and at https://youtu.be/TbXi3KjIWFo.

The Protein Data Bank (PDB) structure of the $A_{2A}R$ pAs is reported in the supplemental information and at www.pdbdb.com, providing an unprecedented structural basis for the design of $A_{2A}R$ ligands with therapeutic potential for cancer, inflammatory, cardiovascular, and Parkinson's and Alzheimer's diseases.[71–76]

RESULTS

Effect of ligand binding and G protein recruitment on $A_{2A}R$ conformational dynamics

The $A_{2A}R$ can assume a large number of conformations ranging from the active state,[77,78] bound to agonist and G protein, to the inactive state, typically bound by antagonist or stabilized by inverse agonist binding.[4,79] In order to investigate how the diverse ligands and G protein influences the receptor dynamics, we first performed a series of extensive all-atom MD simulations on differently ligated forms of $A_{2A}R$. In particular, starting from the experimental structures of the active and inactive $A_{2A}R$,[77,80] we prepared eight distinct simulation systems in which the GPCR is coupled with pharmacologically diverse ligands—the agonist NECA and the inverse agonist ZMA—and the mini-Gs protein heterotrimer in all possible combinations (Table 1). The $A_{2A}R$ was embedded in a mixed 1-palmitoyl-2-oleoylphosphatidylcholine (POPC)-cholesterol (7:3 ratio) membrane environment, and each system was simulated in explicit solvent for 5 μs, resulting in a total simulation time of ~40 μs.

Our simulations allowed for elucidating the stabilizing effect of orthosteric ligands and G protein on their binding sites, the OBS and the IBS, respectively. In the OBS, higher root-mean-square deviation (RMSD) fluctuations (calculated for the Cα atoms) are generally observed for the apo systems (1.4 ± 0.2 Å, 1.2 ± 0.3 Å, and 1.4 ± 0.2 Å for "*FApo,*" "*FApoG,*" and "*INapo,*" respectively) compared with the holo ones (0.9 ± 0.1 Å, 0.9 ± 0.1 Å, and 1.1 ± 0.2 Å for "*FAN,*" "*FANG,*" and "*INzma,*" respectively) (Figure S1). This phenomenon can be ascribed to the drug-protein interactions engaged by NECA and ZMA, which stabilize the OBS side-chain conformations and consequently reduce the backbone fluctuation. Only the holo Gs-uncoupled systems "*INeca*" and "*ACzma*" exhibit relatively high RMSD values of the OBS (1.4 ± 0.2 Å and 1.4 ± 0.2 Å, respectively). This can be attributed to induced fit effects of NECA and ZMA on the inactive and active form of $A_{2A}R$, respectively, which are not the receptor's natural conformations for these ligands. In fact, no experimental structures of inactive $A_{2A}R$ bound with the agonist NECA or active $A_{2A}R$ bound with the inverse agonist ZMA have been reported. Consequently, the initial states for the *INeca* and *ACzma* simulations were generated by manually docking the two compounds into the inactive and active experimental structures of $A_{2A}R$, respectively (see Experimental procedures for details). These simulations are designed to investigate the effect of the ligands on the receptor conformational dynamics and whether NECA and ZMA could induce transitions toward the active and inactive states, respectively, in the absence of G protein.

Similarly to the ligands at the OBS, Gs stabilizes the IBS. In fact, the mini-Gs heterotrimer engages strong and specific interactions with IBS residues, locking $A_{2A}R$ in its active state, as shown by the RMSD comparison (Figure S2) between the two coupled systems (1.7 ± 0.4 Å and 1.7 ± 0.4 Å for *FANG* and *FApoG*, respectively) and the corresponding uncoupled ones (1.9 ± 0.5 Å and 3.1 ± 0.8 Å for *FAN* and *FApo*, respectively).

Interestingly, allosteric communications between the OBS and the IBS have been found looking at the motion of the intracellular receptor region in the holo Gs-uncoupled systems (1.9 ± 0.5 Å and 1.8 ± 0.2 Å for *FAN* and *INzma*, respectively). In fact, in such cases, the RMSD of the IBS is lower in terms of both values and fluctuations, compared with that of the two apo Gs-uncoupled trajectories (3.1 ± 0.8 Å and 2.2 ± 0.4 Å for *FApo* and *INapo*, respectively). This result suggests that the $A_{2A}R$'s intracellular portion can be stabilized by the binding of a specific ligand to the OBS, in addition to the stability given by the binding of transducers at the IBS. In this perspective, we investigated the presence of correlated $A_{2A}R$ inter-helical motions in the different simulated systems by computing a Pearson coefficient (PC) matrix (Figures 2A and S3). Interestingly, this analysis confirmed that the overall receptor dynamics is strongly reduced when

Table 1. Summary of the simulated systems

	Code name	Starting conformation	Ligand	G protein coupling
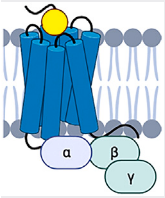	FANG	active	NECA (agonist)	yes
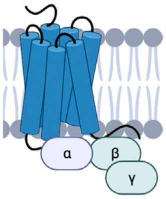	FApoG	active	none (*apo form*)	yes
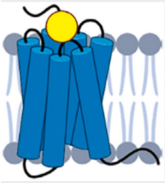	FAN	active	NECA (agonist)	no
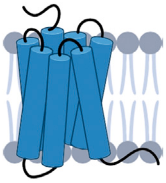	FApo	active	none (*apo form*)	no
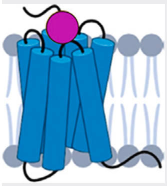	ACzma	active	ZMA (inverse agonist)	no
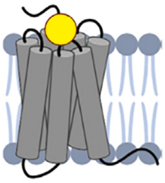	INeca	inactive	NECA (agonist)	no
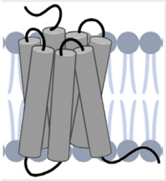	INapo	inactive	none (*apo form*)	no
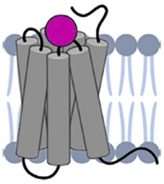	INzma	inactive	ZMA (inverse agonist)	no

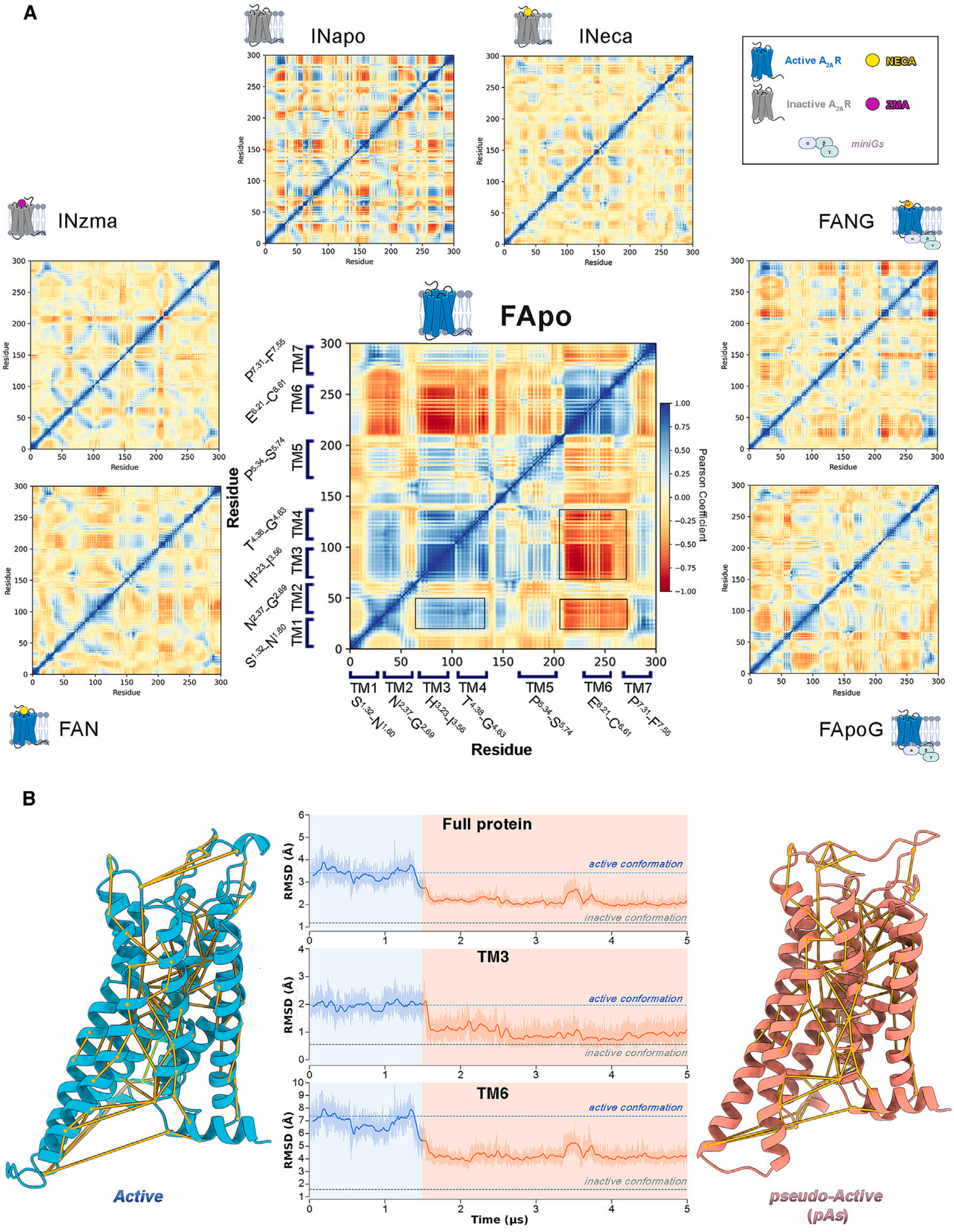

Figure 2. Active to pseudo-active transition

(A) Pearson coefficient matrices computed for the protein Cα atoms over the seven simulated systems.

(B) RMSD plots computed with respect to the inactive $A_{2A}R$ conformation for the entire protein and for both the TM3 and TM6 helices (Cα atoms) over the *FApo* trajectory. The tri-dimensional structures of the starting full active conformation and of the newly identified pseudo-active state (pAs) are shown as cyan and coral cartoon, respectively. The most relevant protein structure network (PSN) metapath of the transition observed in the *FApo* trajectory is displayed onto the active-state (left) and the pAs (right) structures as yellow links.

either the orthosteric ligand or G protein—or both—are bound to the GPCR. Indeed, looking at the PC maps (Figures 2A and S3) few spots at positive (blue spots, PC > 0.5) or negative (red spots, PC < 0.5) correlation values are found in all the holo/coupled systems, whereas significant inter-helical communication areas were observed in the apo-uncoupled systems *INapo* and *FApo*. Particularly, among all the simulated systems, *FApo* shows the largest conformational changes with a tight coupling (Figures 2A and S3) between the fluctuations of TM6 and the intracellular parts of TM1-TM2 and TM3-TM4, which in turn are mutually anti-correlated. These data indicate a rearrangement of the receptor, especially at intracellular level, in line with the RMSD values computed for the IBS (Figure S2), the receptor's TMs, and the connector region (Figures S4 and S5, respectively).

Indeed, within ~1.6 μs, the *FApo* $A_{2A}R$ undergoes a major conformational rearrangement of TM3 and TM6, leaving its starting active state and reaching a conformation intermediate between the active and inactive states (Figure 2B). Such large-scale motion is characterized by two main events (see Video S1). In the first one, the two helices move in opposite directions along the axis perpendicular to the membrane plane (*z*), with TM3 shifting downward (intracellularly) and TM6 upward (extracellularly) by ~3 Å. In the second one, the intracellular segment of TM6 approaches the center of the TM bundle, assuming a state-specific tilted conformation (Figure 2). This represents the final state of the receptor that is stable for the rest of the simulation time, longer than 3.4 μs (Figure S6). The newly identified $A_{2A}R$ state, i.e. the pAs, has never been characterized before, and its remarkable structural and energetic stability prompted us to deeply analyze this receptor conformation in the following section. The PDB structure of the $A_{2A}R$ pAs is reported in the supplemental information and at www.pdbdb.com, whereas an explanatory movie of the $A_{2A}R$ activation mechanism is available as Video S1 and at https://youtu.be/TbXi3KjIWFo.

Structure of $A_{2A}R$ pAs

The tri-dimensional structure of the newly identified $A_{2A}R$ pAs, corresponding to the most sampled receptor conformation in the pAs state, is rather different from the reported inactive (ZMA-bound, PDB: 3PWH[4]) and active (NECA and G protein-bound, PDB: 5G53[77]) states of $A_{2A}R$, with RMSD values computed for the backbone Cα atoms of 2.52 Å vs. 3.28 Å, respectively. A closer inspection of the structure reveals the key residues that stabilize this receptor conformation as transition intermediate between the active and inactive forms. Such residues were identified by analyzing the $A_{2A}R$ conformations collected during the *FApo* MD simulation through a protein structure network (PSN) model that is able to assess time-related residue-residue interactions (Figures 2B and S3 and Experimental procedures for details). Special attention was dedicated to the analysis of residues known as activation microswitches in class A GPCRs.[4,51,68–70] For the sake of clarity, the following discussion is organized treating separately the three main structural components of GPCRs: the OBS, the connector, and the IBS regions.

Orthosteric Binding Site (OBS)

In this region, the pAs is more similar to the inactive conformation than the active one. Proof of that is the lower RMSD values computed for the OBS residues in the pAs with respect to those of the inactive and the active states, 0.9 and 1.2 Å, respectively. This evidence is further confirmed by analyzing the conformations of the OBS residues known to be involved in receptor activation, such as $V84^{3.32}$, $T88^{3.36}$, $S277^{7.42}$ and $W246^{6.48}$.[77,78,81–83] In fact, such amino acids occupy a position very similar to that observed in the inactive receptor (Figure 3A′), while major differences occur with respect to the active conformation (Figure 3A). In more detail, the side chains of $T88^{3.36}$ and $S277^{7.42}$ in the pAs are oriented outward in relation to the binding site, if compared with their position in the experimental active $A_{2A}R$ structures (Figure 3A). In fact, in the latter structures, these two residues engage in polar interactions with the agonists' ribose ring, which are instead missing when the receptor is either in its apo form or bound to antagonists/inverse agonists.[77,78,82–84] Also, the rotameric state of $V84^{3.32}$ and $W246^{6.48}$ (toggle switch) is more similar to the inactive conformation than the active one in which the agonist's ribose ring shifts $V84^{3.32}$ and $W246^{6.48}$ toward an outward conformation and a downward conformation, respectively (Figures 3A and 3B).

Connector region

At variance with the OBS, the conformation assumed by the pAs connector region is state specific, dissimilar from both the active and the inactive states—RMSD values of 2.3 and 2.4 Å, respectively—and is characterized by a distinctive orientation of the $P^{5.50}I^{3.40}F^{6.44}$ motif. Comparing the pAs to the active $A_{2A}R$, TM6 shows an upward movement along the *z* axis and a counterclockwise rotation, which orient the $F242^{6.44}$ side chain outward with respect to the active and inactive structures (Figures 3B and 3B′). On the other hand, TM3 is shifted downward, with $I92^{3.40}$ assuming its inactive state position (Figure 3B′). This is an unprecedented finding in the context of the $A_{2A}R$ (de)activation mechanism. In fact, in all the structural studies reported so far, TM6 was found to move inward and rotate clockwise when passing from the active to the inactive $A_{2A}R$.[4,77,78,82–84] Instead, in the pAs, TM6 moves in the opposite direction and is rotated counterclockwise to facilitate the vertical motion of TM3 and the onset of the deactivation process.

Intracellular Binding Site (IBS)

In the pAs, TM6 is closer to the active state and further from the inactive state, compared with the experimental intermediate-active state (PDB: 2YDO) (Figure S7). The intracellular portion of the receptor is also characterized by a bending of TM6. Specifically, the cytoplasmic end of TM6 is bent toward the center of the TM bundle (Figures 2 and 3C′), reducing the volume of the IBS to values comparable to that of the inactive $A_{2A}R$ X-ray structure (Figure S8). However, two major differences arise by comparing the pAs and the inactive-state IBSs. First, the middle-lower portion of TM6 (residues $229^{6.31}$–$237^{6.39}$) is slightly shifted outward (Figure 3C′). This might be due to the steric hindrance of the $NPxxY^{7.49\text{-}53}$ motif at TM7 in the pAs. In fact, such a motif assumes a position very similar to that of the active $A_{2A}R$ (Figure 3D; Table S1), where $Y288^{7.53}$ interacts with $Y197^{5.58}$ on TM5 through a direct or water-mediated H-bond, which is known to stabilize the receptor's active state.[70] The second relevant feature is that TM6 is rotated about 40°–50° counterclockwise with respect to the inactive state (Figure 3C′). This conformation is stabilized by salt bridge interaction between $R205^{5.66}$ of TM5

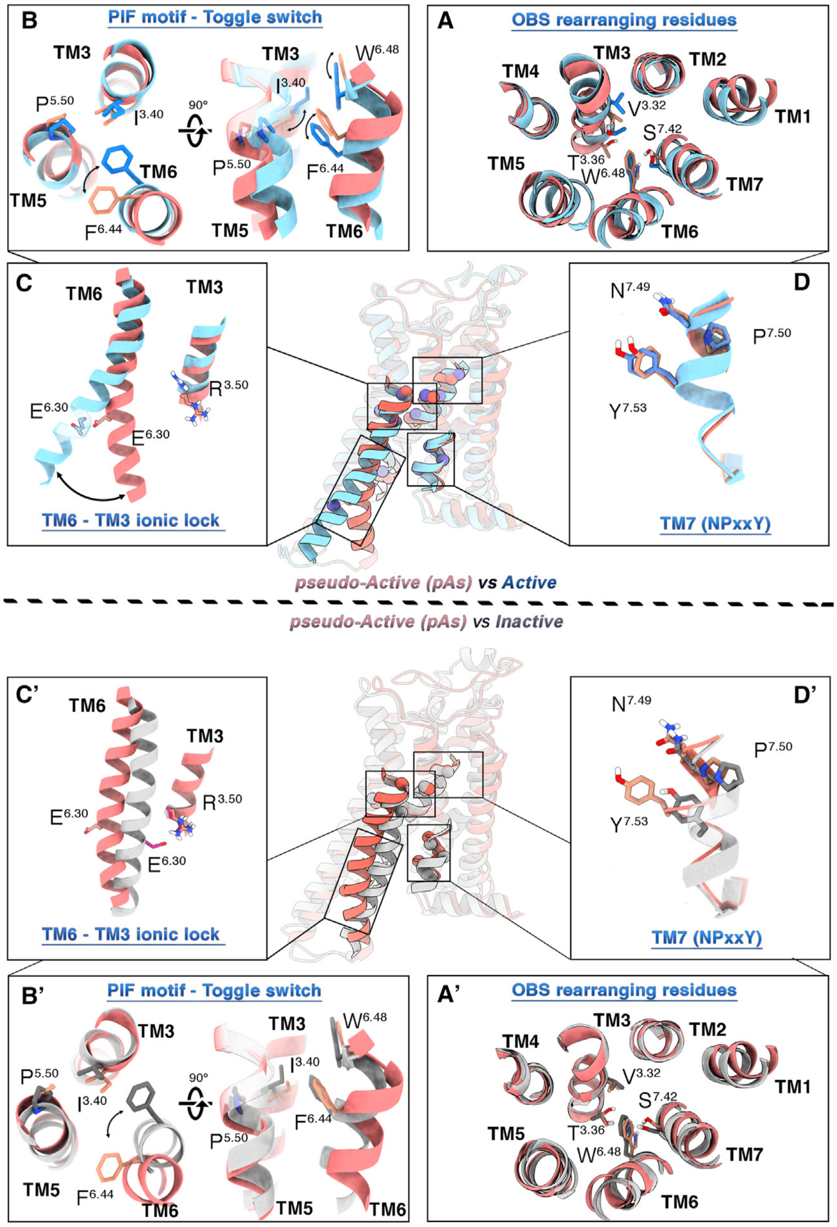

Figure 3. Atomistic details of the newly discovered $A_{2A}R$ pAs

The conformations of the main activation microswitch residues belonging to the $A_{2A}R$ pAs (salmon) were compared with both the active (PDB: 5G53, color: light blue, upper panel) and the inactive (PDB: 3PWH, color: silver, lower panel) $A_{2A}R$ states.

and E228$^{6.30}$ of TM6 (Figure 4A). Recently, Wang et al. have highlighted the role of cation-π interactions involving R291$^{7.56}$ and R293$^{8.48}$ with H230$^{6.32}$ during receptor activation.[7] Our MD simulations confirm these interactions in the inactive state, aligning with findings by Wang et al. (Figure S9). In the active state, both cation-π interactions are weakened or lost, whereas in the pAs, R291$^{7.56}$ and R293$^{8.48}$ are slightly closer to H230$^{6.32}$, compared with the active state, suggesting an intermediate conformation.

The results of our simulations show that the salt bridge interaction between R205$^{5.66}$ and E228$^{6.30}$ is very stable in all the $A_{2A}R$ structures with TM6 in an active-like rotameric state (frequency of occurrence > 95%; Figure 4B), whereas it is lost in all the $A_{2A}R$ inactive conformations (Figure 4B). In the latter, E228$^{6.30}$ is oriented toward the inner part of the IBS and interacts with R102$^{3.50}$ of the DRY motif, forming the IIL. Interestingly, R205$^{5.66}$ and E228$^{6.30}$ are highly conserved among the class A GPCRs (37% and 35%, respectively; Figure 4C), prompting us to propose the E228$^{6.30}$-R205$^{5.66}$ salt bridge as the AIL, *alter ego* of the IIL, whose loss facilitates the clockwise rotation of TM6 and in turn the deactivation process. Notably, a similar interaction was found in the active state structures of the rhodopsin receptor—where R$^{5.66}$ is mutated to K$^{5.66}$ (PDBs: 3CAP and 3DQB)[38,85,86]—and in the A_3 adenosine receptor through *in silico* studies.[87] Additionally, we examined the formation of the salt bridge between TM5 and TM6 in the experimental active structures of $A_{2A}R$ (PDBs: 5G53,[77] and 6GDG[78]) and the recently released active states of the A_1 receptor (A_1R) and A_3 receptor (A_3R) (PDBs: 7LD4,[88] and 8X17[89]). In all these cases, the ICL3 connecting TM5 and TM6, near E$^{6.30}$, was unresolved. Consequently, we reconstructed this loop in all systems and also the E$^{6.30}$ side chain for $A_{2A}R$, and we then conducted MD simulations on the complete $A_{2A}R$, A_1R, and A_3R structures embedded in the POPC/CHL (7:3) bilayer. For $A_{2A}R$, we performed two independent simulations using the experimental structures with PDBs: 5G53[77] and 6GDG[78] as starting states. As shown in Figure S10, in all systems, R$^{5.66}$ on TM5 and E$^{6.30}$ on TM6 rapidly interact to form a stable salt bridge that persists throughout the simulation. These findings underscore the importance of the TM5-TM6 salt bridge in stabilizing the active state of $A_{2A}R$ and other GPCRs.

The role of the new pAs, along with the transitioning of $A_{2A}R$ from the active to the inactive state, was further investigated by analyzing the MD simulations of the apo G protein-uncoupled $A_{2A}R$ systems. Our results show that within the MD microsecond timescale, the pAs can be reached from the active state (*FApo* system) but not from the inactive one (*INapo* system), which is instead stable throughout the simulation (Figure S4). Furthermore, the pAs is a very stable, long-lasting state with a residence time longer than 3 μs (Figure S6). This finding indicates that the pAs is a metastable, intermediate conformation between the active and inactive ones, which are separated by a relatively large energy barrier that is unlikely to be crossed within a

CellPress
OPEN ACCESS

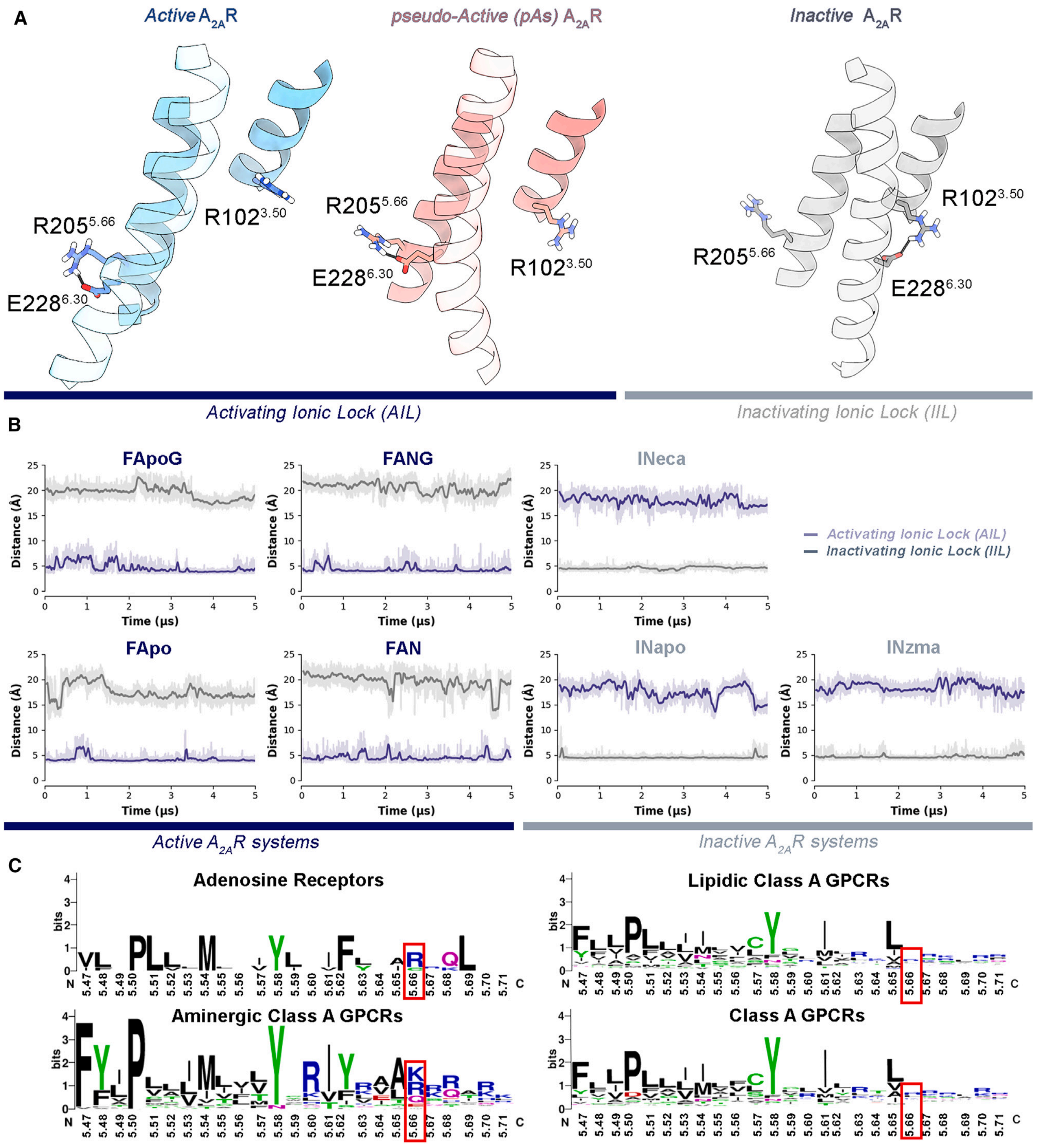

Figure 4. Activating and inactivating ionic locks

(A) 3D representation of the "*activating ionic lock*" (AIL) and "*inactivating ionic lock*" (IIL) in the $A_{2A}R$'s active, pseudo-active, and inactive states. The helices TM3, TM4, and TM6 are depicted as cartoons, while residues E228$^{6.30}$, R205$^{5.66}$, and R102$^{3.50}$ are highlighted as sticks.

(B) Plots of the AIL (distance between the Cγ atom of E228$^{6.30}$ and the Cζ atom of R205$^{5.66}$) and IIL (distance between the Cγ atom of E228$^{6.30}$ and the Cζ atom of R102$^{3.50}$) along the seven MD systems.

(C) LOGOs analysis of residues 5.47–5.71 of TM5. The conservation percentages for basic amino acids at position 5.66 in the four investigated subsets (adenosine, aminergic, lipidic, and the entire class A) of class A GPCRs are, respectively, 75%, 68%, 38%, and 37%.

microsecond (simulation) timescale. In order to observe the transition between the active and inactive states, it is necessary to accelerate the sampling and overcome the timescale limitation of standard MD simulations. This is possible by employing enhanced sampling calculations based on well-tempered MetaD (WT-MetaD)[90,91] combined with path collective variables (PCVs) (see Experimental procedures for details).[92] Particularly, PCV is a dimensionality reduction approach suitable to describe large-scale protein motion, taking into account multiple degrees of freedom of a system for which the terminal states are known, as in the $A_{2A}R$ case. The process under investigation is thus accelerated by applying a bias potential along the PCV that is defined as a path comprising a sequence of intermediate frames connecting the two terminal systems' conformations ($A_{2A}R$ active and inactive states in our case). We note that during the PCV-WT-MetaD calculations, the receptor can explore conformations even different from the original path, thus making the results independent from the choice of the original path. PCV-MetaD calculations have been successfully employed by us and other groups to study large-scale and long-timescale conformational changes in different protein systems.[93–99] In the case of $A_{2A}R$, the path is defined considering the pAs as an intermediate state between the terminal active and inactive states and including the residues involved in the conformational transitions observed during the multi-microsecond MD calculations (see Experimental procedures for details).

Activation free-energy landscape of $A_{2A}R$

The entire (end-to-end) activation and deactivation process of $A_{2A}R$ was investigated by means of PCV-MetaD in three different systems: (1) "*apo*" $A_{2A}R$, (2) "*NECA-bound*" $A_{2A}R$, and (3) "*ZMA-bound*" $A_{2A}R$. In all of them, the sampling was enhanced by adding a bias potential on two PCVs, containing all the inter-residue contacts involved in the conformational receptor transitions observed in the previously discussed MD calculations (see Figure S11 and Experimental procedures for the details). In particular, the first PCV ($_{ACT}$P) is defined as an RMSD matrix describing the geometric distance of the backbone atoms involved in the active-to-inactive receptor transition (see Table S2 and Figure S11A). The second PCV ($_{TM6}$P) is instead defined as a contact map (CMAP) between residues characterizing the rotation of TM6, clockwise from active to inactive (see Table S3 and Figure S11B). The three free-energy calculations reached convergence at different simulation times: 2.4 μs for *apo* $A_{2A}R$, 3.5 μs for *NECA-bound* $A_{2A}R$, and 3.6 μs for *ZMA-bound* $A_{2A}R$, for a total of 9.5 μs of enhanced sampling simulations (Figures S12–S14). Considering the acceleration factor computed during the MetaD calculations, 10^9–10^{10} (see Experimental procedures for detail), we could reasonably estimate that the observed receptor activation and deactivation process occurs on a minute timescale. For each system, at the end of the simulation, we computed the activation free-energy surface (FES) as a function of two CVs, $_{ACT}$P.s and $_{ACT}$P.z (see Experimental procedures for details). The first CV ($_{ACT}$P.s) describes the receptor exploration of the different states forming the transition path from the active to the inactive state, whereas the second CV ($_{ACT}$P.z) defines the distance as MSD of the sampled conformations from the reference path (Figure 5A). As previously introduced, using PCV-MetaD, the systems can explore conformations even distant from the reference path that, in such a case, would have high $_{ACT}$P.z values. Interestingly, in the three systems, all the low energy minima—hence most probable receptor states—have low $_{ACT}$P.z values (< 0.05 nm^2). This result indicates that the reference path employed in WT-MetaD calculations well represents the low energy transition path from active to inactive $A_{2A}R$, leading to a reliable description of the receptor (de)activation process.

Comparing the three FESs in Figure 5, it is possible to assess the effect of ligand binding on the low energy states assumed by the receptor during its active-to-inactive transition. In detail, the *NECA-bound* $A_{2A}R$ is in equilibrium between two energetically comparable states (free-energy difference between minima is 0.8 ± 0.5 kcal/mol). The structure representing the energy minimum A corresponds to the active crystallographic state (RMSD value is 1.4 Å computed for the TM helices with respect to PDB: 5G53[77]), whereas the structure representing the energy minimum I is very similar to the inactive receptor state (RMSD value is 1.6 Å computed for the TM helices with respect to PDB: 3PWH[4]). This finding indicates that upon agonist binding, (1) the receptor can reach the active conformation competent for G protein binding, and (2) the inactive form remains the lowest energy receptor state in the absence of G protein. Similarly to the *NECA-bound* system, the *apo* $A_{2A}R$ has two lowest-energy conformations. While the lowest energy minimum is still represented by the $A_{2A}R$ inactive conformation (state I, RMSD = 1.3 Å), the second energy minimum, 2.9 ± 0.3 kcal/mol higher than I, is not the active state, but state P that corresponds to the pAs structure previously identified by our unbiased MD calculations (RMSD < 1.2 Å). Therefore, the free-energy calculations confirm the presence of the pAs as metastable intermediate between the active and inactive forms of *apo* $A_{2A}R$. On the other hand, the presence of ZMA at the OBS shifts the conformational equilibrium toward the inactive state. In fact, the *ZMA-bound* $A_{2A}R$ has one single low energy minimum (state I), corresponding to the crystallographic pose of the receptor in complex with ZMA. Comparing the FESs in Figure 5, one can see that in *ZMA-bound* $A_{2A}R$, the energy minima of both the active and the pAs are lost in favor of the receptor inactive form. The energetic and structural stability of all the identified minima was further evaluated through microsecond-long unbiased MD simulations (A, P, and I; Figure S15).

In order to investigate in more detail the molecular features characterizing $A_{2A}R$ activation, we computed the FES as a function of $_{ACT}$P.*s* and $_{TM6}$P.*s* (Figure S16). As introduced before, the $_{ACT}$P.*s* CV defines the active-to-inactive receptor transition—specifically the outward/inward motion of helices TM5-6-7—while the second CV ($_{TM6}$P.*s*) describes the *around-the-axis* rotation of TM6 (clockwise or counterclockwise). Looking at the FES and the 1D free-energy profile of the *apo* $A_{2A}R$ system in Figure S16, it is worth noting that the energy barrier separating the minima P and I along the TM6 rotation—$_{TM6}$P.s CV—is 7.4 kcal/mol higher than that along the TM6 translation—$_{ACT}$P.s CV— (14.5 and 7.1 kcal/mol, respectively). A similar result, albeit to a lesser extent, is also obtained for the barrier separating the minima A and I in the *NECA-bound* system (13.2 and 11.5 kcal/mol, respectively). These data indicate that the TM6 rotation represents a slower degree of freedom than the TM6

CellPress
OPEN ACCESS

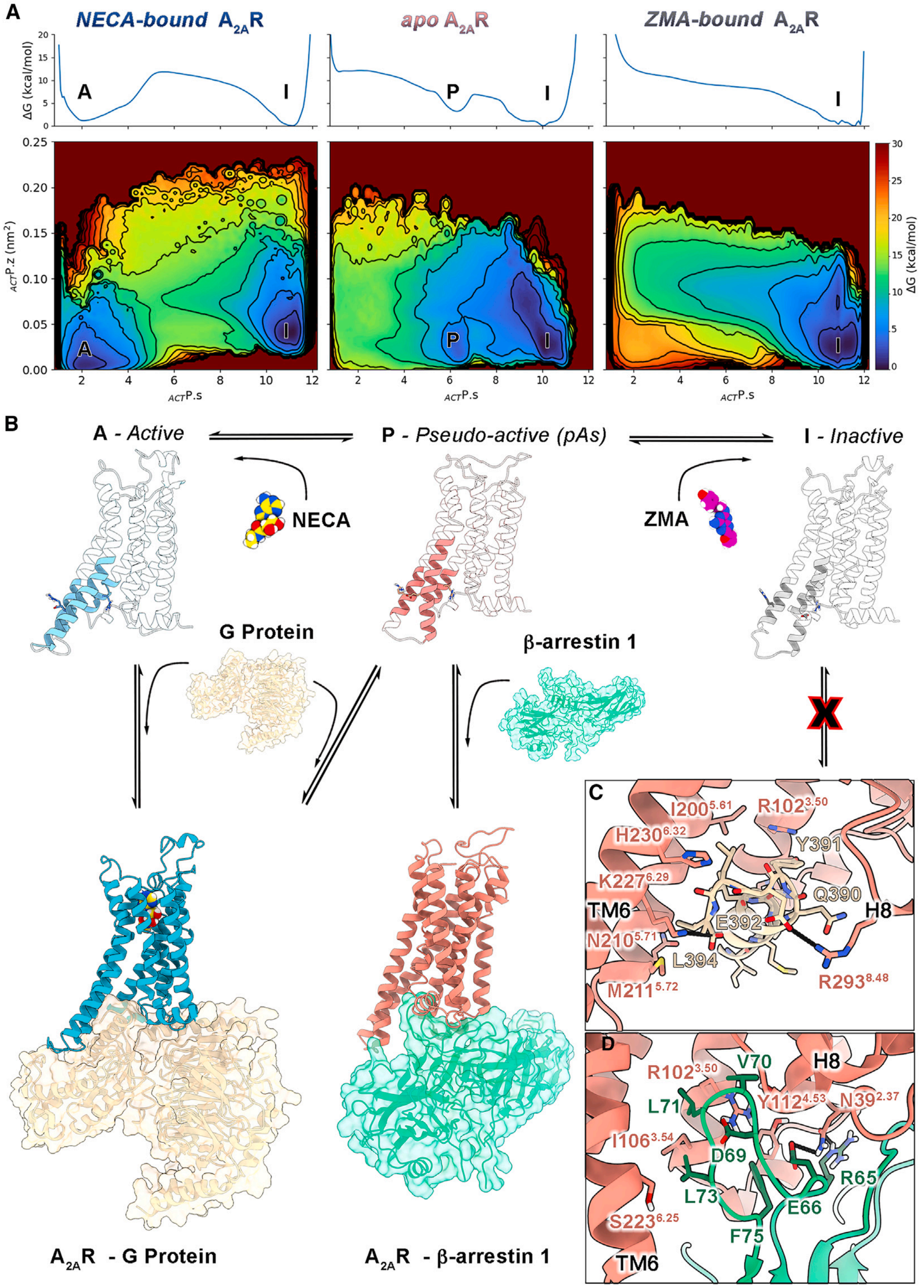

Figure 5. Activation free-energy landscape of $A_{2A}R$

(A) Activation free-energy landscapes of the *NECA-bound*, *apo*, and *ZMA-bound* forms of $A_{2A}R$ as a function of the $_{ACT}P.s$ and $_{ACT}P.z$ collective variables. Isosurfaces are displayed every 3 kcal/mol.

(B) Atomistic representation of the $A_{2A}R$ structures corresponding to the main free-energy minima (A, P, I) and equilibria interconnecting the same conformations along the activation process.

(C) Binding mode of mini Gs (yellow) to the pAs (salmon) upon rearrangement of the TM6 intracellular end.

(D) Binding mode of β-arrestin 1 (green) to the pAs (salmon).

translation and can be considered the rate-determining step of the (de)activation process. Three hubs of microswitch residues rule the receptor motion; they are as follows: (1) the salt bridges engaged by E228$^{6.30}$ with R205$^{5.66}$ in the active form (AIL) and with R102$^{3.50}$ in the inactive one (IIL); (2) the van der Waals interactions established by L235$^{6.37}$, I238$^{6.39}$, and V239$^{6.40}$, with L194$^{5.55}$, L198$^{5.59}$, and F201$^{5.62}$ in the active state and pAs, and with L95$^{3.43}$, I98$^{3.46}$, and I200$^{5.61}$ in the inactive state; and (3) the water-bridged interaction between Y197$^{5.58}$ and Y288$^{7.53}$ in both the active state and pAs. The loss of the latter allows for the outward motion of the NPxxY$^{7.49\text{-}53}$ motif characterizing the receptor deactivation.

Distribution of states in *apo* $A_{2a}R$

Looking at the FES of *apo* $A_{2A}R$ calculated as a function of the $_{ACT}$P.s and $_{TM6}$P.s CVs (Figure 6), five receptor states can be identified. Basin I corresponds to the receptor inactive states. A closer inspection of the structures extracted from this free-energy minimum reveals the co-presence of two equally possible sub-states, I1 and I2, characterized by the TM6-TM3 ionic lock formed and broken (45% vs. 55%). In order to energetically evaluate these two inactive sub-states, we recomputed the free energy as a function of the distance between E$^{6.30}$ and R$^{3.50}$ using a reweighting protocol.[100] The obtained FES clearly shows the two inactive sub-states as separate minima, one where the TM6-TM3 ionic lock is formed and the other where this interaction is broken, I1 and I2, respectively (Figure 6). This finding is in agreement with the existing experimental data, suggesting the existence of two inactive states with the ionic lock formed and broken.[4–7,79]

Basin P corresponds to the pAs characterized by the IIL broken, the counterclockwise rotation of TM6, and the formation of the AIL. Such features indicate that the pAs is an active-intermediate conformation. This observation prompted us to investigate the binding of G protein to this receptor state performing docking and steered MD calculations. The docking simulations failed in providing a reliable $A_{2A}R$/G protein binary complex due to the steric clash occurring between the TM6 intracellular tail and G protein (Figure S17A). However, performing binding simulations including protein conformational flexibility, like in steered MD calculations, the G protein readily accesses the $A_{2A}R$ IBS, reaching the experimental $A_{2A}R$/G protein binary complex within a short simulation time that remains stable during unbiased MD calculations (Figure S18). It is noteworthy that the G protein binding to the pAs induces the opening of the TM6's intracellular end, without observed rotation of the helix, since TM6 is already rotated counterclockwise toward the active conformation in the pAs structure. Our findings indicate that the pAs structure represents a pre-coupling receptor conformation and might correspond to one of the intermediate-active states identified in previous studies for apo $A_{2A}R$.[5–7]

Basin M1 corresponds to an active-intermediate metastable state at higher energy value, similar to the pAs. Finally, state A represents the full active conformation of the receptor, capable of binding the G protein. In the absence of agonist ligands, this state exhibits a high free-energy value, resulting in a low conformational population, as also found in previous works.[5–7] As shown in Figure 6, ~90% of the structures representing this state exhibit the AIL between TM6 and TM5 formed, confirming this interaction as a hallmark of the receptor active states. We note that state A is very similar to the experimental active structure (PDB: 5G53[77]) and the active state previously identified in NMR studies.[5–7] Here, microswitch residues like NPxxY$^{7.49\text{-}53}$ assume the active conformation that, together with the formation of the AIL, facilitates the opening of the $A_{2A}R$ IBS for the G protein coupling (see Table S1).

The pAs in effector coupling

The identification of the pAs prompted us to investigate if such receptor state might play a functional role. To this end, we performed protein-protein docking calculations on pAs with the Gs protein (i.e., mini Gs). As previously mentioned, docking results indicated that the coupling of pAs with G protein is impeded by steric hindrance from the TM6 intracellular tail (Figure S17A), which significantly reduces the $A_{2A}R$ IBS's volume in the pAs (see Figure S8). However, when including receptor conformational flexibility in the binding simulations, as done in steered MD calculations, the G protein successfully binds to $A_{2A}R$ and adopts the experimental binding mode following rearrangement of the TM6 intracellular tail (Figure S18). Here, several interactions stabilize the complex, including salt bridges between the Gα's carboxylic C terminus of L394 and $A_{2A}R$'s K227$^{6.29}$ and between Gα's E392 and $A_{2A}R$'s R293$^{8.48}$. The $A_{2A}R$/G protein binding is further stabilized by a cation-π interaction formed between Gα's Y391 and R102$^{3.50}$ of the $A_{2A}R$'s E$^{6.30}$/DRY$^{3.49\text{-}51}$ motif. Notably, in the pAs/Gα complex, the Gα's Cα5 helix is slightly displaced outward, compared with the experimental structure (Figure S19), with a downward shift of ~2 Å measured between the Cα5 terminal part and the center of mass of the $A_{2A}R$'s NPxxY$^{7.49\text{-}53}$ motif (Figures S19C and S19D) Interestingly, a similar displacement of the Gα's Cα5 helix has recently been observed in an alternative active state of $A_{2A}R$ coupled with G protein, suggested to be implicated in rate-limited GDP/GTP exchange within the Gα protein without triggering full receptor activation.[101] Further investigations are necessary to comprehensively explore this phenomenon.

The coupling of $A_{2A}R$ with effectors other than G proteins remains a topic of debate in the literature.[102–104] While direct evidence is still forthcoming, it is beneficial to explore the structural basis of alternative $A_{2A}R$ signaling pathways involving non-G protein effectors. In this context, we investigated the binding interactions between pAs and β-arrestin 1. At variance with G protein, docking calculations reveal a high binding score between pAs and β-arrestin 1, consistent with previous structural investigations[105–107] showing that a reduced volume at the intracellular pocket might favor the interaction with β-arrestins. The resulting binary complex exhibits protein-protein interactions very similar to the cryoelectron microscopy (cryo-EM) structure of the β_1-adrenoreceptor (β_1AR) coupled with β-arrestin 1. Examples are the polar contacts between $A_{2A}R$'s R102$^{3.50}$ and N39$^{2.37}$ (R139$^{3.50}$ and Asn74$^{2.37}$ in β_1AR) with β-arrestin's E66 and the hydrophobic interactions formed by $A_{2A}R$'s I106$^{3.54}$ (I143$^{3.54}$ in β_1AR) and β-arrestin's L71 and L73 (Figures 5D, S17B, and S17C). The energetic and structural stability of the pAs/β-arrestin 1 complex was further assessed by plain MD calculations on the A_{2A}/β-arrestin binary complex identified in our study. In

CellPress
OPEN ACCESS

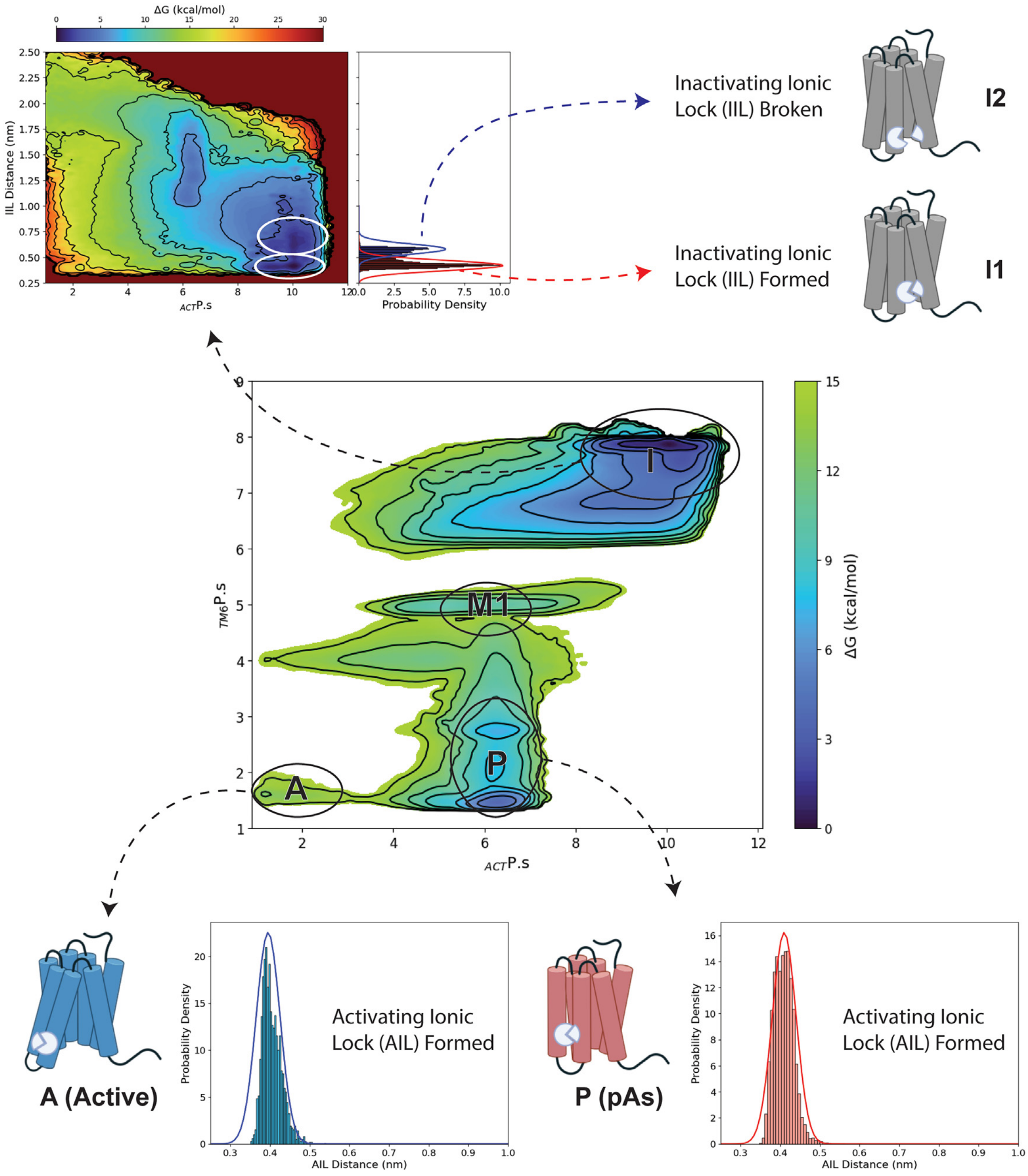

Figure 6. $A_{2A}R$ conformational states

The *apo* $A_{2A}R$ conformational states identified by the free-energy landscape calculated as a function of the $_{ACT}P.s$ and $_{TM6}P.s$ CVs (center). The inactive states I1 and I2 are characterized through a free-energy reweighting protocol using the inactive ionic lock distance as CV (defined as the distance between the $E^{6.30}$ Cδ and the $R^{3.50}$ Cζ) (upper left). The probability density of the inactivating and activating ionic locks (IIL and AIL) in the different states are reported as histograms.

particular, two independent simulations—each lasting 5 μs—were carried out. As can be seen in Figure S20, the A_{2A}/β-arrestin complex is stable throughout the simulations, with low RMSD values calculated for the protein's Cα atoms (1.8 ± 0.4 Å and 1.7 ± 0.4 Å for the two replicas, respectively). During the binding simulations, $A_{2A}R$ remains in the pAs, with the AIL formed over 90% of the simulation time (see Figures S20B and S21). It is important to note that phosphorylation of specific residues at the C-terminal tail, ICL3, or other sites of the receptor can enhance the binding affinity for β-arrestin. Owing to the absence of such detailed information, receptor phosphorylation was not considered in our study. Nevertheless, our findings suggest that the pAs can couple with β-arrestin and may contribute to β-arrestin-mediated activation of specific cellular pathways.

DISCUSSION

In this study, we have provided a thorough structural and energetic characterization of the activation mechanism of the adenosine GPCR A_{2A}R. Specifically, the *apo*, the *agonist-bound*, and the *inverse agonist-bound* forms of the receptor have been investigated. Among these, the A_{2A}R *apo* form is particularly interesting as it represents the basal functional state of the receptor for which no experimental structure has been reported so far. Our results show that the conformational ensemble of the receptor *apo* form is characterized by the co-presence of the inactive states (I1 and I2), active-intermediate states (M1 and P), and the fully activated state (A), in which P represents a *pseudo-active state* (pAs) that is disclosed here for the first time (Figures 5B and 6). This finding is in agreement with NMR and fluorescence-based studies that found the apo A_{2A}R in a similar distribution of states with the co-presence of the antagonist/inverse agonist-bound conformation and intermediate-active states.[5,15,108] In the pAs structure the receptor presents a mix of molecular features of the active and inactive state. The most important one is the salt bridge interaction established between E228$^{6.30}$ and R205$^{5.66}$—largely conserved within class A GPCRs—which stabilizes the TM6 active-like orientation, forming what we have defined *activating ionic lock* (AIL). The newly identified pAs can couple with the Gs protein upon minor rearrangement at the TM6 intracellular tail. In addition, it can bind β-arrestin 1, showing a binding mode very similar to the experimental one found between β_1R and β-arrestin 1 (Figures 5D and S17). In this regard, it is worth noting that A_{2A}R/β-arrestin binding is a rather unexplored area of research. Some studies have demonstrated A_{2A}R/β-arrestin binding and the ability of A_{2A}R to activate arrestin-mediated cellular pathways, even in the presence of the receptor apo form. For instance, the works of Franco and colleagues[102,103] have shown the role of arrestin recruitment by A_{2A}R in receptor internalization and triggering G protein-alternative cell signaling. Despite these efforts, the precise mechanism of interaction between A_{2A}R and β-arrestin, including the phosphorylation sites at A_{2A}R,[109–111] remains poorly understood. In this context, here, the reported structure of the complex between A_{2A}R and β-arrestin provides valuable insights, establishing a structural basis of A_{2A}R/β-arrestin binding interaction. Our findings open novel opportunities for modulation of A_{2A}R activity, also in terms of receptor desensitization and activation of G protein-alternative cellular pathways.[109–111] For instance, the pAs might be targeted to identify novel A_{2A}R-biased ligands by using standard and advanced drug discovery campaigns that employ AI-based algorithms like graph neural networks and geometrical deep learning to increase the hit-discovery success rate. In addition, the pAs might be helpful in elucidating the yet unclear molecular aspects of β-arrestin coupling to A_{2A}R, including phosphorylation at the A_{2A}R's C terminus.[109,112,113]

When A_{2A}R is bound to the agonist NECA without G protein, the receptor is found in two most probable states (inactive [I] and active [A], Figure 5B). The first one corresponds to the inactive-like structure of the receptor, in agreement with the reported X-ray and cryo-EM agonist-bound structures of A_{2A}R[82–84,114] and as also found for the β_2-adrenoreceptor by structural and computational studies.[46,115] In this regard, it is worth noting that the experimental agonist-A_{2A}R complexes (in the absence of the G protein) are defined as intermediate-active states, even though all of them have TM6 rotated clockwise in the inactive form and the intracellular region of the receptor close to the inactive state. In fact, plotting the position of the experimental agonist-bound A_{2A}R structures[81–84,114] onto the FES of the activation mechanism, they all result close to the inactive state I (Figure S22). The second low energy state instead corresponds to the receptor active form, characterized by the counterclockwise rotation of TM6 and the presence of the AIL. This indicates that the agonist binding—even in the absence of the G protein—induces conformational changes in A_{2A}R leading to the loss of pAs in favor of the active form (A), which is competent for the G protein binding. Our finding rationalizes the ^{19}F-NMR data of agonist-bound and apo A_{2A}R reported by Huang et al.[5] (Figure S23), which show in both cases the A_{2A}R in equilibrium between multiple states, among which one is always represented by the inactive state, whereas the others (i.e., active and pAs) are differently stabilized by the presence of the agonist. Furthermore, our results suggest that the "*A-I equilibrium*" established upon agonist binding is functional for the definitive activation of the receptor—and stabilization of the active form—occurring only after the G protein recruitment at the receptor IBS. Interestingly, a recent study by Solano and Choi investigated the activation mechanism of the A_1R in the presence of the agonist adenosine.[58] This receptor shares a relatively high identity percentage with A_{2A}R (~38% for the whole sequence), and it is worth noting some similarities between our results on A_{2A}R and those on A_1R. Similarly to what we observed for A_{2A}R, the authors found that A_1R activation is characterized by the roto-translational motion of TM6, and the inactive state is the lowest energy minimum in the receptor agonist-bound form with the co-presence of a pre-active state.

Finally, when the receptor is bound to ZMA, the basal "*P-I equilibrium*" is shifted toward the inactive conformation, which is the only low energy state present (Figure 5A). Notably, the intrinsic pharmacological activity of ZMA has been a subject of debate in the literature, with some studies classifying it as a neutral antagonist,[60–62] while more recent research attributes an inverse agonist activity to ZMA.[5,6,63] It is important to recognize that the characterization of ligand pharmacological activity can vary depending on the specific system under investigation and the experimental conditions employed. However, drawing from the widely accepted understanding that a neutral antagonist competes with agonists for binding without affecting the receptor's basal activity, whereas an inverse agonist diminishes basal activity, our findings indicate that ZMA functions as an inverse agonist by stabilizing the A_{2A}R in its inactive form. Indeed, if ZMA had acted as a neutral antagonist, it would not have influenced the two-states equilibrium of the A_{2A}R *apo* form.

Our study provides unprecedented structural and energetic insights into A_{2A}R activation mechanism, revealing a novel receptor state, pAs, which could be further investigated in biased signaling studies and could be targeted by drug design campaigns to develop A_{2A}R biased ligands. Our simulation protocol is generalizable and can be applied to study the activation mechanism of any GPCRs and to predict the intrinsic activity of ligands

based on their effect on the receptor conformational dynamics, resulting in a valuable tool for investigations on GPCR activation and drug design.

EXPERIMENTAL PROCEDURES

Systems setup and unbiased MD

According to the best resolution criterion, the starting conformations of the active and inactive $A_{2A}R$ have been taken, respectively, from the PDB: 5G53[77] and PDB: 3PWH[4] X-ray structures, whereas the coordinates of the mini-Gs protein heterotrimer were extracted from the cryo-EM structure PDB: 6GDG[78] (after the alignment of the GPCR section with 5G53[77]). The active G protein-uncoupled (*FAN*, *FApo*) and the apo (*FApo*, *FApoG*, and *IN-apo*) systems were obtained by removing the engineered G protein or the co-crystalized orthosteric ligands (or both) from the original structures (PDB: 5G53,[77] 3PWH[4]). To prepare the *INeca* and *ACzma* systems, we first aligned the agonist- and antagonist-bound $A_{2A}R$ and then replaced the antagonist ZMA in the inactive receptor conformation (PDB: 3PWH[4]) with the agonist NECA, as well as the agonist NECA with the antagonist ZMA in the active receptor conformation (PDB: 5G53[77]). Any mutation present in the starting PDB structures was converted to its wild-type form. Before MD simulations, the receptor's first N-terminal (S5) and the last C-terminal (S305) residues were capped with acetyl and N-methyl groups, respectively. The receptor conformations were prepared using the Protein Preparation Wizard tool, implemented in the Maestro Suite 2021.[116] Any $A_{2A}R$ missing residue was added and conformationally optimized using the Prime toolkit[117,118] and one of the aforementioned PDB structures as reference. Mutations were reverted in accordance with the $A_{2A}R$ primary sequence taken from the UNIPROT database (UNIPROT: P29274). Missing loops were recreated during the refinement stage of Prime by randomly generating starting conformations and then further optimizing their orientation and position via an iterative, energy-based process embedded in Prime. The top scoring loops were selected for the final $A_{2A}R$ structures. A similar approach was followed to generate the missing side chains. All the optimization calculations were carried out using an energy-based approach and the OPLS 2005 all-atom force field. The missing parts of the proteins were first minimized via rigid-body simulation and then further optimized via hybrid Monte Carlo conformational sampling. The implicit membrane treatment was applied when needed.

The residue protonation states were evaluated using Maestro Epik[119,120] toolkit. This approach provides an estimation of the side-chain pKa via iterative calculations. Starting from the optimized structures obtained as previously described, initial protonation states were assigned at pH 7. Then, the pKa of each acidic side chain was re-evaluated after removal of its acid hydrogens. This process was repeated for all acid groups. Then, the initial structure was regenerated, and the basic groups were protonated one at a time following a similar procedure. This computational titration assay allows for properly evaluating the most probable protonation state of each residue at the chosen pH by estimating the variation of its pKa in the protonated and deprotonated states.

The final structures were then subjected to another round of rigid-body and hybrid Monte Carlo minimization to further optimize the receptor structure and the residue interactions. During this phase, special care was provided to the hydrogen-bond network. To do so, initially, only the hydrogens' positions were optimized by imposing a restraint on all the heavy atoms. Afterward, these constraints were released, and the full structure underwent a new cycle of energy minimization.

Each optimized receptor conformation was then embedded in a 105 × 105 Å (along x and y axes) pre-equilibrated POPC—cholesterol (7:3 molar ratio) bilayer and solvated using the TIP3P water model with the aid of the membrane-builder tool of CHARMM-GUI.org (http://www.charmm-gui.org). The ff14SB and lipid17 Amber force fields were used to parametrize the protein and the lipids, respectively.[121] As for the GPD molecule and for the two orthosteric ligands, namely NECA and ZMA, different force fields were used based on the distinct chemical nature of the three compounds. Specifically, a combination of Amber OL3[122] and generalized amber force field (GAFF[123]) parameters were adopted for GPD and NECA, while the GAFF[123] alone was used to treat ZMA. Their atomic partial charges were instead computed using the two-staged restrained electrostatic potential (RESP)[124,125] fitting procedure implemented in Antechamber.[126] The electrostatic potentials (ESPs) were first calculated through the quantomechanical package Gaussian 16.[127] Specifically, the adopted protocol included a double-step geometry optimization procedure at Hartree-Fock level of theory: (1) a preliminary calculation with the 3–21G basis set, followed by (2) a more accurate procedure with the 6–31G* basis set, after which the ESPs were computed. The topology files of the systems were obtained with the tleap program of AmbertTools20[128] and then converted into the GROMACS format by the means of ParmEd. The GROMACS 2020.6[129] code was used to perform the simulations. A cutoff of 12 Å was used for short-range interactions. The long-range electrostatic interactions were computed through the particle mesh Ewald method[130] using a 1.0-Å grid spacing in periodic boundary conditions. The non-iterative LINCS[131] algorithm was applied to constraint bonds, which allowed using a 2-fs integration time step. To solve all the steric clashes, each system underwent 30,000 steps of steepest descent energy minimization in three phases. In the first one, the system heavy atoms were kept fixed to relax only the hydrogens and the water molecules; during the second stage, also the lipidic bilayer was released; and in the third step all the atomic positions were minimized. Then, each complex was equilibrated and heated up to 300 K. Our equilibration protocol follows an iterative approach composed of isothermal-isobaric (NPT) and canonical (NVT) ensemble simulations to ensure a smooth and uniform equilibration of the atom distribution and box sizes. Starting from the temperature of 50 K, the system is first simulated in the NPT ensemble at 1 atm for 1 ns and then in the NVT ensemble for the same time to let the solvent rearrange itself and to avoid creation of low-density regions in the box. Velocities are only generated during this NPT step and then inherited from run to run. Since the addition of the membrane bilayer may introduce steric clashes that might not be resolved during the energy minimization phase, the integrator time step is reduced to 1 fs for the 50-K NPT/NVT cycle only. The temperature is then increased by 50 K, and the procedure is repeated until the temperature reaches 250 K. After this point, the temperature increase is lowered to 25 K for each NPT/NVT cycle. This was done to ensure that the system heats slowly after approaching the water melting temperature. In semi-isotropic systems such as the ones containing lipid bilayers, this is a critical step because of the change in the solvent fluidity that may affect the membrane structure. During the whole equilibration phase, position restraints are applied to the protein and the ligand. An initial force constant of 1,000 kJ/mol is applied during the first 50-K NPT/NVT cycle and then progressively lowered by 160 kJ/mol with each increase of temperature to ensure a smooth equilibration of the protein/ligand complex. Following this approach, a low force constant of 40 kJ/mol is maintained after reaching the 300-K threshold. Another cycle of NPT/NVT simulations at 300 K is then repeated without the position restraints to allow the protein and ligand to move freely. For the whole equilibration procedure, Berendsen thermostat and barostat[132] are applied to avoid abrupt variations in the system's temperature and pressure. Before proceeding with the production simulations, these algorithms are switched to the stochastic velocity rescaling[133] and the Parrinello-Rahman,[134] respectively. A 10-ns NPT preproduction run at 300 K with these settings is performed to remove the influence of the starting conditions.

The same equilibration and production protocols were used also for the simulations on the $A_{2A}R$/β-arrestin and $A_{2A}R$/G protein complexes, coming from protein-protein docking and steered MD calculations, respectively.

Regarding the RMSD and volume analyses shown in Figure 2 and in the supplemental information, the different regions of the receptor have been defined as follows: (1) OBS residues (Cα atoms) 8–14, 57–66, 75–90, 132–140, 175–187, 245–258, 267–278; (2) connector residues (Cα atoms) 15–23, 50–56, 91–97, 127–131, 188–197, 236–244, 279–283; (3) IBS residues (Cα atoms) 24–31, 42–49, 98–106, 119–126, 198–208, 222–235, 283–287 (residue numbering: UNIPROT: P29274).

Cross-correlation analysis

Cross-correlation analysis (or Pearson-correlation coefficient analysis) was used to assess the correlated motions between pairs of residues in the seven simulated MD systems (Table 1). An in-house code was employed to calculate the Pearson coefficients matrices according to the following formula:

$$C_{ij} = \frac{\langle (x_i - \langle x_i \rangle)(x_j - \langle x_j \rangle) \rangle}{\sigma_{x_i} \sigma_{x_j}} \quad \text{(Equation 1)}$$

where the numerator is the covariance between two variables, $\boldsymbol{x_i}$ and $\boldsymbol{x_j}$, while $\boldsymbol{\sigma_i}$ and $\boldsymbol{\sigma_j}$ are the standard deviations of each variable. The normalization obtained dividing the covariance by the product of the standard deviation of the variables allows having values ranging between −1 and +1. The variables represent the Cα atoms' positional vectors, and the Pearson correlation coefficients have been evaluated between any pairs of Cα atoms.

PSN analysis

Network parameters such as hubs, communities, and structural communication analyses were obtained by using the WebPSN 2.0 webserver.[135–137] The methodology builds the protein structure graph (PSG) based on the interaction strength of two connected nodes:

$$I_{ij} = \frac{n_{ij}}{\sqrt{N_i N_j}} 100 \quad \text{(Equation 2)}$$

where interaction percentage ($\boldsymbol{I_{ij}}$) of nodes $\boldsymbol{i}$ and $\boldsymbol{j}$ represents the number of pairs of side-chain atoms within a given cutoff value (4.5 Å), while $\boldsymbol{N_i}$ and $\boldsymbol{N_j}$ are normalization factors. The interaction strength (represented as a percentage) between residues $\boldsymbol{i}$ and $\boldsymbol{j}$ ($\boldsymbol{I_{ij}}$) is calculated for all node pairs. If $\boldsymbol{I_{ij}}$ is more than the minimum interaction strength cutoff ($\boldsymbol{I_{min}}$) among the residue pairs, then the nodes are considered to be interacting and hence represented as a connection in the PSG.

WT-MetaD with PCVs

MetaD[90] is an enhanced sampling method in which the simulation is boosted by a Gaussian-shaped history-dependent bias potential (V_G), deposited on a selected number of reaction coordinates (i.e., slow degrees of freedom) of the system, usually referred to as collective variables (CVs):

$$V_G(S,t) = \int_0^t dt' \omega \exp - \sum_{i=1}^{d} \frac{(S_i(R) - S_i(R(t')))^2}{2\sigma_i^2} \quad \text{(Equation 3)}$$

where $\boldsymbol{S_i}$ is the value of the ith CV, $\boldsymbol{\sigma_i}$ is the width of the Gaussian function, and ω is the rate at which the bias is deposited. WT-MetaD[91] is an evolution of the method in which the bias deposition rate ω is exponentially rescaled over time, depending on how much potential has already been added in the same region of the CV phase space, according to following formula:

$$W = \omega_0 \tau_G e^{-\frac{V_G(\mathbf{S},t)}{k_B \Delta T}} \quad \text{(Equation 4)}$$

where $\boldsymbol{W}$ is the Gaussian height, $\boldsymbol{k_B}$ is Boltzmann's constant, $\boldsymbol{\omega_0}$ is the initial energy rate, $\boldsymbol{\tau_G}$ is the Gaussian deposition stride, $\boldsymbol{\Delta T}$ is the fictitious temperature at which the biased CV ($\boldsymbol{S}$) is sampled, and $\boldsymbol{V_G(S, t)}$ is the bias potential accumulated in $\boldsymbol{S}$ over time $\boldsymbol{t}$. At the end of a WT-MetaD simulation, the deposited bias potential $\boldsymbol{V_G}$ asymptotically converges to the inverse value of a fraction of the free energy $\boldsymbol{F(S)}$:

$$V_G(S, t \to \infty) = -\frac{\Delta T}{\Delta T + T} F(S) \quad \text{(Equation 5)}$$

The fictitious temperature $\boldsymbol{\Delta T}$ is the parameter that controls how quickly the Gaussian height is decreased and often is written in terms of the so-called bias factor $\gamma = \boldsymbol{(T + \Delta T)/T}$. The acceleration factor α introduced by the underlying MetaD bias deposited during the simulations was computed as $\alpha = e^{\Delta F(S,t)/kBT}$ [138] using the energetic difference $\boldsymbol{\Delta F}$ calculated between the lowest-energy minimum and the highest energy transition state identified in the *apo* and *NECA-bound* systems (14.5 and 13.4 kcal/mol, respectively).

The large-scale conformational differences between the crystal structures of the active and inactive $A_{2A}R$ suggest that the transition between these states is highly cooperative and involves a number of degrees of freedom. For this reason, the use of simple geometrical CVs (i.e., a distance or a torsion) might be insufficient both to reproduce the event and to calculate the associated free energy. To overcome this limitation, we employed the PCVs approach,[92] which has been successfully applied in a number of conformational transition studies.[93–96] In this dimensionality reduction scheme, two functions are used to characterize the position of a point in a configurational space **R** relative to a preassigned path $\boldsymbol{l}$ in terms of progress along the path $\boldsymbol{s}$(**R**) and distance from it $\boldsymbol{z}$(**R**):

$$s(\mathrm{R}) = \frac{1}{N-1} \frac{\sum_{i=1}^{N} (i-1) \exp\left(-\lambda \|X(\mathrm{R}) - X(l)\|^2\right)}{\sum_{i=1}^{N} \exp\left(-\lambda \|X(\mathrm{R}) - X(l)\|^2\right)} \quad \text{(Equation 6)}$$

$$z(\mathrm{R}) = -\frac{1}{\lambda} \ln\left[\sum_{i=1}^{N} \exp\left(-\lambda \|X(\mathrm{R}) - X(l)\|^2\right)\right] \quad \text{(Equation 7)}$$

where $\boldsymbol{X(R)}$ is a reduced representation of $\boldsymbol{R}$, $\boldsymbol{X(l)}$ is the same kind of reduced representation of the path $\boldsymbol{l}$, $\boldsymbol{i}$ is a discrete index ranging from 1 to $\boldsymbol{N}$, with $\boldsymbol{N}$ being the number of conformations selected to build the path. ‖…‖ indicates the metric used to compute the distance between the configurations, which is generally defined in terms of contacts matrix or RMSD. In this work, a preliminary guess of the $A_{2A}R$ activation process was obtained through the unbiased MD system *FApo* (transition from the active state to the pAs) and steered MD (transition from the pAs to the inactive state). The latter was performed applying a constant force (harmonic constant k = 4.774 kcal/mol) on a CV represented by the RMSD of the protein Cα atoms computed with respect to the experimental inactive conformation of $A_{2A}R$. The overall trajectory was then used to extract the frames needed for the preassigned paths of two sets of PCVs: P_{ACT} and P_{TM6}.

$_{ACT}$PCVs

In $_{ACT}$PCVs, $\boldsymbol{X(R)}$ is defined as a set of Cartesian coordinates belonging to a subset of atoms. The distance ‖…‖ of each generic configuration $\boldsymbol{X(R)}$ from the path was computed as the RMSD of the subset after optimal receptors' alignment by using Kearsley's algorithm.[139] Notably, the choice of the atoms to be included in the path is far from trivial; in fact, a wrong choice can turn into a loss of performance and additional noise that may affect the calculations. Here, we used the Cα and Cβ of the most important receptor microswitches (PIF, toggle, NPxxY, AIL, IIL) as well as the Cζ and Cγ of key residues detected in our unbiased MD (Table S2; Figure S11A). The final path consists of 12 frames: 10 frames directly extracted from the preliminary deactivation trajectory (obtained as described above) and two terminal extra-(non-real)-frames. We verified that the obtained set of configurations was equally spaced in the adopted mean square displacement metrics, and the value of λ was chosen so as to be comparable to the inverse of the RMSD between successive frames. The average distance between adjacent frames was 0.74 Å, which thus required setting λ = 1.22 $Å^{-2}$ in Equations 2 and 3.

$_{TM6}$PCVs

Here, the reduced representation $\boldsymbol{X(R)}$ is defined as the CMAP matrix $\boldsymbol{C(R)}$ and the distance ‖…‖ computed as:

$$\|X(\mathbf{R}) - X(l)\| = \sum_{j>1} \left[C(\mathbf{R})_{i,j} - C(\boldsymbol{l})_{i,j}\right]^2 \quad \text{(Equation 8)}$$

where $\boldsymbol{C(R)_{i,j}}$ and $\boldsymbol{C(l)_{i,j}}$ are the elements of the CMAP matrix. A contact between atom $\boldsymbol{i}$ and $\boldsymbol{j}$ is defined as

$$C(\mathbf{R})_{i,j} = \frac{1 - \left(\frac{r_{ij}}{r_0}\right)^n}{1 - \left(\frac{r_{ij}}{r_0}\right)^m} \quad \text{(Equation 9)}$$

where $\boldsymbol{r_{i,j}}$ is the distance between the two atoms, and $\boldsymbol{r_0}$ is the typical distance at which the contact is formed (Table S3). The list of the contacts included (Table S3) in the CV was chosen to specifically accelerate the around-the-axis rotation of TM6 occurring upon receptor (de)activation. The final path consists of 7 frames. However, The FESs in Figures S16 and S22 are computed

through a reweighting procedure[100] as a function of path CVs defined by the $_{TM6}$PCV used in the production runs with the addition of two terminal extra fames that might represent multiple receptor conformations sampled at the endpoints. The average distance between adjacent frames was 0.63, which thus required setting λ = 3.64, according to the same criterion used for S_{ACT}.

The PLUMED 2.7.1[140,141] library patched with the GROMACS 2020.6[129] MD engine was used to perform WT-MetaD simulations on the uncoupled A_{2A}R in its *apo*, *NECA-bound*, and *ZMA-bound* forms. Two-dimensional Gaussians were added on the ***s*(R)** components of $_{ACT}$PCVs *and* $_{TM6}$PCVs every 2 ps. An initial Gaussian height of 0.95 kcal/mol was gradually decreased based on a bias factor γ = 15. The Gaussian widths were respectively set to 0.1 and 0.03 for the $_{ACT}$P.*s* and $_{TM6}$P.*s* dimensions, according to the CVs' fluctuations observed in the standard MD regime. To limit the exploration of unphysical states during the simulations, harmonic restraints were placed on the helicity of TM6, based on the ALPHARMSD variable defined in PLUMED. The bias reweighting procedures used along this work were performed according to the algorithm developed by Bonomi et al.[100]

Protein-protein docking

To evaluate the possible affinity of the newly discovered A_{2A}R pAs toward different intracellular transducers such as mini Gs and β-arrestin 1, protein-protein docking were performed with the aid of the HADDOCK 2.4 webserver.[142,143] Specifically, the minimized structure of the pAS extracted from our MD simulations was used as starting conformation for the A_{2A}R, while the 3D coordinates of the mini Gs and β-arrestin 1 were taken from the experimental PDB: 6GDG[78], 6TKO.[106]. Prior to docking, we indicated, as active residues of the A_{2A}R, the following amino acids defining the IBS: 106, 102, 203, 235, 230, 227, 292, 208. On the other hand, based on the analysis of multiple GPCR-G protein and GPCR-β-arrestin complexes, we indicated, as interacting portion of the transducers, the α5 helix of mini Gs (residues: 239–248) and the finger loop of β-arrestin 1 (residues: 63–76). The best binding poses were selected as those having the lowest RMSD with respect to 6TKO PDB complex.

Steered MD

The coupling process of the mini Gs (α subunit) to the A_{2A}R pAs was investigated through the steered MD implementation included in the PLUMED platform. Particularly, a time-dependent harmonic restraint was applied on the system RMSD with respect to a target configuration, according to the following formula:

$$V(\vec{s},t) = \frac{1}{2}\kappa(t)\left(\vec{s} - \vec{s}_0(t)\right)^2$$

where *k* is the force constant, and s_0 is the target conformation. These two values are iteratively tuned by PLUMED at each simulation step to reach the target value in a user-defined number of steps.

Our protocol was thus divided into two steps, each lasting 20 ns. In the first, the steering force was applied on a RMSD-based CV, computed on the Cα atoms of HN (residues 14–40, UNIPROT: P63092), α4 (residues 323–341, UNIPROT: P63092) and α5 (residues 361–381, UNIPROT: P63092) helices of the mini Gs (α subunit) after optimal alignment on the A_{2A}R OBS (Cα atoms of residues 8–14, 57–66, 75–90, 132–140, 175–187, 245–258, 267–278, according to the UNIPROT: P29274 numeration).

In the second step, the intermolecular contacts were refined by computing the RMSD CV on the Cβ atoms of all the secondary structure elements of the mini Gs α subunit (residues 14–46, 209–214, 217–223, 243–249, 253–268, 277–293, 322–341, 349–353, 360–364; UNIPROT: P63092) after optimal alignment on the Cα atoms of the TM helices of A_{2A}R (Cα atoms of residues 8–31, 42–66, 75–106, 119–140, 175–205, 227–257, 267–287; UNIPROT: P29274). In both the steered MD steps, a kappa of 10,000 kJ/mol was used.

RESOURCE AVAILABILITY

Lead contact

Further information and requests for resources and materials should be directed to and will be fulfilled by the lead contact, Vittorio Limongelli (vittoriolimongelli@gmail.com).

Materials availability

This study did not generate new unique reagents. All data and codes are available and can be found at the addresses specified in the data and code availability section.

Data and code availability

- The Path CV MetaD protocol employed in this work is available on PLUMED-NEST (plumID:23.045).
- The structure of the A_{2A}R pAs is available as PDB file in the supplemental information and at www.pdbdb.com.
- The complete dataset of the simulations and codes used or generated in this study is available on Zenodo (DOI: 10.5281/zenodo.13460724).
- The movie of the activation mechanism of A_{2A}R in apo and ligated forms is available as supplemental information and at https://youtu.be/TbXi3KjIWFo.

ACKNOWLEDGMENTS

This work has received funding from the European Research Council (ERC) under the European Union's Horizon 2020 research and innovation programme ("CoMMBi" ERC grant agreement no. 101001784), and it was supported by a grant from the Swiss National Supercomputing Centre (CSCS) under project ID u8 and s1150. The authors also thank Dr. Francesco Saverio Di Leva from the University of Naples "Federico II" and Dr. Stefano Raniolo from the Università della Svizzera italiana (USI) for reading the paper and for the useful discussions, as well as Daniele Angioletti for providing the equivariant graph neural network utilized to calculate NMR chemical shifts.

AUTHOR CONTRIBUTIONS

Conceptualization, V.L.; methodology, V.M.D., P.C., L.M., and V.L.; investigation, V.M.D.; software, V.M.D.; visualization, V.M.D., P.C., and V.L.; data curation, V.M.D., P.C., and V.L.; writing – original draft, V.M.D., P.C., L.M., and V.L.; writing – review & editing, V.M.D., P.C., L.M., and V.L.; resources, V.M.D., P.C., and V.L.; funding acquisition, V.L.; supervision, V.L.

DECLARATION OF INTERESTS

The authors declare no competing interests.

SUPPLEMENTAL INFORMATION

Supplemental information can be found online at https://doi.org/10.1016/j.chempr.2024.08.004.

Received: December 21, 2023
Revised: July 11, 2024
Accepted: August 8, 2024
Published: September 9, 2024

REFERENCES

1. Rosenbaum, D.M., Rasmussen, S.G.F., and Kobilka, B.K. (2009). The structure and function of G-protein-coupled receptors. Nature *459*, 356–363. https://doi.org/10.1038/nature08144.
2. Yang, D., Zhou, Q., Labroska, V., Qin, S., Darbalaei, S., Wu, Y., Yuliantie, E., Xie, L., Tao, H., Cheng, J., et al. (2021). G protein-coupled receptors: structure- and function-based drug discovery. Signal Transduct. Target. Ther. *6*, 7. https://doi.org/10.1038/s41392-020-00435-w.
3. Hilger, D., Masureel, M., and Kobilka, B.K. (2018). Structure and dynamics of GPCR signaling complexes. Nat. Struct. Mol. Biol. *25*, 4–12. https://doi.org/10.1038/s41594-017-0011-7.
4. Doré, A.S., Robertson, N., Errey, J.C., Ng, I., Hollenstein, K., Tehan, B., Hurrell, E., Bennett, K., Congreve, M., Magnani, F., et al. (2011). Structure of the adenosine A 2A receptor in complex with ZM241385 and the

xanthines XAC and caffeine. Structure *19*, 1283–1293. https://doi.org/10.1016/j.str.2011.06.014.

5. Huang, S.K., Pandey, A., Tran, D.P., Villanueva, N.L., Kitao, A., Sunahara, R.K., Sljoka, A., and Prosser, R.S. (2021). Delineating the conformational landscape of the adenosine A2A receptor during G protein coupling. Cell *184*, 1884–1894.e14. https://doi.org/10.1016/j.cell.2021.02.041.
6. Ye, L., Van Eps, N., Zimmer, M., Ernst, O.P., and Prosser, R.S. (2016). Activation of the A 2A adenosine G-protein-coupled receptor by conformational selection. Nature *533*, 265–268. https://doi.org/10.1038/nature17668.
7. Wang, X., Neale, C., Kim, S.K., Goddard, W.A., and Ye, L. (2023). Intermediate-state-trapped mutants pinpoint G protein-coupled receptor conformational allostery. Nat. Commun. *14*, 1325. https://doi.org/10.1038/s41467-023-36971-6.
8. Weis, W.I., and Kobilka, B.K. (2018). The Molecular Basis of G Protein-Coupled Receptor Activation. Annu. Rev. Biochem. *87*, 897–919. https://doi.org/10.1146/annurev-biochem-060614-033910.
9. Hauser, A.S., Kooistra, A.J., Munk, C., Heydenreich, F.M., Veprintsev, D.B., Bouvier, M., Babu, M.M., and Gloriam, D.E. (2021). GPCR activation mechanisms across classes and macro/microscales. Nat. Struct. Mol. Biol. *28*, 879–888. https://doi.org/10.1038/s41594-021-00674-7.
10. Venkatakrishnan, A.J., Deupi, X., Lebon, G., Tate, C.G., Schertler, G.F., and Babu, M.M. (2013). Molecular signatures of G-protein-coupled receptors. Nature *494*, 185–194. https://doi.org/10.1038/nature11896.
11. Manglik, A., and Kruse, A.C. (2017). Structural Basis for G Protein-Coupled Receptor Activation. Biochemistry *56*, 5628–5634. https://doi.org/10.1021/acs.biochem.7b00747.
12. Rasmussen, S.G.F., Choi, H.J., Fung, J.J., Pardon, E., Casarosa, P., Chae, P.S., Devree, B.T., Rosenbaum, D.M., Thian, F.S., Kobilka, T.S., et al. (2011). Structure of a nanobody-stabilized active state of the β2 adrenoceptor. Nature *469*, 175–180. https://doi.org/10.1038/nature09648.
13. Rosenbaum, D.M., Zhang, C., Lyons, J.A., Holl, R., Aragao, D., Arlow, D.H., Rasmussen, S.G.F., Choi, H.J., Devree, B.T., Sunahara, R.K., et al. (2011). Structure and function of an irreversible agonist-β2 adrenoceptor complex. Nature *469*, 236–240. https://doi.org/10.1038/nature09665.
14. Smith, J.S., Lefkowitz, R.J., and Rajagopal, S. (2018). Biased signalling: From simple switches to allosteric microprocessors. Nat. Rev. Drug Discov. *17*, 243–260. https://doi.org/10.1038/nrd.2017.229.
15. Prosser, R.S., Ye, L., Pandey, A., and Orazietti, A. (2017). Activation processes in ligand-activated G protein-coupled receptors: A case study of the adenosine A2A receptor. BioEssays *39*, 1–10. https://doi.org/10.1002/bies.201700072.
16. Warne, T., Edwards, P.C., Doré, A.S., Leslie, A.G.W., and Tate, C.G. (2019). Molecular basis for high-affinity agonist binding in GPCRs. Science *364*, 775–778. https://doi.org/10.1126/science.aau5595.
17. Neves, S.R., Ram, P.T., and Iyengar, R. (2002). G protein pathways. Science *296*, 1636–1639. https://doi.org/10.1126/science.1071550.
18. Oldham, W.M., and Hamm, H.E. (2008). Heterotrimeric G protein activation by G-protein-coupled receptors. Nat. Rev. Mol. Cell Biol. *9*, 60–71. https://doi.org/10.1038/nrm2299.
19. Hurowitz, E.H., Melnyk, J.M., Chen, Y.J., Kouros-Mehr, H., Simon, M.I., and Shizuya, H. (2000). Genomic characterization of the human heterotrimeric G protein α, β, and γ subunit genes. DNA Res. *7*, 111–120. https://doi.org/10.1093/dnares/7.2.111.
20. Krasel, C., and Lohse, M.J. (2007). G protein coupled receptor kinases. In The Comprehensive Pharmacology Reference, S.J. Enna and D.B. Bylund, eds. (Elsevier), pp. 1–19. https://doi.org/10.1016/B978-008055232-3.63085-5.
21. McGarrigle, D., and Huang, X.Y. (2007). GPCRs signaling directly through Src-family kinases. Sci. STKE *2007*, pe35. https://doi.org/10.1126/stke.3922007pe35.
22. Smith, J.S., and Rajagopal, S. (2016). The β-Arrestins: Multifunctional regulators of G protein-coupled receptors. J. Biol. Chem. *291*, 8969–8977. https://doi.org/10.1074/jbc.R115.713313.
23. Shiraishi, Y., Kofuku, Y., Ueda, T., Pandey, S., Dwivedi-Agnihotri, H., Shukla, A.K., and Shimada, I. (2021). Biphasic activation of β-arrestin 1 upon interaction with a GPCR revealed by methyl-TROSY NMR. Nat. Commun. *12*, 7158. https://doi.org/10.1038/s41467-021-27482-3.
24. Slosky, L.M., Caron, M.G., and Barak, L.S. (2021). Biased Allosteric Modulators: New Frontiers in GPCR Drug Discovery. Trends Pharmacol. Sci. *42*, 283–299. https://doi.org/10.1016/j.tips.2020.12.005.
25. Franco, R., Rivas-Santisteban, R., Reyes-Resina, I., and Navarro, G. (2021). The Old and New Visions of Biased Agonism Through the Prism of Adenosine Receptor Signaling and Receptor/Receptor and Receptor/Protein Interactions. Front. Pharmacol. *11*, 1–6. https://doi.org/10.3389/fphar.2020.628601.
26. Wootten, D., Christopoulos, A., Marti-Solano, M., Babu, M.M., and Sexton, P.M. (2018). Mechanisms of signalling and biased agonism in G protein-coupled receptors. Nat. Rev. Mol. Cell Biol. *19*, 638–653. https://doi.org/10.1038/s41580-018-0049-3.
27. Hauser, A.S., Attwood, M.M., Rask-Andersen, M., Schiöth, H.B., and Gloriam, D.E. (2017). Trends in GPCR drug discovery: New agents, targets and indications. Nat. Rev. Drug Discov. *16*, 829–842. https://doi.org/10.1038/nrd.2017.178.
28. Cooray, S.N., Gobbetti, T., Montero-Melendez, T., McArthur, S., Thompson, D., Clark, A.J.L., Flower, R.J., and Perretti, M. (2013). Ligand-specific conformational change of the G-protein-coupled receptor ALX/FPR2 determines proresolving functional responses. Proc. Natl. Acad. Sci. USA *110*, 18232–18237. https://doi.org/10.1073/pnas.1308253110.
29. Wingler, L.M., Skiba, M.A., McMahon, C., Staus, D.P., Kleinhenz, A.L.W., Suomivuori, C.M., Latorraca, N.R., Dror, R.O., Lefkowitz, R.J., and Kruse, A.C. (2020). Angiotensin and biased analogs induce structurally distinct active conformations within a GPCR. Science *367*, 888–892. https://doi.org/10.1126/science.aay9813.
30. Slosky, L.M., Bai, Y., Toth, K., Ray, C., Rochelle, L.K., Badea, A., Chandrasekhar, R., Pogorelov, V.M., Abraham, D.M., Atluri, N., et al. (2020). β-Arrestin-Biased Allosteric Modulator of NTSR1 Selectively Attenuates Addictive Behaviors. Cell *181*, 1364–1379.e14. https://doi.org/10.1016/j.cell.2020.04.053.
31. Gurevich, V.V., and Gurevich, E.V. (2020). Biased GPCR signaling: Possible mechanisms and inherent limitations. Pharmacol. Ther. *211*, 107540. https://doi.org/10.1016/j.pharmthera.2020.107540.
32. Wisler, J.W., Rockman, H.A., and Lefkowitz, R.J. (2018). Biased G protein-coupled receptor signaling: Changing the paradigm of drug discovery. Circulation *137*, 2315–2317. https://doi.org/10.1161/CIRCULATIONAHA.117.028194.
33. Poulie, C.B.M., Pottie, E., Simon, I.A., Harpsøe, K., D'Andrea, L., Komarov, I.V., Gloriam, D.E., Jensen, A.A., Stove, C.P., and Kristensen, J.L. (2022). Discovery of β-Arrestin-Biased 25CN-NBOH-Derived 5-HT2AReceptor Agonists. J. Med. Chem. *65*, 12031–12043. https://doi.org/10.1021/acs.jmedchem.2c00702.
34. Wall, M.J., Hill, E., Huckstepp, R., Barkan, K., Deganutti, G., Leuenberger, M., Preti, B., Winfield, I., Carvalho, S., Suchankova, A., et al. (2022). Selective activation of Gαob by an adenosine A1 receptor agonist elicits analgesia without cardiorespiratory depression. Nat. Commun. *13*, 4150. https://doi.org/10.1038/s41467-022-31652-2.
35. Faouzi, A., Wang, H., Zaidi, S.A., DiBerto, J.F., Che, T., Qu, Q., Robertson, M.J., Madasu, M.K., El Daibani, A., Varga, B.R., et al. (2023). Structure-based design of bitopic ligands for the μ-opioid receptor. Nature *613*, 767–774. https://doi.org/10.1038/s41586-022-05588-y.
36. Hurtley, S.M. (2018). Continuing the resolution revolution. Science *360*, 280.11–280282. https://doi.org/10.1126/science.360.6386.280-k.
37. Provasi, D., Artacho, M.C., Negri, A., Mobarec, J.C., and Filizola, M. (2011). Ligand-Induced modulation of the Free-Energy landscape of G

protein-coupled receptors explored by adaptive biasing techniques. PLOS Comput. Biol. *7*, e1002193. https://doi.org/10.1371/journal.pcbi.1002193.

38. Provasi, D., and Filizola, M. (2010). Putative active states of a prototypic g-protein-coupled receptor from biased molecular dynamics. Biophys. J. *98*, 2347–2355. https://doi.org/10.1016/j.bpj.2010.01.047.

39. Fanelli, F., Felline, A., Raimondi, F., and Seeber, M. (2016). Structure network analysis to gain insights into GPCR function. Biochem. Soc. Trans. *44*, 613–618. https://doi.org/10.1042/BST20150283.

40. Kohlhoff, K.J., Shukla, D., Lawrenz, M., Bowman, G.R., Konerding, D.E., Belov, D., Altman, R.B., and Pande, V.S. (2014). Cloud-based simulations on Google Exacycle reveal ligand modulation of GPCR activation pathways. Nat. Chem. *6*, 15–21. https://doi.org/10.1038/nchem.1821.

41. Sun, X., Singh, S., Blumer, K.J., and Bowman, G.R. (2018). Simulation of spontaneous G protein activation reveals a new intermediate driving GDP unbinding. eLife *7*, 1–24. https://doi.org/10.7554/eLife.38465.

42. Li, J., Jonsson, A.L., Beuming, T., Shelley, J.C., and Voth, G.A. (2013). Ligand-dependent activation and deactivation of the human adenosine A 2A receptor. J. Am. Chem. Soc. *135*, 8749–8759. https://doi.org/10.1021/ja404391q.

43. Mafi, A., Kim, S.-K., and Goddard, W.A., III. (2022). The mechanism for ligand activation of the GPCR- protein complex. Proc. Natl. Acad. Sci. USA *119*. https://doi.org/10.1073/pnas.2110085119.

44. Wang, J., Bhattarai, A., Do, H.N., Akhter, S., and Miao, Y. (2022). Molecular Simulations and Drug Discovery of Adenosine Receptors. Molecules *27*, 2054. https://doi.org/10.3390/molecules27072054.

45. Saleh, N., Saladino, G., Gervasio, F.L., and Clark, T. (2017). Investigating allosteric effects on the functional dynamics of β2-adrenergic ternary complexes with enhanced-sampling simulations. Chem. Sci. *8*, 4019–4026. https://doi.org/10.1039/c6sc04647a.

46. Mafi, A., Kim, S.K., and Goddard, W.A. (2023). The dynamics of agonist-β2-adrenergic receptor activation induced by binding of GDP-bound Gs protein. Nat. Chem. *15*, 1127–1137. https://doi.org/10.1038/s41557-023-01238-6.

47. Lu, S., He, X., Yang, Z., Chai, Z., Zhou, S., Wang, J., Rehman, A.U., Ni, D., Pu, J., Sun, J., and Zhang, J. (2021). Activation pathway of a G protein-coupled receptor uncovers conformational intermediates as targets for allosteric drug design. Nat. Commun. *12*, 4721. https://doi.org/10.1038/s41467-021-25020-9.

48. Souza, P.C.T., Limongelli, V., Wu, S., Marrink, S.J., and Monticelli, L. (2021). Perspectives on High-Throughput Ligand/Protein Docking With Martini MD Simulations. Front. Mol. Biosci. *8*, 657222. https://doi.org/10.3389/fmolb.2021.657222.

49. Suomivuori, C.M., Latorraca, N.R., Wingler, L.M., Eismann, S., King, M.C., Kleinhenz, A.L.W., Skiba, M.A., Staus, D.P., Kruse, A.C., Lefkowitz, R.J., and Dror, R.O. (2020). Molecular mechanism of biased signaling in a prototypical G protein-coupled receptor. Science *367*, 881–887. https://doi.org/10.1126/science.aaz0326.

50. Dror, R.O., Arlow, D.H., Maragakis, P., Mildorf, T.J., Pan, A.C., Xu, H., Borhani, D.W., and Shaw, D.E. (2011). Activation mechanism of the β 2-adrenergic receptor. Proc. Natl. Acad. Sci. USA *108*, 18684–18689. https://doi.org/10.1073/pnas.1110499108.

51. Latorraca, N.R., Venkatakrishnan, A.J., and Dror, R.O. (2017). GPCR dynamics: Structures in motion. Chem. Rev. *117*, 139–155. https://doi.org/10.1021/acs.chemrev.6b00177.

52. Hollingsworth, S.A., and Dror, R.O. (2018). Molecular Dynamics Simulation for All. Neuron *99*, 1129–1143. https://doi.org/10.1016/j.neuron.2018.08.011.

53. Burg, J.S., Ingram, J.R., Venkatakrishnan, A.J., Jude, K.M., Dukkipati, A., Feinberg, E.N., Angelini, A., Waghray, D., Dror, R.O., Ploegh, H.L., et al. (2015). Structural biology. Structural basis for chemokine recognition and activation of a viral G protein-coupled receptor. Science *347*, 1113–1117. https://doi.org/10.1126/science.aaa5026.

54. Pan, A.C., Borhani, D.W., Dror, R.O., and Shaw, D.E. (2013). Molecular determinants of drug-receptor binding kinetics. Drug Discov. Today *18*, 667–673. https://doi.org/10.1016/j.drudis.2013.02.007.

55. Provasi, D., Bortolato, A., and Filizola, M. (2009). Exploring molecular mechanisms of ligand recognition by opioid receptors with metadynamics. Biochemistry *48*, 10020–10029. https://doi.org/10.1021/bi901494n.

56. Do, H.N., Wang, J., and Miao, Y. (2023). Deep Learning Dynamic Allostery of G-Protein-Coupled Receptors. JACS Au *3*, 3165–3180. https://doi.org/10.1021/jacsau.3c00503.

57. Sun, Z., Wang, Z., Wang, T., Wang, J., Zhang, H., Li, Z., Wang, S., Sheng, F., Yu, J., and Hou, Y. (2022). Biodegradable MnO-Based Nanoparticles with Engineering Surface for Tumor Therapy: Simultaneous Fenton-Like Ion Delivery and Immune Activation. ACS Nano *16*, 11862–11875. https://doi.org/10.1021/acsnano.2c00969.

58. Maria-Solano, M.A., and Choi, S. (2023). Dynamic allosteric networks drive adenosine A1 receptor activation and G-protein coupling. eLife *12*, 1–28. https://doi.org/10.7554/eLife.90773.

59. Di Marino, D., Conflitti, P., Motta, S., and Limongelli, V. (2023). Structural basis of dimerization of chemokine receptors CCR5 and CXCR4. Nat. Commun. *14*, 6439. https://doi.org/10.1038/s41467-023-42082-z.

60. Poucher, S.M., Keddie, J.R., Singh, P., Stoggall, S.M., Caulkett, P.W.R., Jones, G., and Coll, M.G. (1995). The in vitro pharmacology of ZM 241385,a potent,non-xanthine, A2a selective adenosine receptor antagonist. Br. J. Pharmacol. *115*, 1096–1102. https://doi.org/10.1111/j.1476-5381.1995.tb15923.x.

61. Uustare, A., Vonk, A., Terasmaa, A., Fuxe, K., and Rinken, A. (2005). Kinetic and functional properties of [3H]ZM241385, a high affinity antagonist for adenosine A2A receptors. Life Sci. *76*, 1513–1526. https://doi.org/10.1016/j.lfs.2004.10.027.

62. Pugliese, A.M., Traini, C., Cipriani, S., Gianfriddo, M., Mello, T., Giovannini, M.G., Galli, A., and Pedata, F. (2009). The adenosine A 2A receptor antagonist ZM241385 enhances neuronal survival after oxygen-glucose deprivation in rat CA1 hippocampal slices. Br. J. Pharmacol. *157*, 818–830. https://doi.org/10.1111/j.1476-5381.2009.00218.x.

63. Huang, S.K., Almurad, O., Pejana, R.J., Morrison, Z.A., Pandey, A., Picard, L.P., Nitz, M., Sljoka, A., and Prosser, R.S. (2022). Allosteric modulation of the adenosine A2A receptor by cholesterol. eLife *11*, 1–24. https://doi.org/10.7554/eLife.73901.

64. Ye, L., Neale, C., Sljoka, A., Lyda, B., Pichugin, D., Tsuchimura, N., Larda, S.T., Pomès, R., García, A.E., Ernst, O.P., et al. (2018). Mechanistic insights into allosteric regulation of the A2A adenosine G protein-coupled receptor by physiological cations. Nat. Commun. *9*, 1372. https://doi.org/10.1038/s41467-018-03314-9.

65. Fernandes, D.D., Neale, C., Gomes, G.W., Li, Y., Malik, A., Pandey, A., Orazietti, A.P., Wang, X., Ye, L., Scott Prosser, R., and Gradinaru, C.C. (2021). Ligand modulation of the conformational dynamics of the A2A adenosine receptor revealed by single-molecule fluorescence. Sci. Rep. *11*, 5910. https://doi.org/10.1038/s41598-021-84069-0.

66. Ferré, G., Anazia, K., Silva, L.O., Thakur, N., Ray, A.P., and Eddy, M.T. (2022). Global insights into the fine tuning of human A2AAR conformational dynamics in a ternary complex with an engineered G protein viewed by NMR. Cell Rep. *41*, 111844. https://doi.org/10.1016/j.celrep.2022.111844.

67. Ballesteros, J.A., and Weinstein, H. (1995). Integrated Methods for the Construction of Three-Dimensional Models and Computational Probing of Structure-Function Relations in G Protein-Coupled Receptors. Methods Neurosci. *25*, 366–428. https://doi.org/10.1016/S1043-9471(05)80049-7.

68. Schneider, E.H., Schnell, D., Strasser, A., Dove, S., and Seifert, R. (2010). Impact of the DRY motif and the missing “ionic lock” on constitutive activity and G-protein coupling of the human histamine H 4 receptor. J. Pharmacol. Exp. Ther. *333*, 382–392. https://doi.org/10.1124/jpet.109.163220.

69. Vogel, R., Mahalingam, M., Lüdeke, S., Huber, T., Siebert, F., and Sakmar, T.P. (2008). Functional Role of the "Ionic Lock"-An Interhelical Hydrogen-Bond Network in Family A Heptahelical Receptors. J. Mol. Biol. *380*, 648–655. https://doi.org/10.1016/j.jmb.2008.05.022.

70. Fleetwood, O., Matricon, P., Carlsson, J., and Delemotte, L. (2020). Energy Landscapes Reveal Agonist Control of G Protein-Coupled Receptor Activation via Microswitches. Biochemistry *59*, 880–891. https://doi.org/10.1021/acs.biochem.9b00842.

71. Dall'Igna, O.P., Porciúncula, L.O., Souza, D.O., Cunha, R.A., and Lara, D.R. (2003). Neuroprotection by caffeine and adenosine A 2A receptor blockade of β-amyloid neurotoxicity. Br. J. Pharmacol. *138*, 1207–1209. https://doi.org/10.1038/sj.bjp.0705185.

72. Effendi, W.I., Nagano, T., Kobayashi, K., and Nishimura, Y. (2020). Focusing on Adenosine Receptors as a Potential Targeted Therapy in Human Diseases. Cells *9*, 1–36. https://doi.org/10.3390/cells9030785.

73. de Lera Ruiz, M.D.L., Lim, Y.H., and Zheng, J. (2014). Adenosine A2 receptor as a drug discovery target. J. Med. Chem. *57*, 3623–3650. https://doi.org/10.1021/jm4011669.

74. Guerrero, A. (2018). A2A Adenosine Receptor Agonists and their Potential Therapeutic Applications. An Update. Curr. Med. Chem. *25*, 3597–3612. https://doi.org/10.2174/0929867325666180313110254.

75. Gołembiowska, K., and Dziubina, A. (2004). Striatal adenosine A2A receptor blockade increases extracellular dopamine release following L-DOPA administration in intact and dopamine-denervated rats. Neuropharmacology *47*, 414–426. https://doi.org/10.1016/j.neuropharm.2004.04.018.

76. Headrick, J.P., Ashton, K.J., Rose'Meyer, R.B., and Peart, J.N. (2013). Cardiovascular adenosine receptors: Expression, actions and interactions. Pharmacol. Ther. *140*, 92–111. https://doi.org/10.1016/j.pharmthera.2013.06.002.

77. Carpenter, B., Nehmé, R., Warne, T., Leslie, A.G.W., and Tate, C.G. (2016). Structure of the adenosine A2A receptor bound to an engineered G protein. Nature *536*, 104–107. https://doi.org/10.1038/nature18966.

78. García-Nafría, J., Lee, Y., Bai, X., Carpenter, B., and Tate, C.G. (2018). Cryo-EM structure of the adenosine A2A receptor coupled to an engineered heterotrimeric G protein. eLife *7*, 1–19. https://doi.org/10.7554/eLife.35946.

79. Jaakola, V.-P., Griffith, M.T., Hanson, M.A., Cherezov, V., Chien, E.Y.T., Lane, J.R., IJzerman, A.P., and Stevens, R.C. (2008). The 2.6 Angstrom Crystal Structure of a Human A_{2A} Adenosine Receptor Bound to an Antagonist. Science *322*, 1211–1217. https://doi.org/10.1126/science.1164772.

80. Segala, E., Guo, D., Cheng, R.K.Y., Bortolato, A., Deflorian, F., Doré, A.S., Errey, J.C., Heitman, L.H., IJzerman, A.P., Marshall, F.H., et al. (2016). Controlling the Dissociation of Ligands from the Adenosine A2A Receptor through Modulation of Salt Bridge Strength. J. Med. Chem. *59*, 6470–6479. https://doi.org/10.1021/acs.jmedchem.6b00653.

81. Lebon, G., Warne, T., Edwards, P.C., Bennett, K., Langmead, C.J., Leslie, A.G.W., and Tate, C.G. (2011). Agonist-bound adenosine A2A receptor structures reveal common features of GPCR activation. Nature *474*, 521–525. https://doi.org/10.1038/nature10136.

82. White, K.L., Eddy, M.T., Gao, Z.G., Han, G.W., Lian, T., Deary, A., Patel, N., Jacobson, K.A., Katritch, V., and Stevens, R.C. (2018). Structural Connection between Activation Microswitch and Allosteric Sodium Site in GPCR Signaling. Structure *26*, 259–269.e5. https://doi.org/10.1016/j.str.2017.12.013.

83. Lebon, G., Edwards, P.C., Leslie, A.G.W., and Tate, C.G. (2015). Molecular determinants of CGS21680 binding to the human adenosine A2A receptor. Mol. Pharmacol. *87*, 907–915. https://doi.org/10.1124/mol.114.097360.

84. Cui, M., Zhou, Q., Xu, Y., Weng, Y., Yao, D., Zhao, S., and Song, G. (2022). Crystal structure of a constitutive active mutant of adenosine A2A receptor. IUCrJ *9*, 333–341. https://doi.org/10.1107/S2052252522001907.

85. Park, J.H., Scheerer, P., Hofmann, K.P., Choe, H.W., and Ernst, O.P. (2008). Crystal structure of the ligand-free G-protein-coupled receptor opsin. Nature *454*, 183–187. https://doi.org/10.1038/nature07063.

86. Scheerer, P., Park, J.H., Hildebrand, P.W., Kim, Y.J., Krauss, N., Choe, H.W., Hofmann, K.P., and Ernst, O.P. (2008). Crystal structure of opsin in its G-protein-interacting conformation. Nature *455*, 497–502. https://doi.org/10.1038/nature07330.

87. Ciancetta, A., Rubio, P., Lieberman, D.I., and Jacobson, K.A. (2019). A3 adenosine receptor activation mechanisms: molecular dynamics analysis of inactive, active, and fully active states. J. Comput. Aided Mol. Des. *33*, 983–996. https://doi.org/10.1007/s10822-019-00246-4.

88. Draper-Joyce, C.J., Bhola, R., Wang, J., Bhattarai, A., Nguyen, A.T.N., Cowie-Kent, I., O'Sullivan, K., Chia, L.Y., Venugopal, H., Valant, C., et al. (2021). Positive allosteric mechanisms of adenosine A1 receptor-mediated analgesia. Nature *597*, 571–576. https://doi.org/10.1038/s41586-021-03897-2.

89. Cai, H., Guo, S., Xu, Y., Sun, J., Li, J., Xia, Z., Jiang, Y., Xie, X., and Xu, H.E. (2024). Cryo-EM structures of adenosine receptor A3AR bound to selective agonists. Nat. Commun. *15*, 3252. https://doi.org/10.1038/s41467-024-47207-6.

90. Laio, A., and Parrinello, M. (2002). Escaping free-energy minima. Proc. Natl. Acad. Sci. USA *99*, 12562–12566. https://doi.org/10.1073/pnas.202427399.

91. Barducci, A., Bussi, G., and Parrinello, M. (2008). Well-tempered metadynamics: A smoothly converging and tunable free-energy method. Phys. Rev. Lett. *100*, 020603. https://doi.org/10.1103/PhysRevLett.100.020603.

92. Branduardi, D., Gervasio, F.L., and Parrinello, M. (2007). From A to B in free energy space. J. Chem. Phys. *126*, 054103. https://doi.org/10.1063/1.2432340.

93. Grazioso, G., Limongelli, V., Branduardi, D., Novellino, E., De Micheli, C., Cavalli, A., and Parrinello, M. (2012). Investigating the mechanism of substrate uptake and release in the glutamate transporter homologue Glt Ph through metadynamics simulations. J. Am. Chem. Soc. *134*, 453–463. https://doi.org/10.1021/ja208485w.

94. Berteotti, A., Cavalli, A., Branduardi, D., Gervasio, F.L., Recanatini, M., and Parrinello, M. (2009). Protein Conformational Transitions: The Closure Mechanism of a Kinase Explored by Atomistic Simulations. J. Am. Chem. Soc. *131*, 244–250. https://doi.org/10.1021/ja806846q.

95. Bonomi, M., Branduardi, D., Gervasio, F.L., and Parrinello, M. (2008). The Unfolded Ensemble and Folding Mechanism of the C-Terminal GB1-Hairpin. J. Am. Chem. Soc. *130*, 13938–13944. https://doi.org/10.1021/ja803652f.

96. Formoso, E., Limongelli, V., and Parrinello, M. (2015). Energetics and Structural Characterization of the large-scale Functional Motion of Adenylate Kinase. Sci. Rep. *5*, 8425. https://doi.org/10.1038/srep08425.

97. Limongelli, V., Bonomi, M., Marinelli, L., Gervasio, F.L., Cavalli, A., Novellino, E., and Parrinello, M. (2010). Molecular basis of cyclooxygenase enzymes (COXs) selective inhibition. Proc. Natl. Acad. Sci. USA *107*, 5411–5416. https://doi.org/10.1073/pnas.0913377107.

98. Limongelli, V., Marinelli, L., Cosconati, S., La Motta, C., Sartini, S., Mugnaini, L., Da Settimo, F., Novellino, E., and Parrinello, M. (2012). Sampling protein motion and solvent effect during ligand binding. Proc. Natl. Acad. Sci. USA *109*, 1467–1472. https://doi.org/10.1073/pnas.1112181108.

99. Zantza, I., Pyrris, Y., Raniolo, S., Papadaki, G.F., Lambrinidis, G., Limongelli, V., Diallinas, G., and Mikros, E. (2023). Uracil/H+ Symport by FurE Refines Aspects of the Rocking-bundle Mechanism of APC-type Transporters. J. Mol. Biol. *435*, 168226. https://doi.org/10.1016/j.jmb.2023.168226.

100. Bonomi, M., Barducci, A., and Parrinello, M. (2009). Reconstructing the equilibrium boltzmann distribution from well-tempered metadynamics. J. Comput. Chem. *30*, 1615–1621. https://doi.org/10.1002/jcc.21305.

101. Bi, M., Wang, X., Wang, J., Xu, J., Sun, W., Adediwura, V.A., Miao, Y., Cheng, Y., and Ye, L. (2024). Structure and function of an intermediate

GPCR-Gαβγ complex. Preprint at bioRxiv. https://doi.org/10.1101/2024.04.02.587841.

102. Burgueño, J., Blake, D.J., Benson, M.A., Tinsley, C.L., Esapa, C.T., Canela, E.I., Penela, P., Mallol, J., Mayor, F., Lluis, C., et al. (2003). The adenosine A2A receptor interacts with the actin-binding protein α-actinin. J. Biol. Chem. *278*, 37545–37552. https://doi.org/10.1074/jbc.M302809200.

103. Navarro, G., Gonzalez, A., Campanacci, S., Rivas-Santisteban, R., Reyes-Resina, I., Casajuana-Martin, N., Cordomí, A., Pardo, L., and Franco, R. (2020). Experimental and computational analysis of biased agonism on full-length and a C-terminally truncated adenosine A2A receptor. Comput. Struct. Biotechnol. J. *18*, 2723–2732. https://doi.org/10.1016/j.csbj.2020.09.028.

104. Mundell, S., and Kelly, E. (2011). Adenosine receptor desensitization and trafficking. Biochim. Biophys. Acta *1808*, 1319–1328. https://doi.org/10.1016/j.bbamem.2010.06.007.

105. Wingler, L.M., Elgeti, M., Hilger, D., Latorraca, N.R., Lerch, M.T., Staus, D.P., Dror, R.O., Kobilka, B.K., Hubbell, W.L., and Lefkowitz, R.J. (2019). Angiotensin Analogs with Divergent Bias Stabilize Distinct Receptor Conformations. Cell *176*, 468–478.e11. https://doi.org/10.1016/j.cell.2018.12.005.

106. Lee, Y., Warne, T., Nehmé, R., Pandey, S., Dwivedi-Agnihotri, H., Chaturvedi, M., Edwards, P.C., García-Nafría, J., Leslie, A.G.W., Shukla, A.K., and Tate, C.G. (2020). Molecular basis of β-arrestin coupling to formoterol-bound β1-adrenoceptor. Nature *583*, 862–866. https://doi.org/10.1038/s41586-020-2419-1.

107. Zhuang, Y., Wang, Y., He, B., He, X., Zhou, X.E., Guo, S., Rao, Q., Yang, J., Liu, J., Zhou, Q., et al. (2022). Molecular recognition of morphine and fentanyl by the human μ-opioid receptor. Cell *185*, 4361–4375.e19. https://doi.org/10.1016/j.cell.2022.09.041.

108. Wei, S., Thakur, N., Ray, A.P., Jin, B., Obeng, S., McCurdy, C.R., McMahon, L.R., Gutiérrez-de-Terán, H., Eddy, M.T., and Lamichhane, R. (2022). Slow conformational dynamics of the human A2A adenosine receptor are temporally ordered. Structure *30*, 329–337.e5. https://doi.org/10.1016/j.str.2021.11.005.

109. Zezula, J., and Freissmuth, M. (2008). The A 2A-adenosine receptor: A GPCR with unique features? Br. J. Pharmacol. *153*, 184–190. https://doi.org/10.1038/sj.bjp.0707674.

110. Atif, M., Alsrhani, A., Naz, F., Ullah, M.I., Alameen, A.A.M., Imran, M., and Ejaz, H. (2022). Adenosine A2A receptor as a potential target for improving cancer immunotherapy. Mol. Biol. Rep. *49*, 10677–10687. https://doi.org/10.1007/s11033-022-07685-7.

111. Drube, J., Haider, R.S., Matthees, E.S.F., Reichel, M., Zeiner, J., Fritzwanker, S., Ziegler, C., Barz, S., Klement, L., Filor, J., et al. (2022). GPCR kinase knockout cells reveal the impact of individual GRKs on arrestin binding and GPCR regulation. Nat. Commun. *13*, 540. https://doi.org/10.1038/s41467-022-28152-8.

112. Maharana, J., Banerjee, R., Yadav, M.K., Sarma, P., and Shukla, A.K. (2022). Emerging structural insights into GPCR–β-arrestin interaction and functional outcomes. Curr. Opin. Struct. Biol. *75*, 102406. https://doi.org/10.1016/j.sbi.2022.102406.

113. Haider, R.S., Matthees, E.S.F., Drube, J., Reichel, M., Zabel, U., Inoue, A., Chevigné, A., Krasel, C., Deupi, X., and Hoffmann, C. (2022). β-arrestin1 and 2 exhibit distinct phosphorylation-dependent conformations when coupling to the same GPCR in living cells. Nat. Commun. *13*, 5638. https://doi.org/10.1038/s41467-022-33307-8.

114. Xu, F., Wu, H., Katritch, V., Han, G.W., Jacobson, K.A., Gao, Z.G., Cherezov, V., and Stevens, R.C. (2011). Structure of an agonist-bound human A2A adenosine receptor. Science *332*, 322–327. https://doi.org/10.1126/science.1202793.

115. Manglik, A., Kim, T.H., Masureel, M., Altenbach, C., Yang, Z., Hilger, D., Lerch, M.T., Kobilka, T.S., Thian, F.S., Hubbell, W.L., et al. (2015). Structural insights into the dynamic process of β2-adrenergic receptor signaling. Cell *161*, 1101–1111. https://doi.org/10.1016/j.cell.2015.04.043.

116. Sastry, G.M., Adzhigirey, M., Day, T., Annabhimoju, R., and Sherman, W. (2013). Protein and ligand preparation: Parameters, protocols, and influence on virtual screening enrichments. J. Comput. Aided Mol. Des. *27*, 221–234. https://doi.org/10.1007/s10822-013-9644-8.

117. Day, T.J.F., Honig, B., and Shaw, D.E. (2004). A hierarchical approach to all-atom protein loop prediction - Jacobson - 2004 - Proteins: Structure, Function, and Bioinformatics. Proteins Struct. *55*, 351–367.

118. Jacobson, M.P., Friesner, R.A., Xiang, Z., and Honig, B. (2002). On the role of the crystal environment in determining protein side-chain conformations. J. Mol. Biol. *320*, 597–608. https://doi.org/10.1016/S0022-2836(02)00470-9.

119. Greenwood, J.R., Calkins, D., Sullivan, A.P., and Shelley, J.C. (2010). Towards the comprehensive, rapid, and accurate prediction of the favorable tautomeric states of drug-like molecules in aqueous solution. J. Comput. Aided Mol. Des. *24*, 591–604. https://doi.org/10.1007/s10822-010-9349-1.

120. Shelley, J.C., Cholleti, A., Frye, L.L., Greenwood, J.R., Timlin, M.R., and Uchimaya, M. (2007). Epik: A software program for pKa prediction and protonation state generation for drug-like molecules. J. Comput. Aided Mol. Des. *21*, 681–691. https://doi.org/10.1007/s10822-007-9133-z.

121. Maier, J.A., Martinez, C., Kasavajhala, K., Wickstrom, L., Hauser, K.E., and Simmerling, C. (2015). ff14SB: Improving the Accuracy of Protein Side Chain and Backbone Parameters from ff99SB. J. Chem. Theory Comput. *11*, 3696–3713. https://doi.org/10.1021/acs.jctc.5b00255.

122. Zgarbov, M., Otyepka, M., Rí Sponer, J., St Ml, A., Ban, P., Cheatham, T.E., and Jure, P. (2011). Refinement of the Cornell et al. Nucleic Acids Force Field Based on Reference Quantum Chemical Calculations of Glycosidic Torsion Profiles. J. Chem. Theory Comput *7*, 56. https://doi.org/10.1021/ct200162x.

123. Wang, J., Wolf, R.M., Caldwell, J.W., Kollman, P.A., and Case, D.A. (2004). Development and testing of a general Amber force field. J. Comput. Chem. *25*, 1157–1174. https://doi.org/10.1002/jcc.20035.

124. Bayly, C.I., Cieplak, P., Cornell, W.D., and Kollman, P.A. (1993). A well-behaved electrostatic potential based method using charge restraints for deriving atomic charges: The RESP model. J. Phys. Chem. *97*, 10269–10280. https://doi.org/10.1021/j100142a004.

125. Wang, J., Cieplak, P., and Kollman, P.A. (2000). How Well Does a Restrained Electrostatic Potential (RESP) Model Perform in Calculating Conformational Energies of Organic and Biological Molecules? J. Comput. Chem. *21*, 1049–1074. https://doi.org/10.1002/1096-987X(200009)21:12<1049::AID-JCC3>3.0.CO;2-F.

126. Wang, J., Wang, W., Kollman, P.A., and Case, D.A. (2006). Automatic atom type and bond type perception in molecular mechanical calculations. J. Mol. Graph. Model. *25*, 247–260. https://doi.org/10.1016/j.jmgm.2005.12.005.

127. Frisch, G.W., Schlegel, H.B., Scuseria, G.E., Robb, M.A., Cheeseman, J.R., Scalmani, G., Barone, V., Petersson, G.A., Nakatsuji, H., Li, X., et al. (2016). Gaussian 16, Rev. A.03 (Gaussian, Incorp.), p. 111.

128. Case, D.A., Cheatham, T.E., Darden, T., Gohlke, H., Luo, R., Merz, K.M., Onufriev, A., Simmerling, C., Wang, B., and Woods, R.J. (2005). The Amber biomolecular simulation programs. J. Comput. Chem. *26*, 1668–1688. https://doi.org/10.1002/jcc.20290.

129. Berendsen, H.J.C., van der Spoel, D., and van Drunen, R. (1995). GROMACS: A message-passing parallel molecular dynamics implementation. Comput. Phys. Commun. *91*, 43–56. https://doi.org/10.1016/0010-4655(95)00042-E.

130. Darden, T., York, D., and Pedersen, L. (1993). Particle mesh Ewald: An N·log(N) method for Ewald sums in large systems. J. Chem. Phys. *98*, 10089–10092. https://doi.org/10.1063/1.464397.

131. Hess, B., Bekker, H., Berendsen, H.J.C., and Fraaije, J.G.E.M. (1997). LINCS: A linear constraint solver for molecular simulations. J. Comput. Chem. *18*, 1463–1472. https://doi.org/10.1002/(SICI)1096-987X(199709)18:12<1463::AID-JCC4>3.0.CO;2-H.

132. Berendsen, H.J.C., Postma, J.P.M., Van Gunsteren, W.F., Dinola, A., and Haak, J.R. (1984). Molecular dynamics with coupling to an external bath. J. Chem. Phys. *81*, 3684–3690. https://doi.org/10.1063/1.448118.

133. Bussi, G., Donadio, D., and Parrinello, M. (2007). Canonical sampling through velocity rescaling. J. Chem. Phys. *126*, 014101. https://doi.org/10.1063/1.2408420.

134. Parrinello, M., and Rahman, A. (1981). Polymorphic transitions in single crystals: A new molecular dynamics method. J. Appl. Phys. *52*, 7182–7190. https://doi.org/10.1063/1.328693.

135. Seeber, M., Felline, A., Raimondi, F., Mariani, S., and Fanelli, F. (2015). WebPSN: a web server for high-throughput investigation of structural communication in biomacromolecules. Bioinformatics *31*, 779–781. https://doi.org/10.1093/bioinformatics/btu718.

136. Felline, A., Seeber, M., and Fanelli, F. (2020). webPSN v2.0: a webserver to infer fingerprints of structural communication in biomacromolecules. Nucleic Acids Res. *48*, W94–W103. https://doi.org/10.1093/nar/gkaa397.

137. Felline, A., Seeber, M., and Fanelli, F. (2022). PSNtools for standalone and web-based structure network analyses of conformational ensembles. Comput. Struct. Biotechnol. J. *20*, 640–649. https://doi.org/10.1016/j.csbj.2021.12.044.

138. Tiwary, P., and Parrinello, M. (2013). From metadynamics to dynamics. Phys. Rev. Lett. *111*, 230602. https://doi.org/10.1103/PhysRevLett.111.230602.

139. Kearsley, S.K. (1989). On the orthogonal transformation used for structural comparisons. Acta Crystallogr. A Found. Crystallogr. *45*, 208–210. https://doi.org/10.1107/S0108767388010128.

140. Tribello, G.A., Bonomi, M., Branduardi, D., Camilloni, C., and Bussi, G. (2014). PLUMED 2: New feathers for an old bird. Comput. Phys. Commun. *185*, 604–613. https://doi.org/10.1016/j.cpc.2013.09.018.

141. Bonomi, M., Bussi, G., Camilloni, C., Tribello, G.A., Banáš, P., Barducci, A., Bernetti, M., Bolhuis, P.G., Bottaro, S., and Branduardi, D. (2019). Promoting transparency and reproducibility in enhanced molecular simulations. Nat. Methods *16*, 670–673. https://doi.org/10.1038/s41592-019-0506-8.

142. Dominguez, C., Boelens, R., and Bonvin, A.M.J.J. (2003). HADDOCK: A Protein−Protein Docking Approach Based on Biochemical or Biophysical Information. J. Am. Chem. Soc. *125*, 1731–1737. https://doi.org/10.1021/ja026939x.

143. Van Zundert, G.C.P., Rodrigues, J.P.G.L.M., Trellet, M., Schmitz, C., Kastritis, P.L., Karaca, E., Melquiond, A.S.J., Van Dijk, M., De Vries, S.J., and Bonvin, A.M.J.J. (2016). The HADDOCK2.2 Web Server: User-Friendly Integrative Modeling of Biomolecular Complexes. J. Mol. Biol. *428*, 720–725. https://doi.org/10.1016/j.jmb.2015.09.014.

Chem, Volume *10*

Supplemental information

Minute-timescale free-energy calculations reveal a pseudo-active state in the adenosine A_{2A} receptor activation mechanism

Vincenzo Maria D'Amore, Paolo Conflitti, Luciana Marinelli, and Vittorio Limongelli

Supplemental Information

TABLE OF CONTENTS

Table S1: Conformational features of main $A_{2A}R$ states

Table S2: List of atoms selected for ${}_{ACT}P$ collective variable

Table S3: List of atoms selected for ${}_{TM6}P$ collective variable

Figure S1: RMSD plots of the $A_{2A}R$ orthosteric binding site.

Figure S2: RMSD plots of the $A_{2A}R$ intracellular binding site

Figure S3: Pearson Coefficient Matrices and Protein Structure Network

Figure S4: RMSD plots of the $A_{2A}R$ transmembrane helices

Figure S5: RMSD plots of the $A_{2A}R$ connector region

Figure S6: Stability of the pseudo-active $A_{2A}R$ in MD simulations

Figure S7: Comparison of the TM6 translational movement in different $A_{2A}R$ structures

Figure S8: Plot of the IBS's volume in the FApo system

Figure S9: Violin plots of the $H^{6.32}$-$R^{7.56}$ and $H^{6.32}$-$R^{8.48}$ cation-π interactions

Figure S10: Activating Ionic Lock (AIL) formation in rhodopsin and adenosine receptors

Figure S11: Graphical representations of the path collective variables (PCVs)

Figure S12: Convergence plots of the free-energy calculation in NECA-bound $A_{2A}R$

Figure S13: Convergence plots of the free-energy calculation in the apo $A_{2A}R$

Figure S14: Convergence plots of the free-energy calculation in the ZMA-bound $A_{2A}R$

Figure S15: RMSD plots of the energy minima receptor structures during unbiased MD simulations

Figure S16: FESs as a function of ${}_{ACT}P.s$ and ${}_{TM6}P.s$

Figure S17: Protein-Protein docking results

Figure S18: Targeted and unbiased MD calculations on $A_{2A}R$/G Protein complex

Figure S19: pAs-$A_{2A}R$/G protein binding mode

Figure S20: Structural stability of the pAs-$A_{2A}R$/β-arrestin 1 complex

Figure S21: Stability of the Activating Ionic Lock (AIL) interaction

Figure S22: Projection of $A_{2A}R$ experimental structures onto the FES of the $A_{2A}R$/NECA system

Figure S23: Comparison between ^{19}F-NMR spectra and free energy surfaces of A2AR

Table S1: Conformational microswitches in the diverse $A_{2A}R$ states

	Microswitch			
**State**	NPxxY		AIL	IIL
A	1.4	3.7	3.9	20.2
pAs	2.0	4.0	4.3	17.6
Intermediate Active	3.4	4.5	12.9	11.8
I1	3.9	1.9	16.0	4.0
I2	3.9	1.7	16.4	7.0
	RMSD (Å)[1]	RMSD (Å)[2]	Distance (Å)[3]	Distance (Å)[3]

[1] The RMSD was computed for the C-α of the transmembrane helices after with respect to $A_{2A}R$ active conformation (PDB code:5G53)

[2] The RMSD was computed for the C-α of the transmembrane helices after with respect to $A_{2A}R$ active conformation (PDB code: 3PWH)

[3] The values of AIL and IIL correspond to the distances of E6.30-Cδ from R5.66-Cζ and R3.50-Cɣ, respectively.

Table S2: List of atoms used for the alignment and RMSD measurement of $_{ACT}$Path CV (residues numbered according to UNIPROT ID P29274 numeration)

RMSD computation		Alignment	
**Atom**	**Residue**	**Atom**	**Residue**
Cα and Cβ	92	Cα	19-29
Cα and Cβ	189	Cα	44-64
Cα and Cβ	201	Cα	84-89
Cα and Cβ	205	Cα	119-138
Cα and Cβ	226-232	Cα	181-186
Cα and Cβ	234-236	Cα	246-251
Cα and Cβ	240-250	Cα	272-277
Cα and Cβ	284-289		
Cγ	208		
Cγ	225		
Cζ	202		
Cζ	288		

Table S3: List of contacts used for computing of $_{TM6}$Path CV (residues numbered according to UNIPROT ID P29274 numeration).

Contact List					
**Contact number**	**Atom i**	**Atom j**	**R_0**	***n***	***m***
1	Glu228-Cδ	Arg205-Cζ	7Å	8	16
2	Glu228-Cδ	Arg102-Cζ	7Å	8	16
3	Glu228-Cδ	Asp101-Cγ	16.5Å	8	18
4	Arg102-Cζ	Asp97-Cγ	7.5Å	6	18
5	Leu235- Cγ	Leu198-Cγ	9Å	8	16
6	Leu235- Cγ	Ile98-Cβ	10Å	10	24
7	Tyr288-Cζ	Tyr197-Cζ	8.5Å	8	28
8	Tyr288-Cζ	Val45-Cζ	6.7Å	12	24

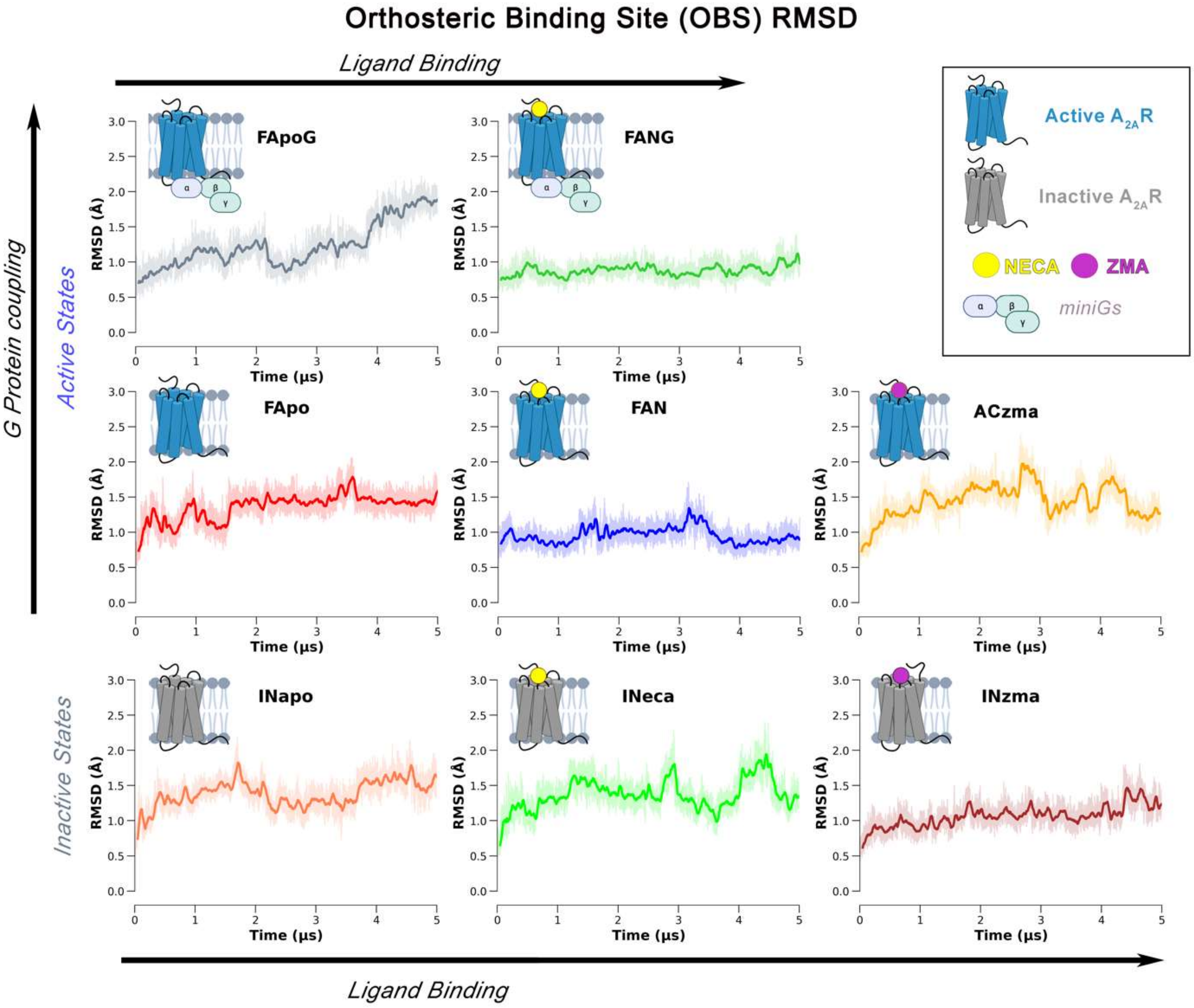

Figure S1. RMSD plots of the $A_{2A}R$ **orthosteric binding site**. The RMSD plots were computed using the Cα atoms of residues 8-14, 57-66, 75-90, 132-140, 175-187, 245-258, 267-278 (residue numbering as in UNIPROT ID P29274) with respect to the first frame of each trajectory. The bolded lines show a RMSD value smoothed with a rolling window of 5 ns, whereas the actual fluctuations are shown with a slight transparency.

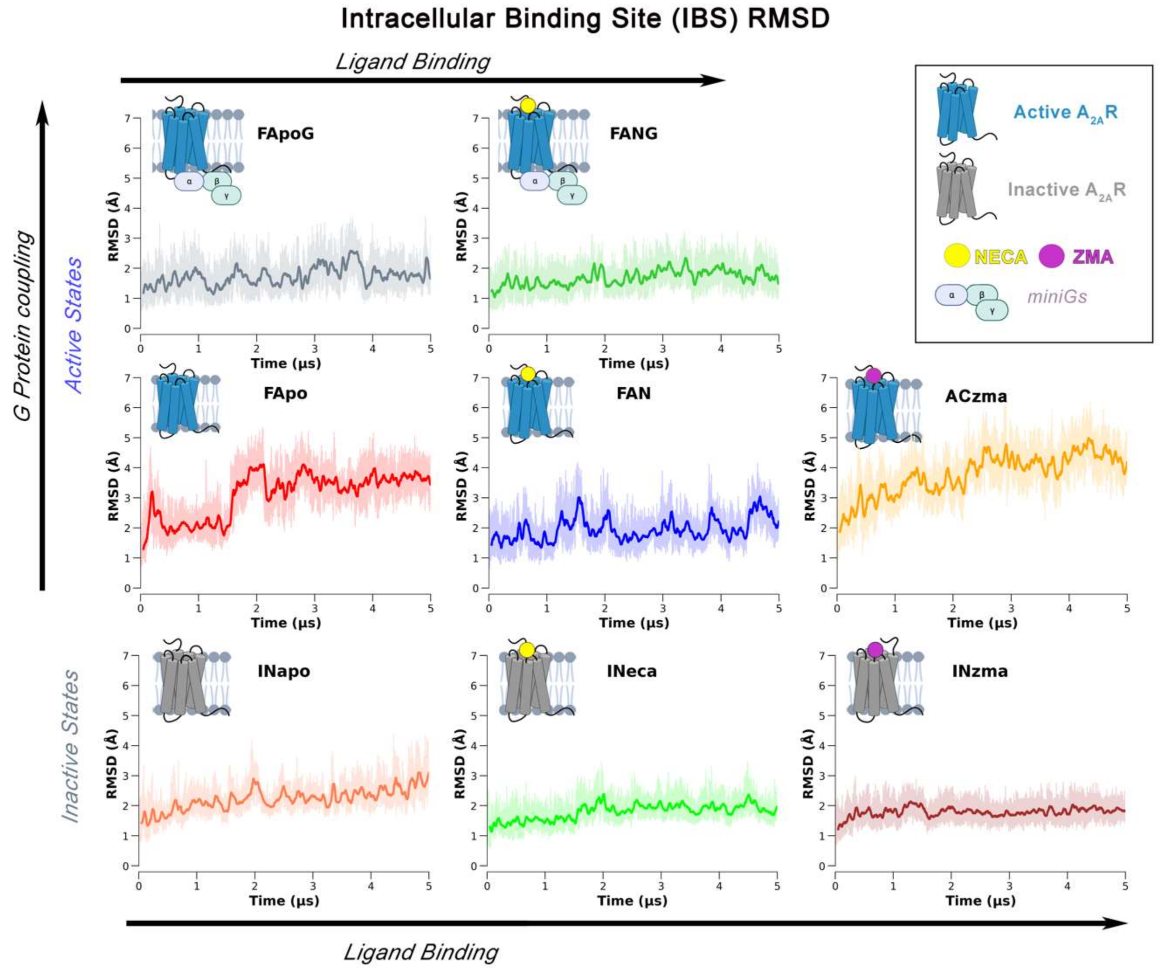

Figure S2. RMSD plots of the $A_{2A}R$ **intracellular binding site**. The RMSD plots were computed using the IBS Cα atoms of residues 24-31, 42-49, 98-106, 119-126, 198-208, 222-235, 283-287 (residue numbering as in UNIPROT ID P29274) with respect to the first frame of each trajectory. The bolded lines show a RMSD value smoothed with a rolling window of 5 ns, whereas the actual fluctuations are shown with a slight transparency.

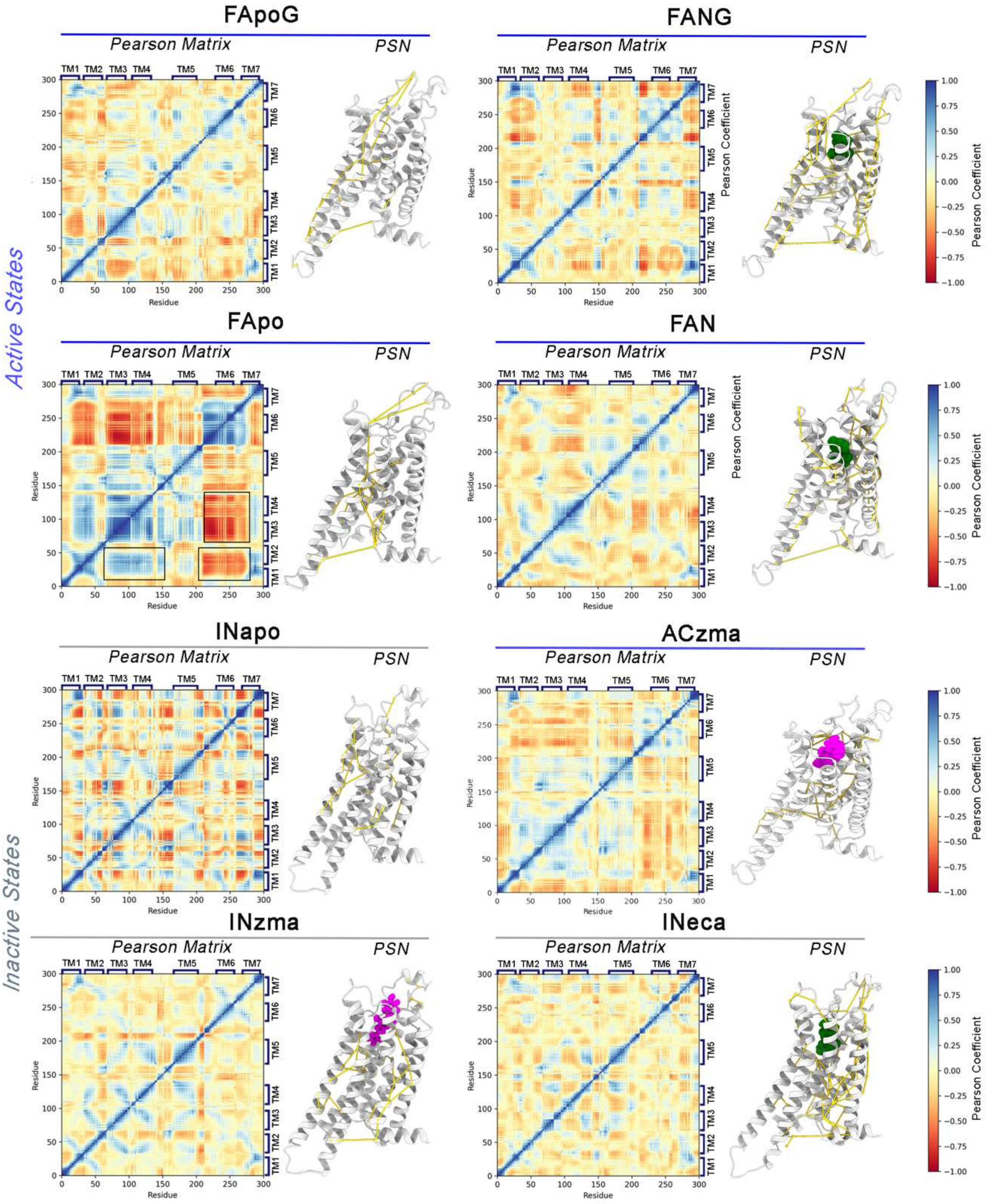

Figure S3. Pearson Coefficient Matrices and Protein Structure Network. The Pearson Coefficients were computed based on the corresponding elements of the covariance matrix of the atomic (Cα of each residue) fluctuations during the unbiased trajectories. The PSN were obtained with the aid of the webpsn server; the links connecting the most important aminoacidic hubs in the global metapath are depicted as gold connections and projected onto a cartoon representation of the starting $A_{2A}R$ conformation in the specific MD run.

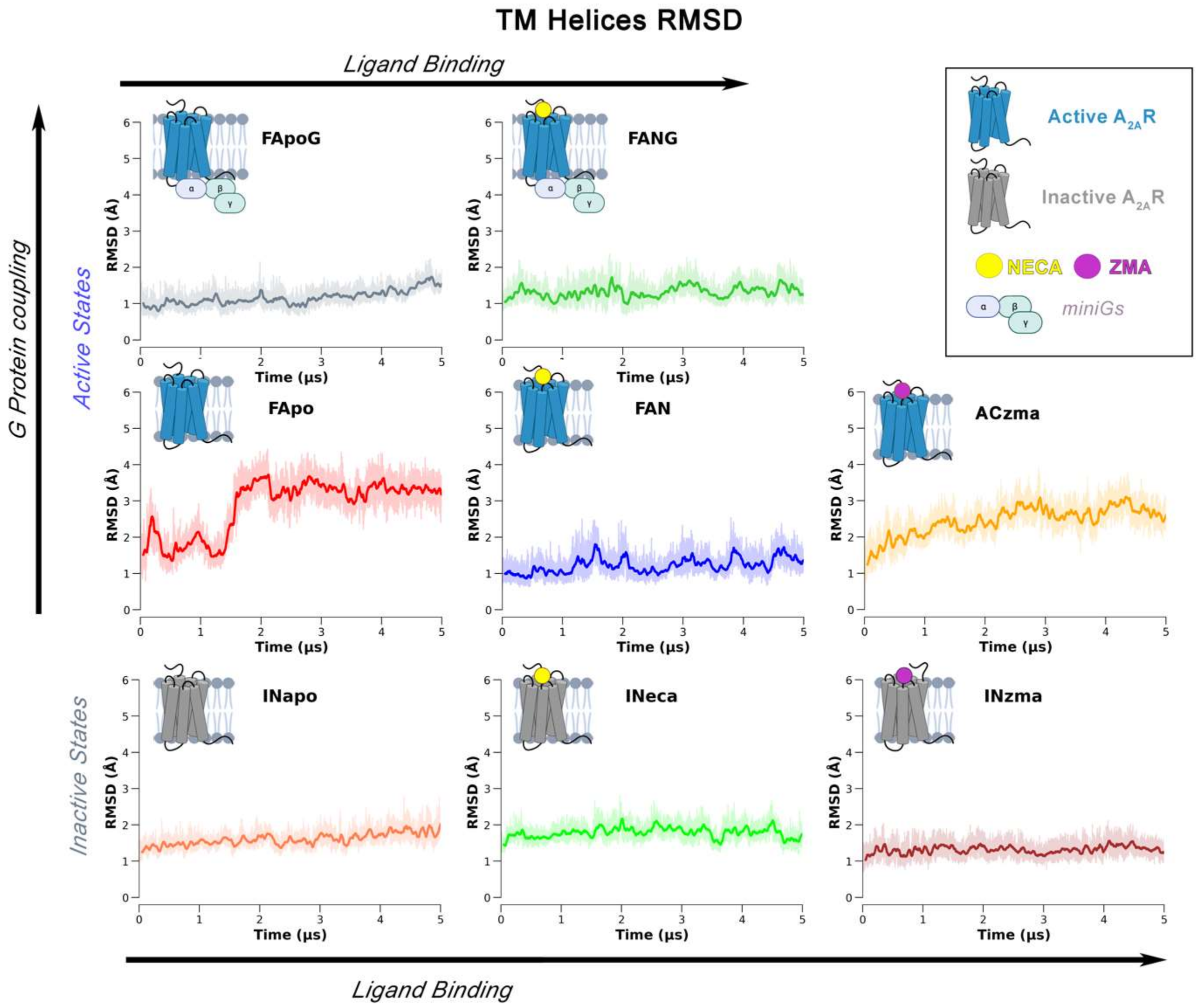

Figure S4. RMSD plots of the A2AR **transmembrane helices**. The RMSD plots were computed using the Cα atoms of residues 8-31, 42-66, 75-106, 119-140, 175-205, 227-257, 267-287 (residue numbering as in UNIPROT ID P29274) with respect to the first frame of each trajectory. The bolded lines show a RMSD value smoothed with a rolling window of 5 ns, whereas the actual fluctuations are shown with a slight transparency.

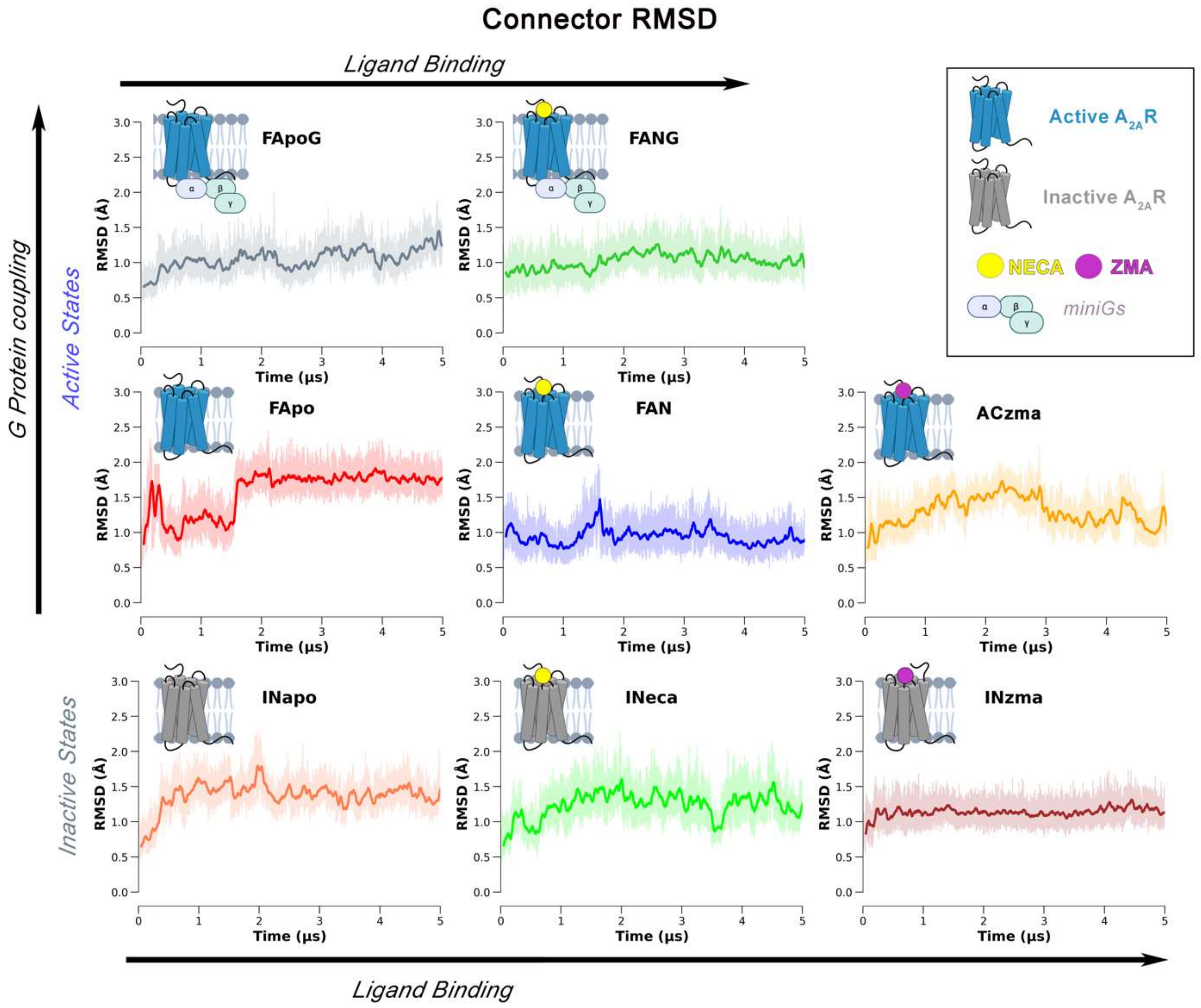

Figure S5. RMSD plots of the A2AR **connector region.** The RMSD plots were computed using the Cα atoms of residues 15-23, 50-56, 91-97, 127-131, 188-197, 236-244, 279-283 (residue numbering as in UNIPROT ID P29274) with respect to the first frame of each trajectory. The bolded lines show a RMSD value smoothed with a rolling window of 5 ns, whereas the actual fluctuations are shown with a slight transparency.

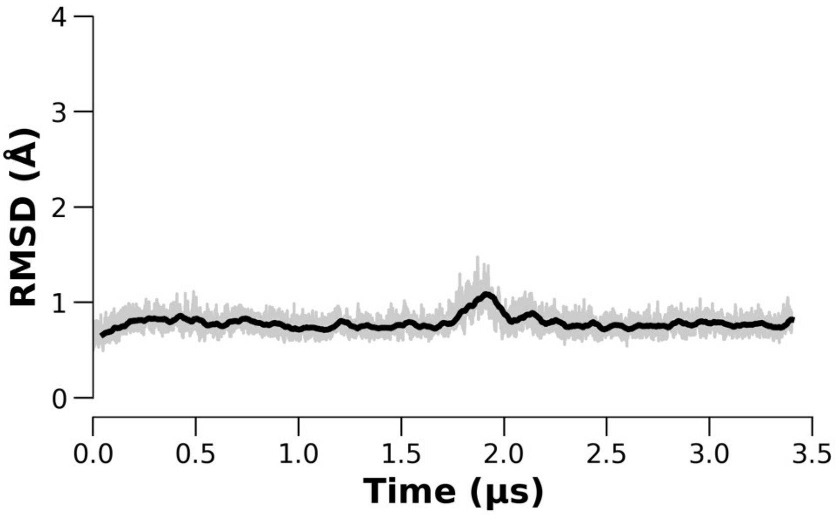

Figure S6. Stability of the pseudo-active A_{2A}R in MD simulations. RMSD plots were computed for the GPCR transmembrane helices (Cα atoms of residues 8-31, 42-66, 75-106, 119-140, 175-205, 227-

257, 267-287; residue numbering as in UNIPROT ID P29274) with respect to the first frame after the conformational transition observed in the *FApo* system. The bolded lines show a RMSD value smoothed with a rolling window of 5 ns, whereas the actual fluctuations are shown with a slight transparency.

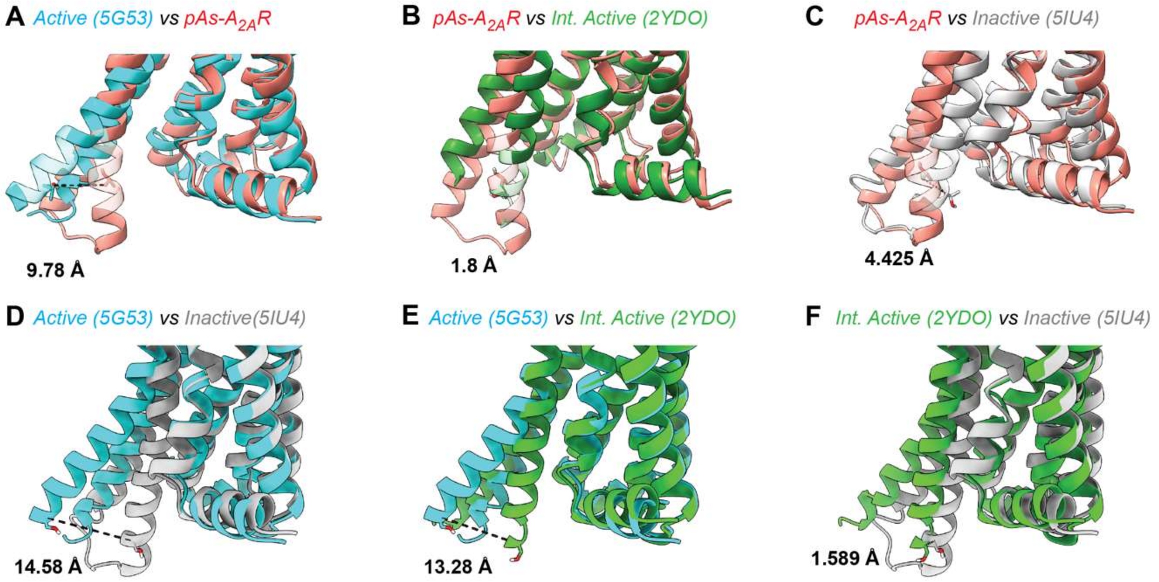

Figure S7. Comparison of the TM6 translational movement in different $A_{2A}R$ structures. Superposition between the intracellular section of pAs-$A_{2A}R$ (salmon) with the experimental active (cyan, PDB code 5G53), the intermediate-active (green, PDB code 2YDO), and the inactive structures (gray, PDB code 5IU4). The displacement of the cytoplasmic end of TM6 is measured as the distance between the Cα atoms of $Thr224^{6.26}$ in the different conformations (threonine is displayed as sticks).

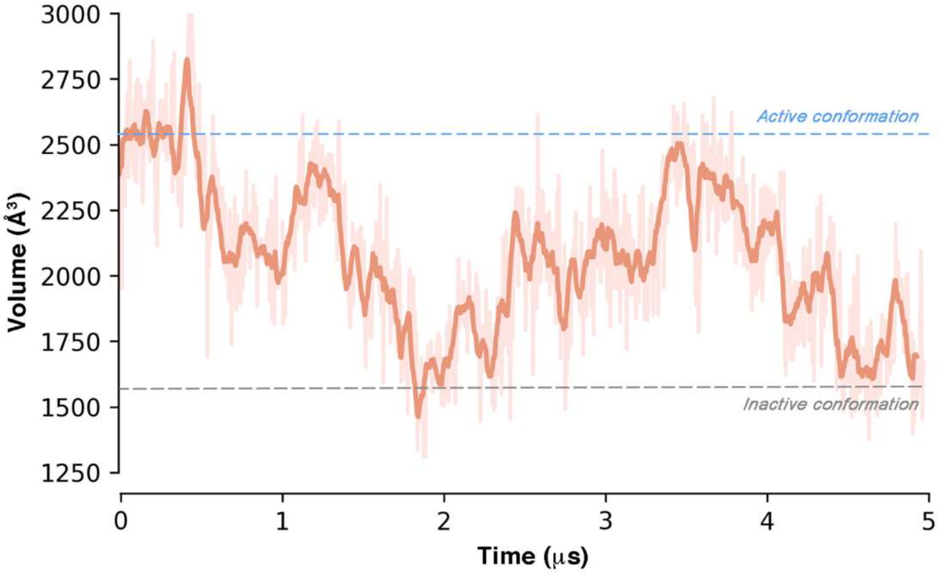

Figure S8. Plot of the IBS's volume in the FApo system. The bolded lines show a volume value smoothed with a rolling window of 5ns, whereas the actual fluctuations are shown with a slight transparency. The reference volume values for $A_{2A}R$ active and inactive states are shown as light-blue and grey dashed lines, respectively. The volume computation was performed with the aid POVME.py tool (J. Chem. Theory Comput. 2017, 13, 9, 4584–4592) by setting an inclusion sphere of 16Å centered at the center of mass of residues 24-31, 42-49, 98-106, 119-126, 198-208, 222-235, 283-287; residue numbering as in UNIPROT ID P29274.

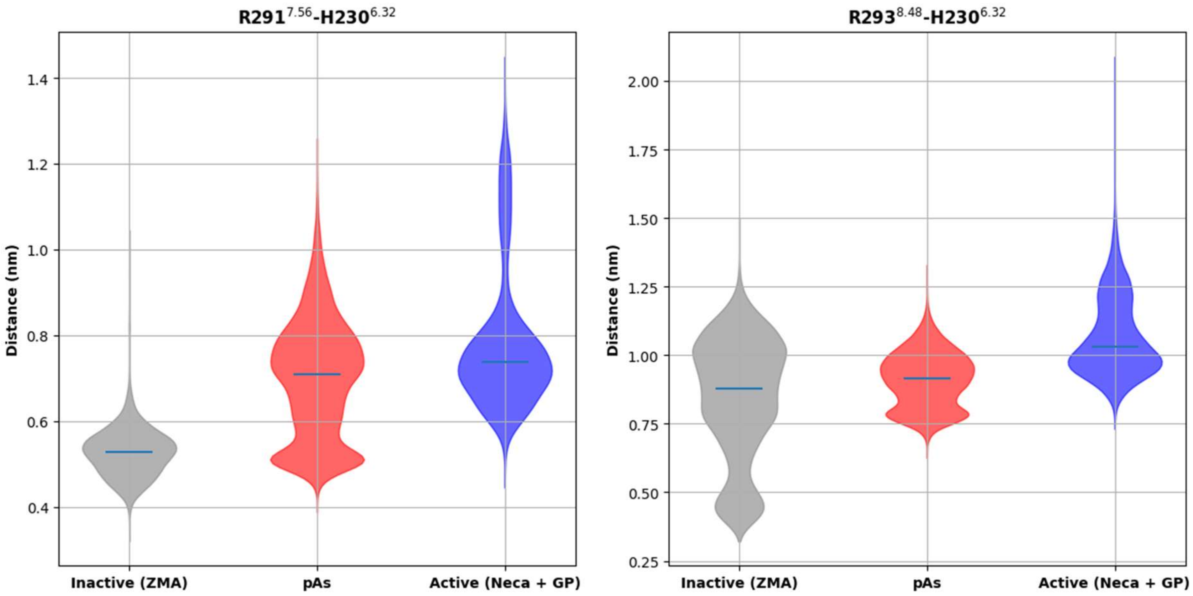

Figure S9. Violin plots of the $H^{6.32}$-$R^{7.56}$ and $H^{6.32}$-$R^{8.48}$ **cation-π interactions.** The plots are computed for the residue pairs $H^{6.32}$-$R^{7.56}$ (Left) and $H^{6.32}$-$R^{8.48}$ (Right) over the unbiased MD trajectories of $A_{2A}R$ in the inactive (in complex with ZMA), pAs, and active (in complex with NECA e G Protein) states. Distances are measured between the center of the imidazole ring of histidine and the Cζ of arginine residues.

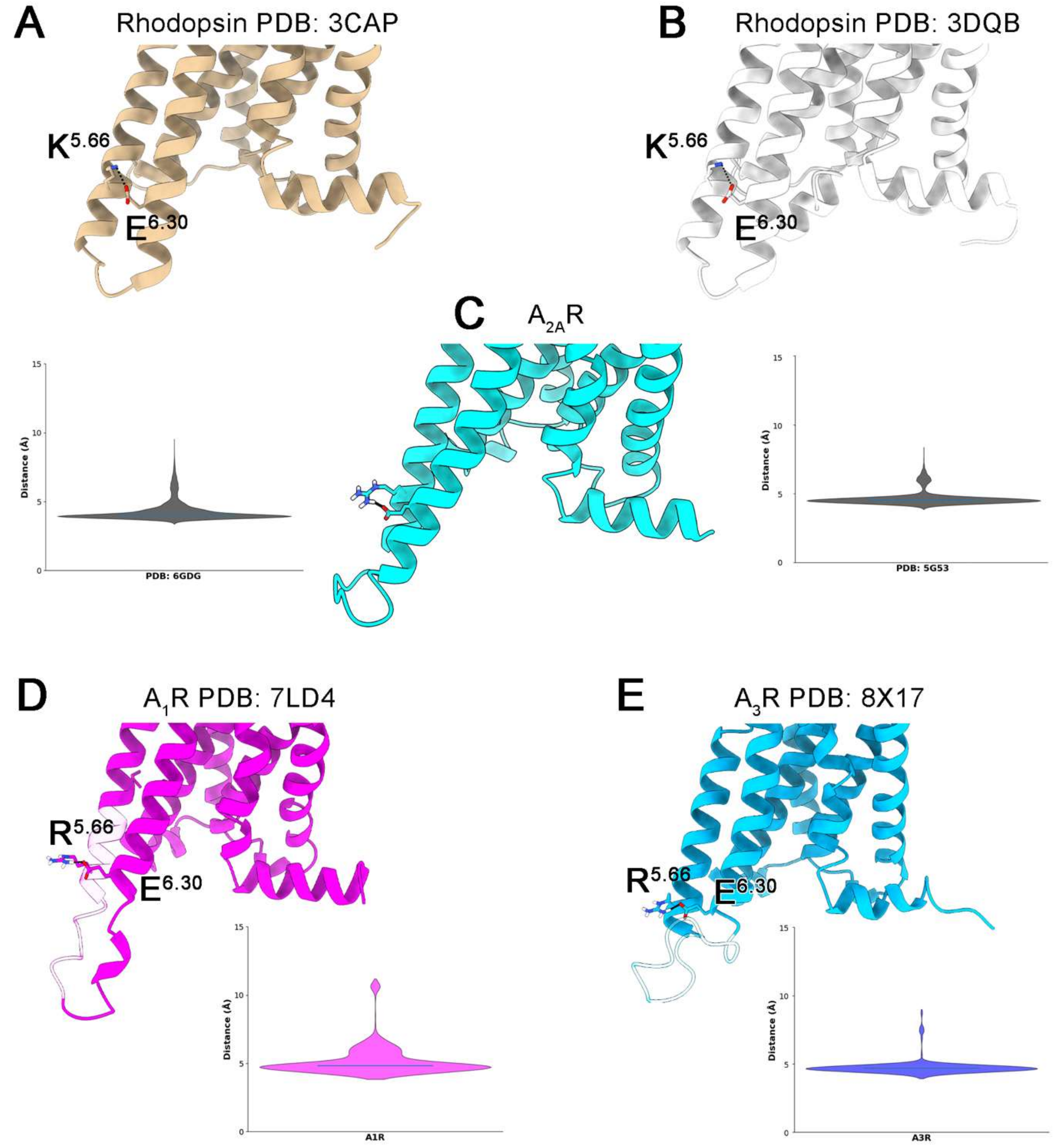

Figure S10. Activating Ionic Lock (AIL) formation in rhodopsin and adenosine receptors. Atomistic detail of the TM5-TM6 salt bridge interaction observed between K/$R^{5.66}$ and $E^{6.30}$ in the experimental active structures of rhodopsin with PDB codes 3CAP (A) and 3DQB (B), and in the $A_{2A}R$ (C), A_1R (D) and A_3R (E) receptors during MD simulations. The violin plots computed for the distance between $R^{5.66}$'s Cδ and $E^{6.30}$'s Cζ are shown as insets.

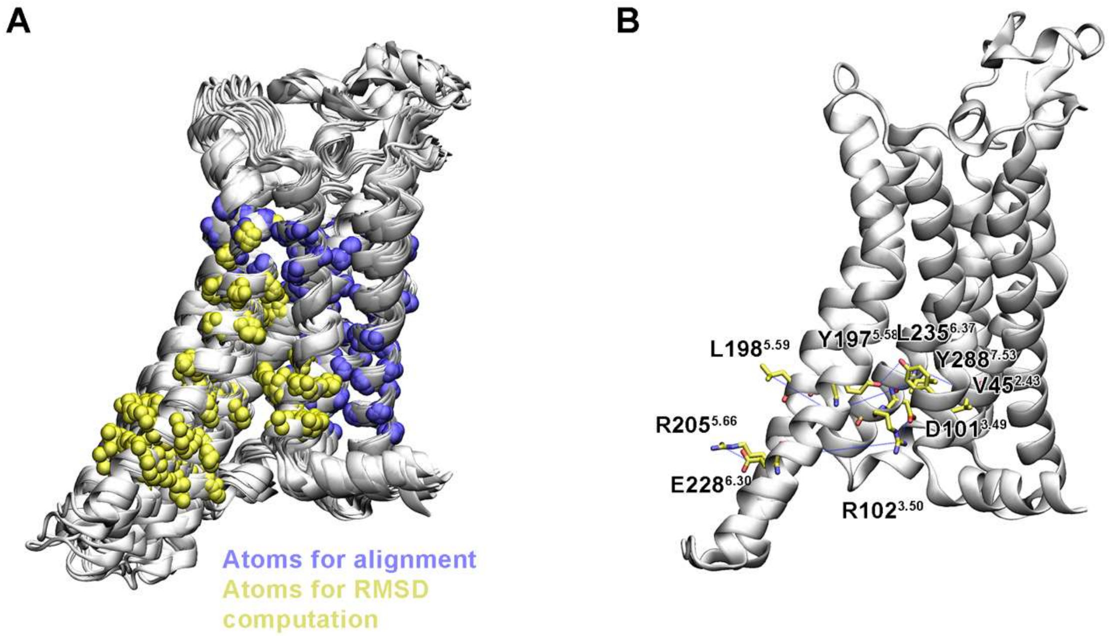

Figure S11. Graphical representations of the path collective variables (PCVs). A) Cartoon representation of the 12 frames forming the reference path used for the $_{ACT}$ Path Collective Variable. The RMSD matrix was computed for the atoms shown as yellow sphere and listed in Table S1, after alignment of the frames based on the position of the Cα and Cβ atoms depicted as blue spheres. B) Graphical representation of the seven contacts (blue dashed lines) used for defining the $_{TM6}$Path Collective variable in the CMAP space, which are listed in Table S2. For sake of clarity the contacts are projected on the first of the nine frames forming the path, corresponding to the receptor active conformation.

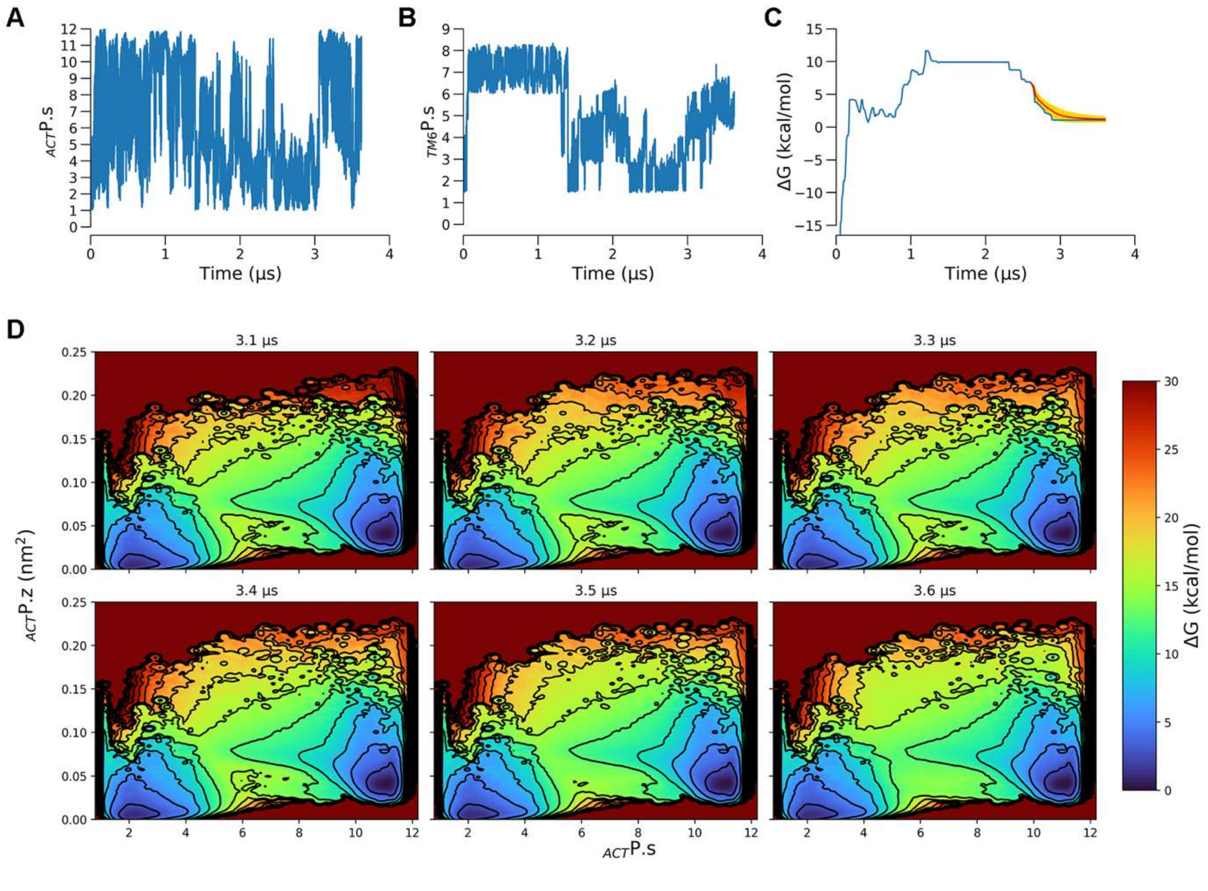

Figure S12. Convergence plots of the free-energy calculation in NECA-bound $A_{2A}R$. A) Time evolution of the $_{ACT}$P.s collective variable biased during the simulation. B) Time evolution of the $_{TM6}$P.s collective variable biased during the simulation. C) Free energy difference between the two main energy basins (A* and I*) as a function of the simulation time (blue). Its time-weighted average is reported in red, error bars are shown in yellow. D) Time evolution of the reweighted FES as a function of the $_{ACT}$P.s and $_{ACT}$P.z CVs during the last 500 ns of simulation.

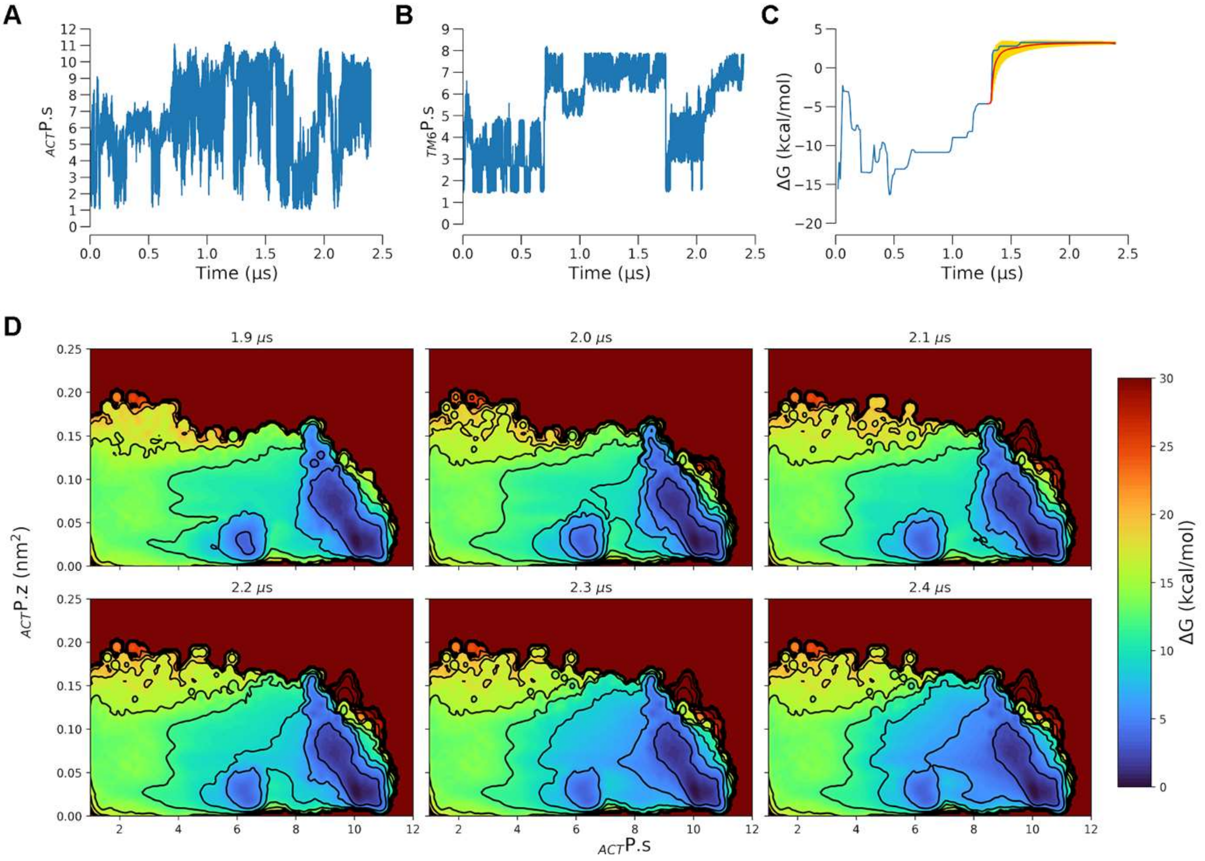

Figure S13. Convergence plots of the free-energy calculation in the apo $A_{2A}R$. A) Time evolution of the $_{ACT}$P.s collective variable biased during the simulation. B) Time evolution of the $_{TM6}$P.s collective variable biased during the simulation. C) Free energy difference between the two main energy basins (A* and I*) as a function of the simulation time (blue). Its time-weighted average is reported in red, error bars are shown in yellow. D) Time evolution of the reweighted FES as a function of the $_{ACT}$P.s and $_{ACT}$P.z CVs during the last 500 ns of simulation.

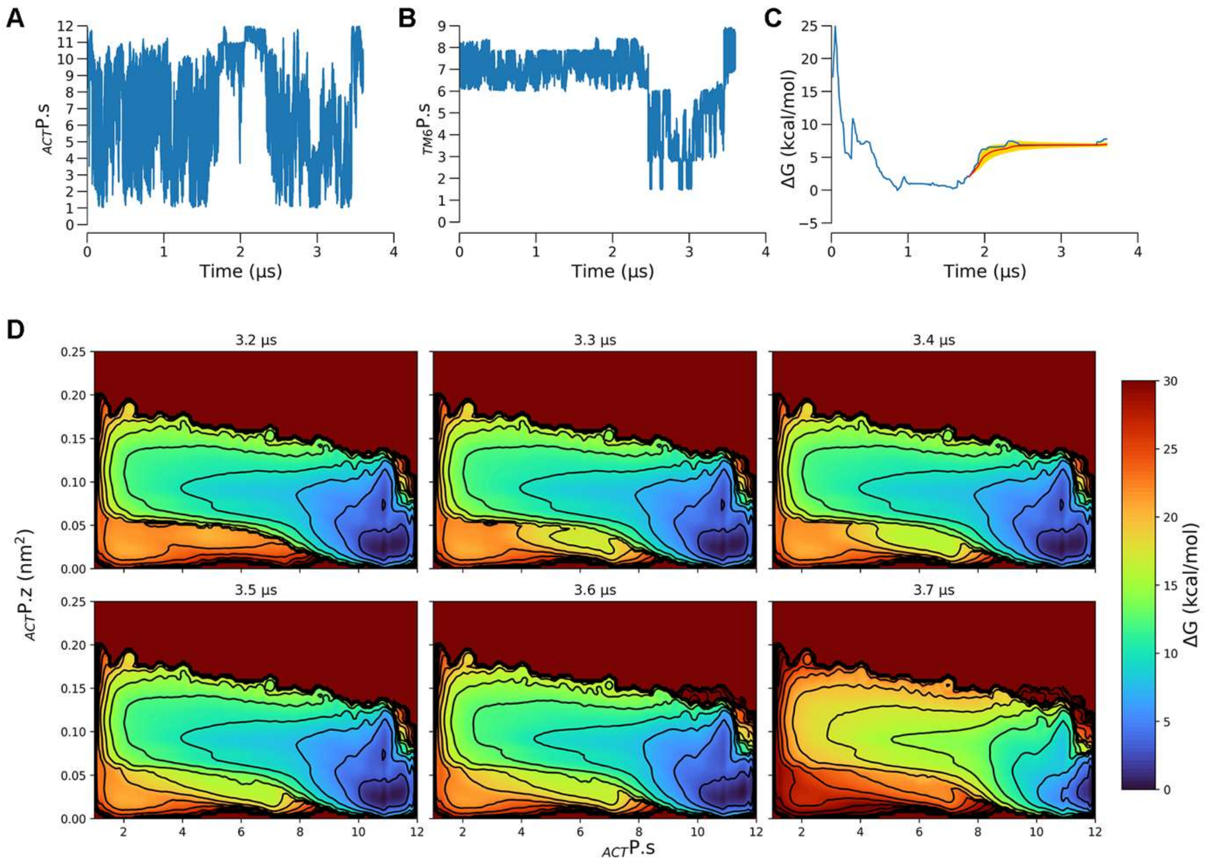

Figure S14. Convergence plots of the free-energy calculation in the ZMA-bound A$_{2A}$R. A) Time evolution of the $_{ACT}$P.s collective variable biased during the simulation. B) Time evolution of the $_{TM6}$P.s collective variable biased during the simulation. C) Free energy difference between the two main energy basins (A* and I*) as a function of the simulation time (blue). Its time-weighted average is reported in red, error bars are shown in yellow. D) Time evolution of the reweighted FES as a function of the $_{ACT}$P.s and $_{ACT}$P.z CVs during the last 500 ns of simulation.

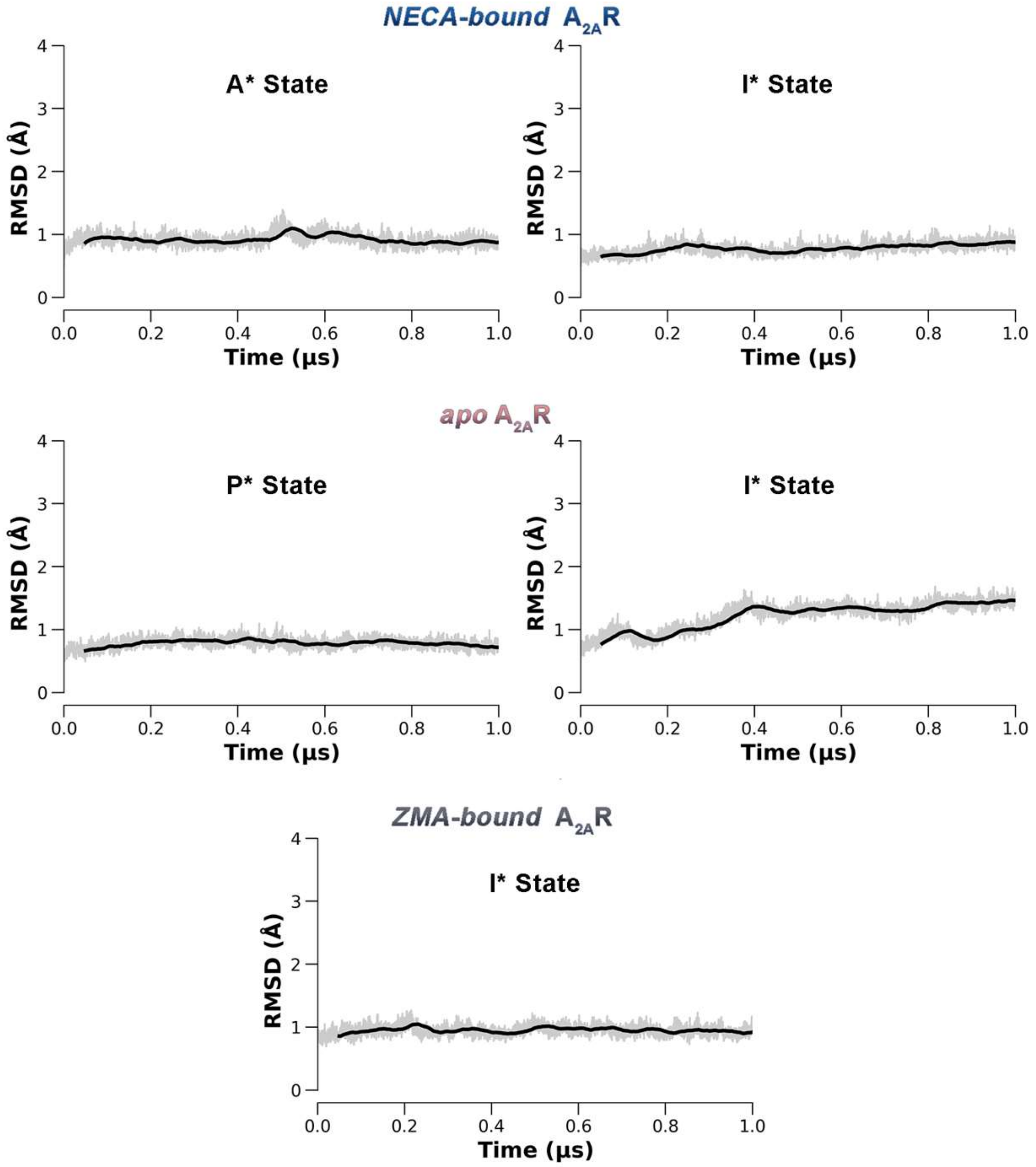

Figure S15. RMSD plots of the energy minima receptor structures during unbiased MD simulations. RMSD plots are computed for the Cα atoms of the TM helices (Cα atoms of residues 8-31, 42-66, 75-106, 119-140, 175-205, 227-257, 267-287) with respect to the starting conformation of each minimum. For the A state in the *NECA-bound* system and I state in the *ZMA-bound* system, the plots refer to the first 1 µs MD simulations performed on the X-ray structures and also described in Supplementary Fig.1-4. For the P state in the *apo* system, the plot refers to the first 1 µs after the conformational transition observed in the *FApo* MD system described in Supplementary Fig. 1-4. The bolded lines show a RMSD value smoothed with a rolling window of 5 ns, whereas the actual fluctuations are shown with a slight transparency.

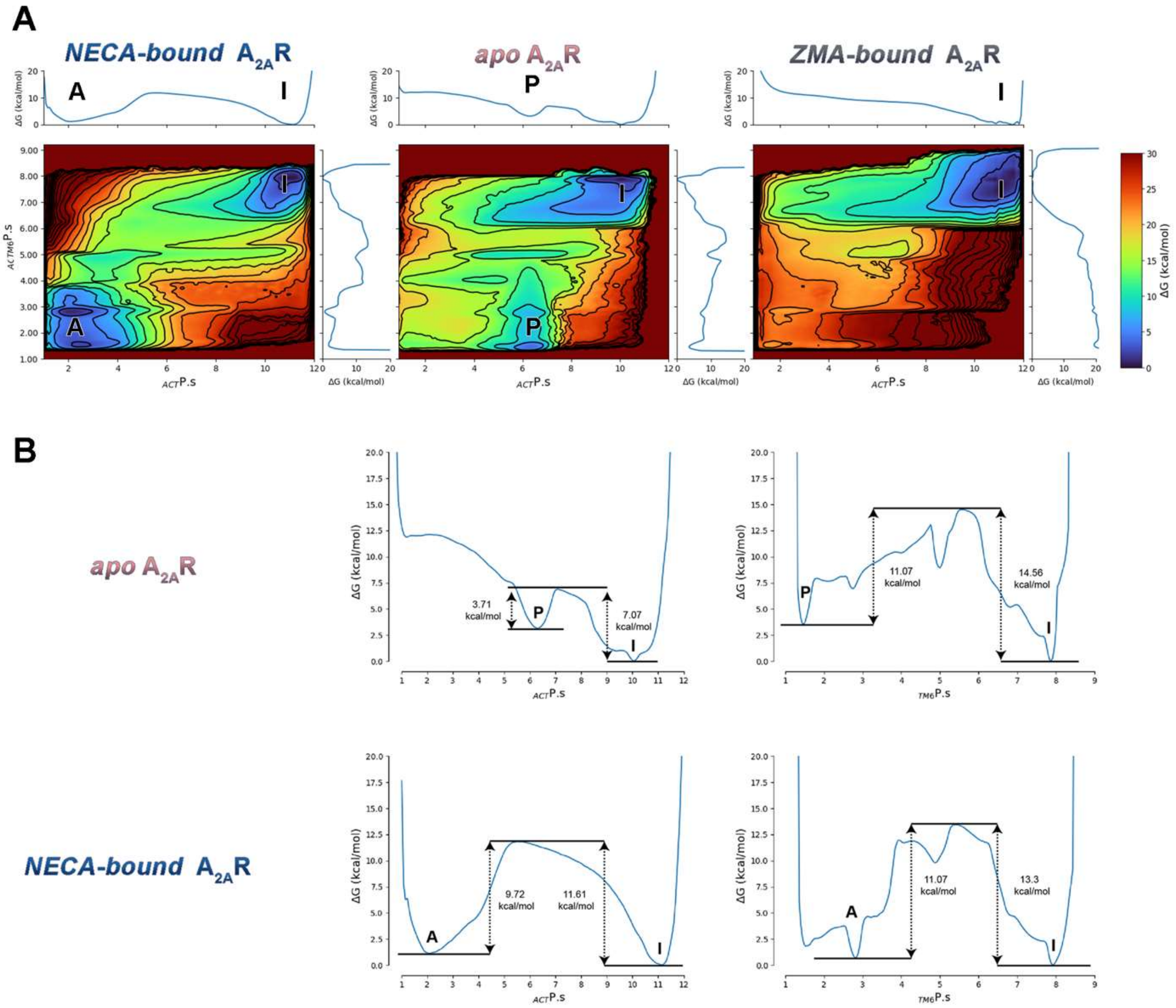

Figure S16. FESs as a function of $_{ACT}$P.s and $_{TM6}$P.s. A) Activation Free Energy landscapes of the *NECA-bound*, *apo* and *ZMA-bound* forms of A_{2A}R as a function of the $_{ACT}$P.s and $_{TM6}$P.s collective variables. Isosurfaces are displayed every 3 kcal/mol. B) 1-D free energy profiles of the apo and the NECA-bound A_{2A} calculated as a function of the $_{ACT}$P.s (left) and the $_{TM6}$P.s (right) CVs.

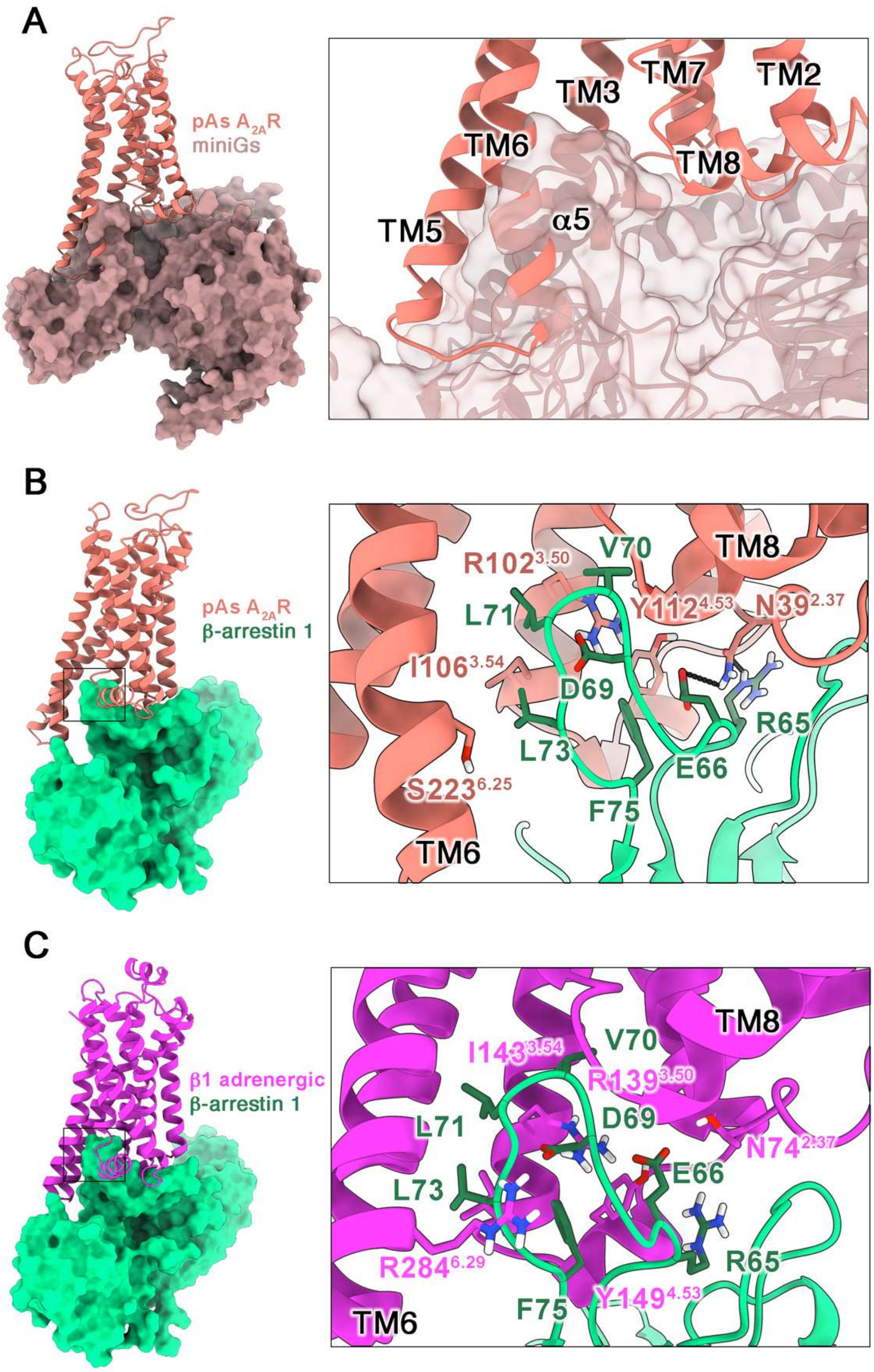

Figure S17. Protein-Protein docking results. A) Steric clash between the intracellular portion of $A_{2A}R$ and the α5 helix of Gα subunit in the the pAs/G protein complex obtained by manual fitting. B) Docking complex predicted by Haddock of the pAs-$A_{2A}R$/β-arrestin 1 interaction. The pAs $A_{2A}R$ structure is shown as salmon cartoon, whereas the mini Gs and β-arrestin 1 are depicted (cartoon and surface) in brown and green, respectively. C) Experimental Cryo-EM structure of β arrestin 1 (green surface and cartoon) at the β1 adrenoreceptor IBS (purple cartoon, PDB code: 6TKO).

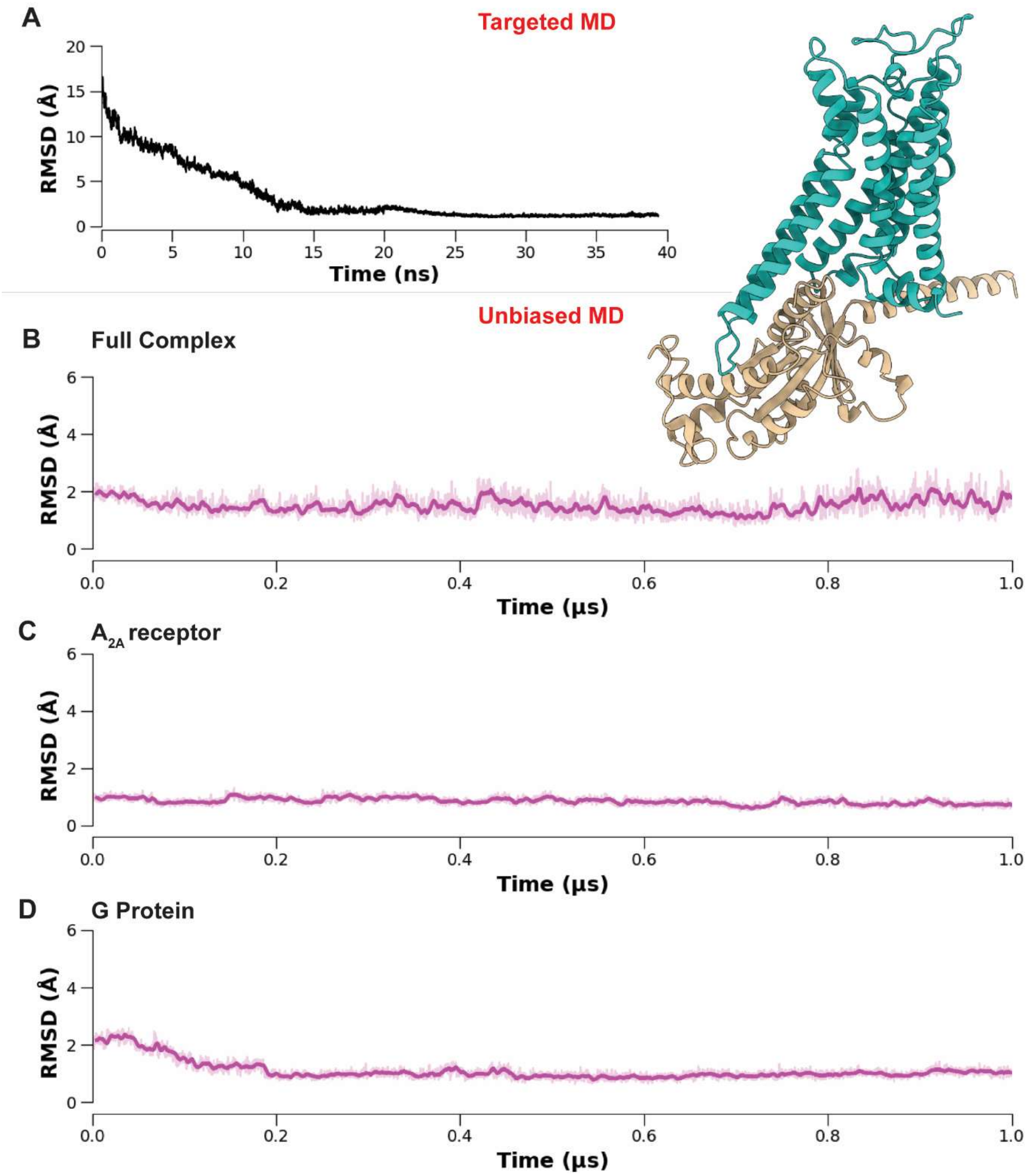

Figure S18. Targeted and unbiased MD calculations on A_{2A}R/G Protein complex. A) Evolution of the RMSD of the G protein secondary structure's Cβ calculated relative to the experimental A_{2A}R-miniGs complex (PDB: 6GDG) during 40 ns targeted MD simulations. B-D) Stability of the A_{2A}R-miniGs complex throughout 1 μs unbiased MD simulation. Evolution of the RMSD of (B) the binary complex, (C) the A_{2A}R receptor, and (D) the G protein during the unbiased MD calculated relative to the average protein conformation.

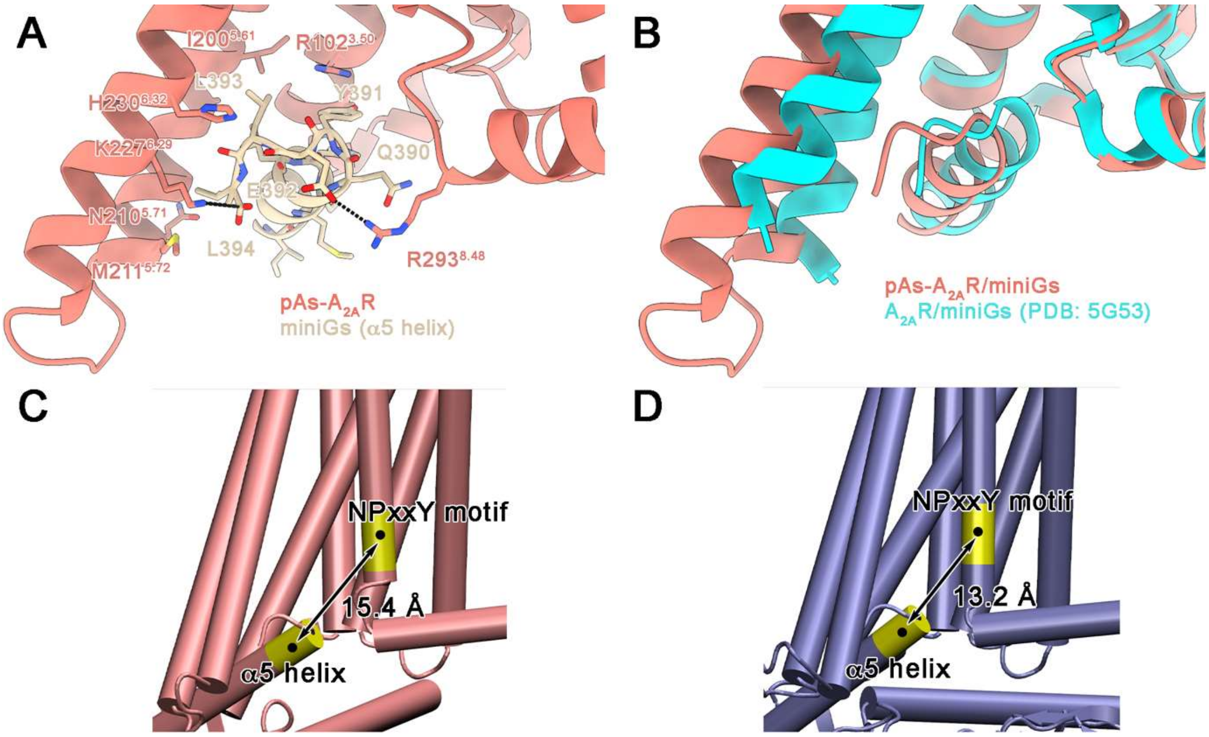

Figure S19. pAs-A$_{2A}$R/G protein binding mode. A) Binding mode of the G$_{\alpha}$'s Cα5 helix (yellow) at the intracellular binding site of A$_{2A}$R pAs (salmon) identified by molecular dynamics simulations. B) Superimposition of the pAs-G protein complex (salmon) with the experimental structure with PDB code 5G53 (light blue). C-D) Distance between the A$_{2A}$R NPxxY motif and the G$_{\alpha}$'s Cα5 helix measured in the pAs/G protein and the experimental A$_{2A}$R/G protein complexes, respectively.

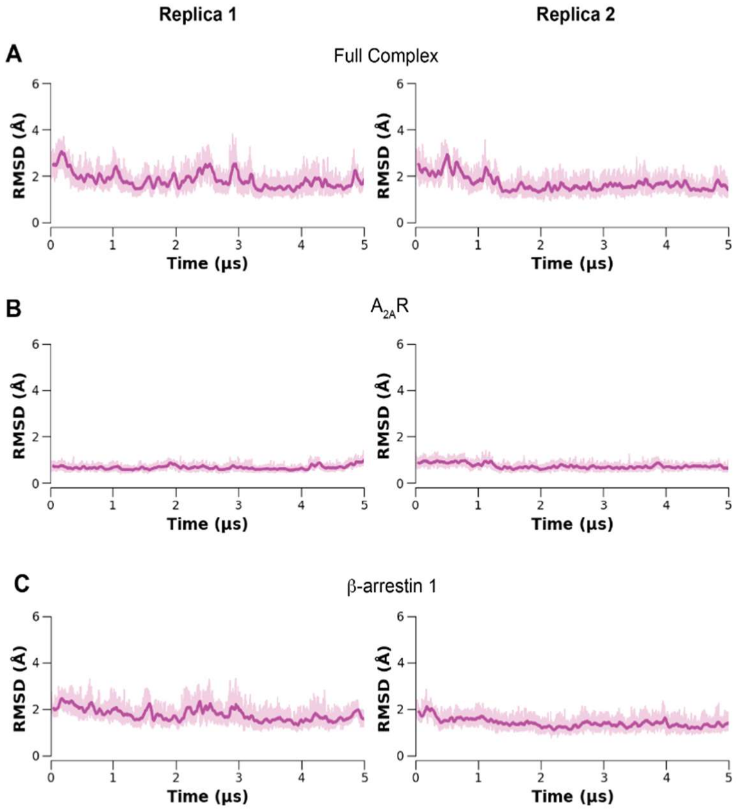

Figure S20. Structural stability of the pAs-A$_{2A}$R/β-arrestin 1 complex. The RMSD values were computed over the two independent 5 microseconds-long MD simulations (left and right column,

respectively) using the Cα atoms of the entire complex (A), the A_{2A} receptor (B) and β-arrestin with respect to their average conformation. The bolded lines show a RMSD value smoothed with a rolling window of 5 ns, whereas the actual fluctuations are shown with a slight transparency.

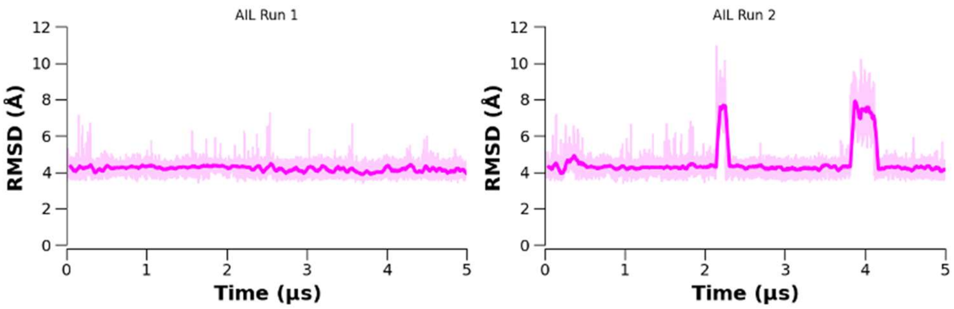

Figure S21. Stability of the Activating Ionic Lock (AIL) interaction. The RMSD values were computed over the two independent 5 microseconds-long MD simulations (left and right column, respectively) on the predicted pAs-A_{2A}R/β-arrestin 1 complex. The AIL is computed as distance between the Cδ of $E^{6.30}$ and the Cz of $R^{5.66}$. The bolded lines show a RMSD value smoothed with a rolling window of 5 ns, whereas the actual fluctuations are shown with a slight transparency.

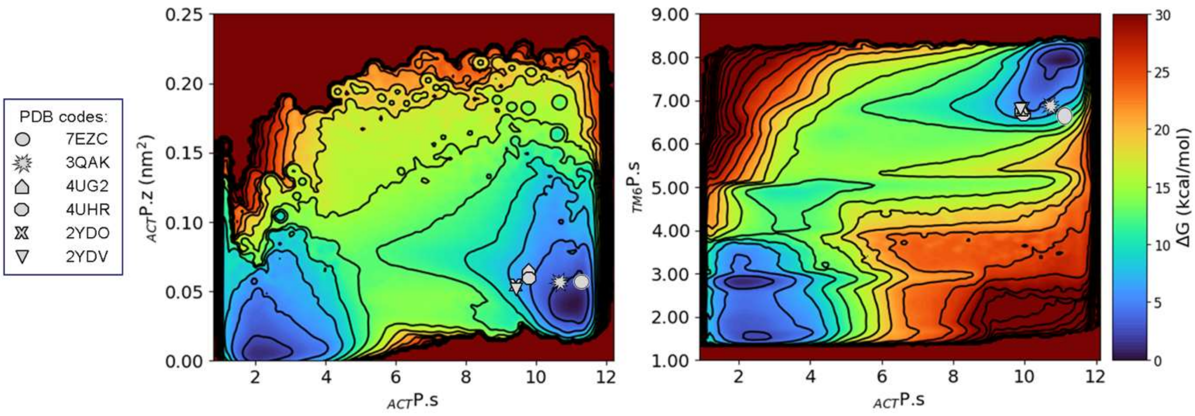

Figure S22. Projection of A_{2A}R experimental structures onto the FES of the A_{2A}R/NECA system. Projection of all the agonist-bound experimental structure (gray shapes) of the *uncoupled* A_{2A}R on the activation free energy surfaces predicted in the present work for the NECA-bound system.

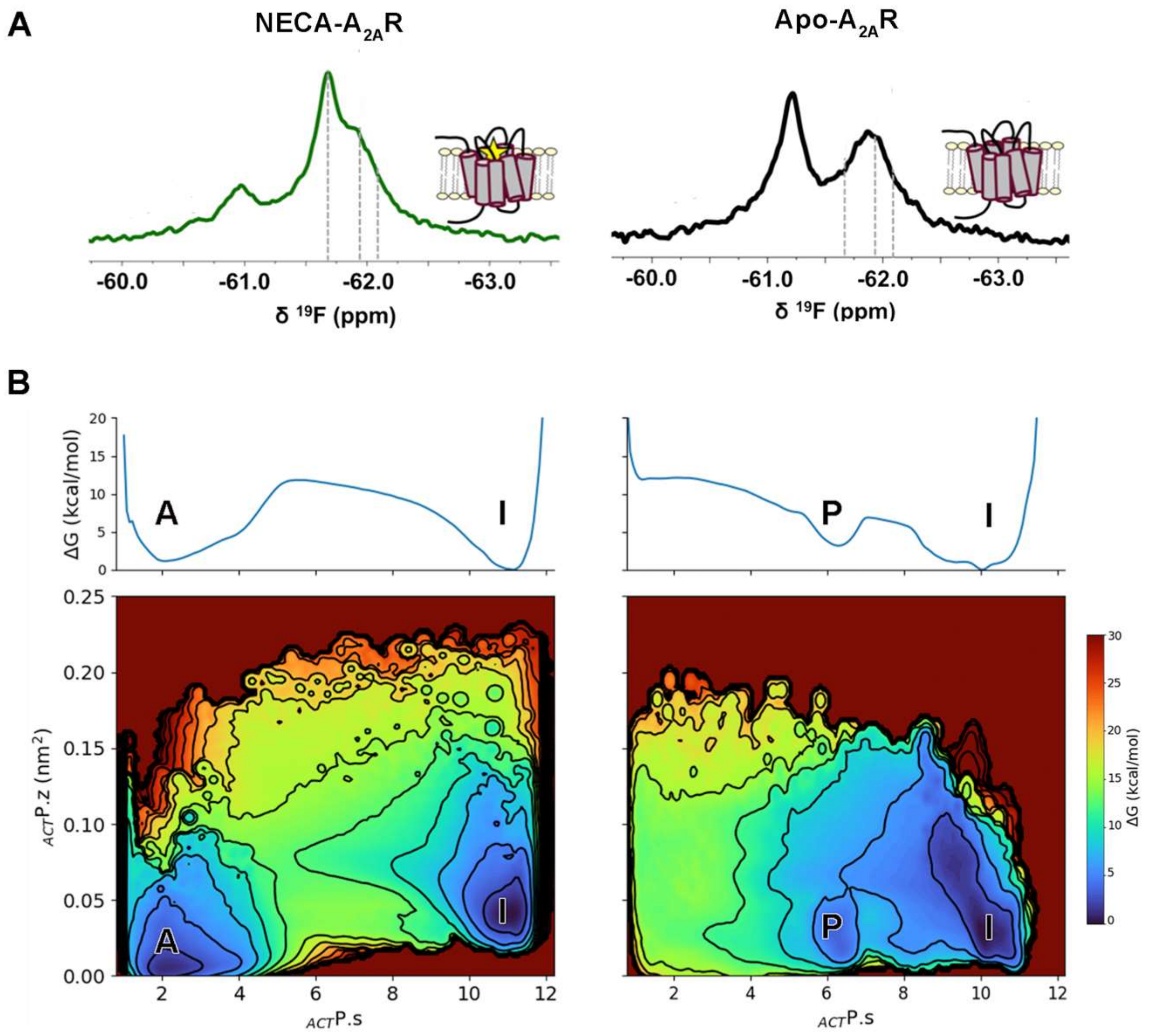

Figure S23. Comparison between ^{19}F-NMR spectra and free energy surfaces of A$_{2A}$R. A) ^{19}F NMR spectra of A$_{2A}$R-V229C in *apo* conditions and in presence of NECA reported by Prosser and coworkers (*Cell,* **2021**, 184, 1884–1894). B) Activation free energy surfaces of the *NECA-bound*, *apo*, and *ZMA-bound* A2AR as a function of the $_{ACT}$P.s and $_{ACT}$P.z collective variables computed by means of PCV-MetaD.
